# Self-Powered Sensing in Wearable Electronics—A Paradigm Shift Technology

**DOI:** 10.1021/acs.chemrev.3c00305

**Published:** 2023-10-23

**Authors:** Wei Tang, Qijun Sun, Zhong Lin Wang

**Affiliations:** † CAS Center for Excellence in Nanoscience, Beijing Institute of Nanoenergy and Nanosystems, Chinese Academy of Sciences, Beijing 100083, China; ‡ School of Nanoscience and Technology, University of Chinese Academy of Sciences, Beijing 100049, China; § Institute of Applied Nanotechnology, Jiaxing, Zhejiang 314031, P.R. China; ¶ Yonsei Frontier Lab, Yonsei University, Seoul 03722, Republic of Korea; ⊥ Georgia Institute of Technology, Atlanta, Georgia 30332-0245, United States

## Abstract

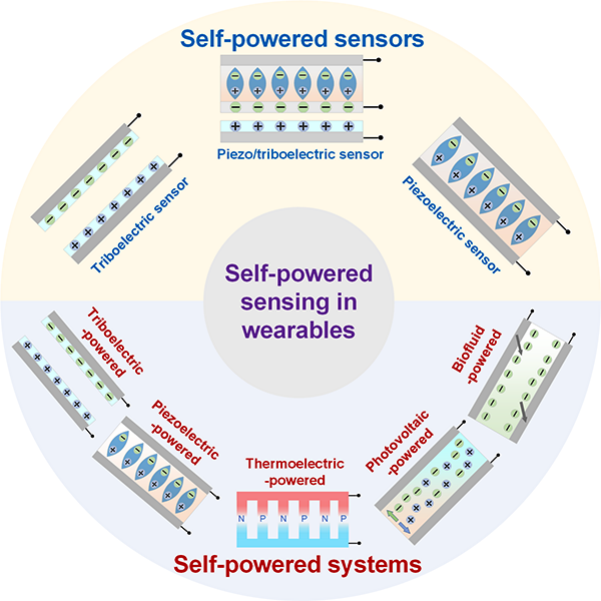

With the advancements in materials science and micro/nanoengineering, the field of wearable electronics has experienced a rapid growth and significantly impacted and transformed various aspects of daily human life. These devices enable individuals to conveniently access health assessments without visiting hospitals and provide continuous, detailed monitoring to create comprehensive health data sets for physicians to analyze and diagnose. Nonetheless, several challenges continue to hinder the practical application of wearable electronics, such as skin compliance, biocompatibility, stability, and power supply. In this review, we address the power supply issue and examine recent innovative self-powered technologies for wearable electronics. Specifically, we explore self-powered sensors and self-powered systems, the two primary strategies employed in this field. The former emphasizes the integration of nanogenerator devices as sensing units, thereby reducing overall system power consumption, while the latter focuses on utilizing nanogenerator devices as power sources to drive the entire sensing system. Finally, we present the future challenges and perspectives for self-powered wearable electronics.

## 1 Introduction

Humans increasingly depend on wearable sensors to monitor their physical and physiological conditions,^1−5^ thereby enhancing the quality of life in the digitalized and intelligent world. For example, wearable or attachable devices can monitor heart rate, electrocardiograph signals,^6−8^ providing crucial information for diagnosing heart diseases. Skin temperature can be obtained through flexible devices,^9−11^ serving as direct indicators of certain diseases related to immune response. Body motion analysis can reveal health conditions associated with orthopedics, muscles, or neurological disorders.^12−14^ Additionally, attachable electronic patches can monitor sweat to indicate metabolic conditions.^15−17^ Sensing devices have also been developed for monitoring respiration,^18,19^ blood,^20,21^ and skin^22^ to provide early warning signs for related diseases.

Despite significant advancements in benchside research over the past decade, the market adoption of flexible sensors remains limited due to several bottlenecks that hinder their maturation, one of which is power supply. Specifically, incorporating a battery in a wearable sensor system imposes constraints on its volume and weight, thereby limiting its potential applications. Moreover, when the battery is depleted, continuous monitoring is disrupted, potentially affecting the accuracy of estimations and consequently reducing user engagement. Thus, the development of wearable systems with innovative power supply technologies is of paramount importance.

**Figure 1:**
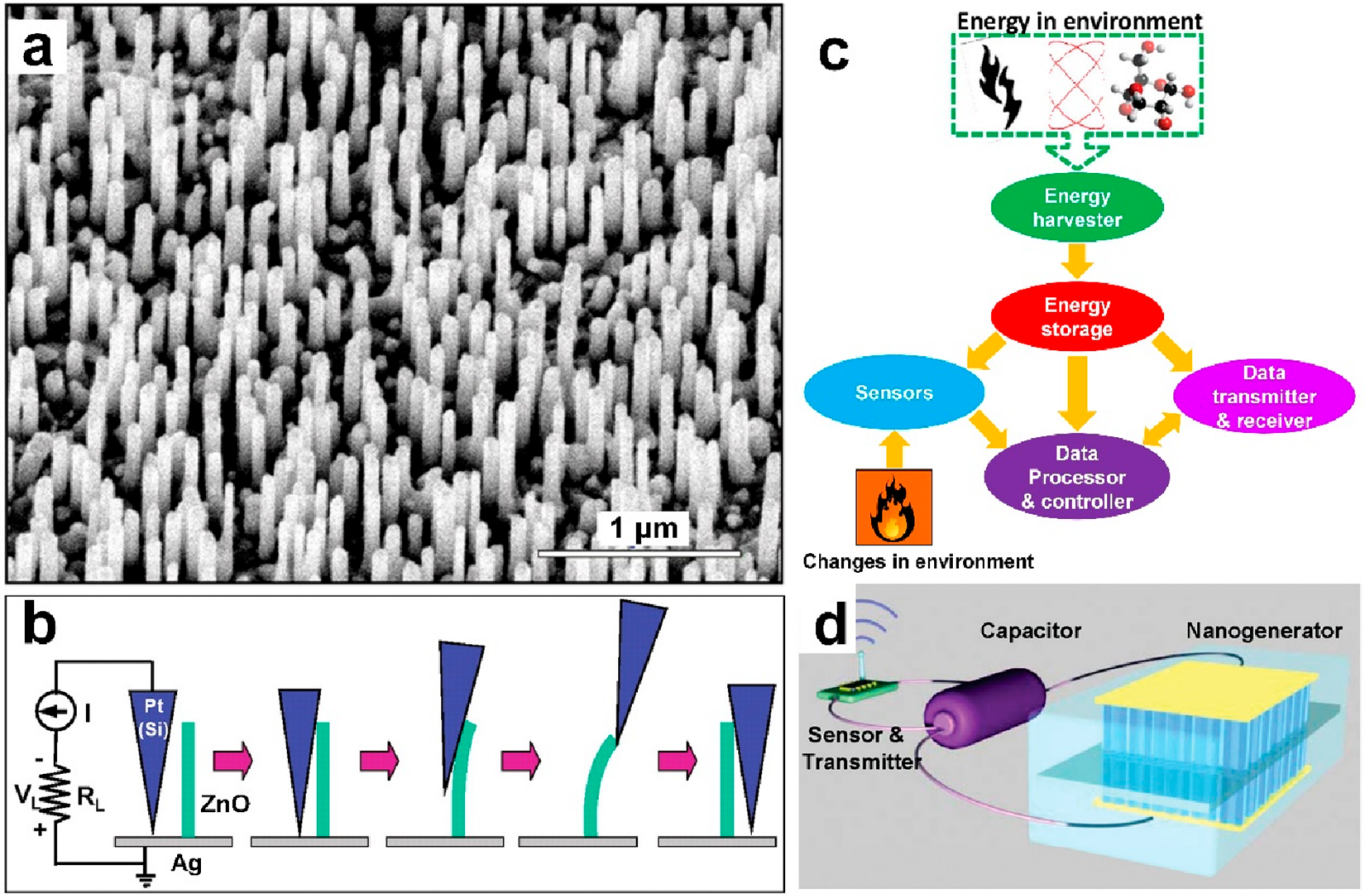
Emergence of the self-powered concept. (a) ZnO nanowires and (b) electricity generation by using an AFM tip to bend a ZnO nanowire. Reproduced with permission from ref (24). Copyright 2006 AAAS. (c) Sketch of the self-powered nanosystem. Reproduced with permission from ref (25). Copyright 2010 Elsevier Ltd. (d) Self-powered nanosystem with wireless data transmission. Reproduced with permission from ref (26). Copyright 2012 Wiley-VCH.

In 2008, Wang introduced the concept of self-powered nanosystems^23^ based on the developed piezoelectric nanogenerator, which is capable of converting the mechanical trigger from an AFM tip into an electrical output, as reported in 2006 (Figure 1a and 1b).^24^ The self-powered nanosystem comprises a sensor, a data processing and transmission circuit, and an energy harvesting and storage unit, as depicted in Figure 1c and 1d. The energy harvester can convert ambient mechanical, thermal, chemical, and even solar energy into electricity, which then powers the subsequent sensing processes, including data acquisition, processing, and transmission.

**Figure 2:**
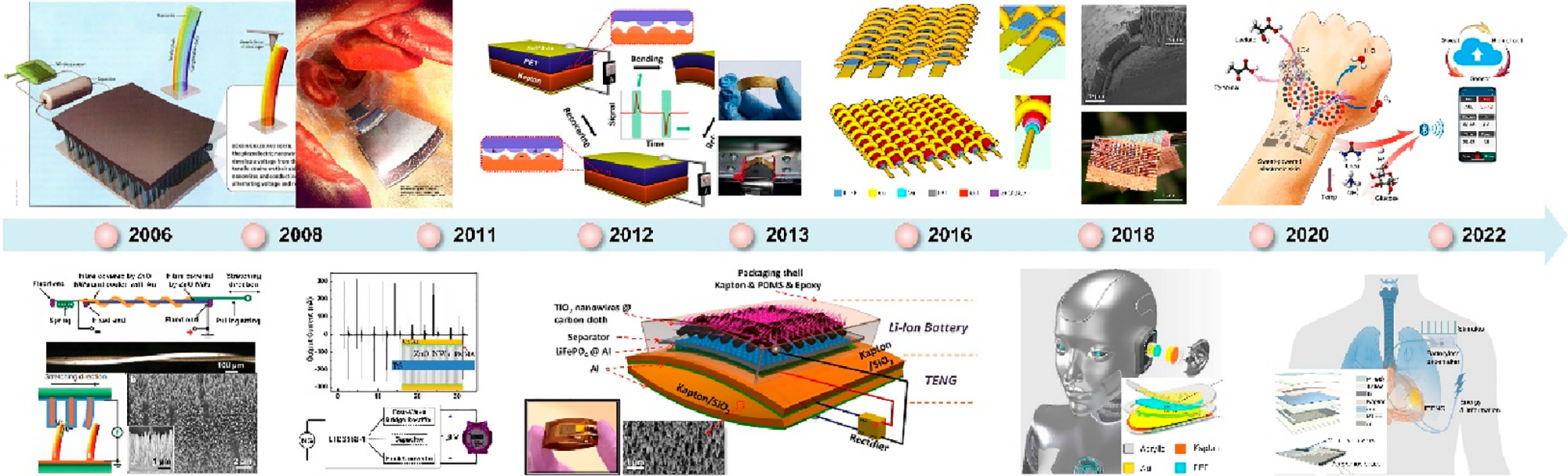
Rapid development of self-powered nanosystems and their applications in wearable electronics. Piezoelectric nanogenerator in 2006. Reproduced with permission from ref (24). Copyright 2006 AAAS. Self-powered nanosystems in 2008 and 2011. Reproduced with permission from ref (27). Copyright 2011 WILEY-VCH. Triboelectric nanogenerator in 2012. Reproduced with permission from ref (28). Copyright 2012 Elsevier Ltd. Self-charging power unit in 2013. Reproduced with permission from ref (29). Copyright 2013 American Chemical Society. Hybrid power suit in 2016. Reproduced with permission from ref (30). Copyright 2016 Springer Nature. Self-powered sensors in 2018. Reproduced with permission from ref (31). Copyright 2018 AAAS. Self-powered skin electronics powered by sweat in 2020. Reproduced with permission from ref (33). Copyright 2020 AAAS. Symbiotic cardiac pacemaker. Reproduced with permission from ref (32). Copyright 2019 The Authors.

Subsequently, numerous self-powered prototypes have been developed. As illustrated in Figure 2, a nanogenerator based on piezoelectric materials was reported in 2006.^26^ This was followed by the proposal of a self-powered nanosystem in 2008.^23−25^ In 2011, a fiber-based piezoelectric nanogenerator featuring thousands of ZnO nanowires was fabricated, and the first self-powered electronic watch powered by a ZnO–nanowire array was reported.^27^ In 2012, a novel mechanical energy harvesting approach based on triboelectrification and electrostatic induction emerged, named a triboelectric nanogenerator (TENG).^28^ Due to its high output performance, self-powered nanosystems have advanced further. For example, a self-powered energy cell consisting of a TENG and a Li-ion battery was reported in 2013.^29^ In 2016, a combination of TENGs and solar cells was woven into fabric, serving as a wearable power supply.^30^ In 2018, employing a TENG as a self-powered sonic sensor was proposed, with the device functioning as a hearing aid for humans or robots.^31^ In 2020, a self-powered sweat sensing system powered by human motion energy was demonstrated. Furthermore, a symbiotic cardiac pacemaker was unveiled in 2021,^32^ enabling the pacemaker to be driven by the host organism’s own heartbeat.

**Figure 3:**
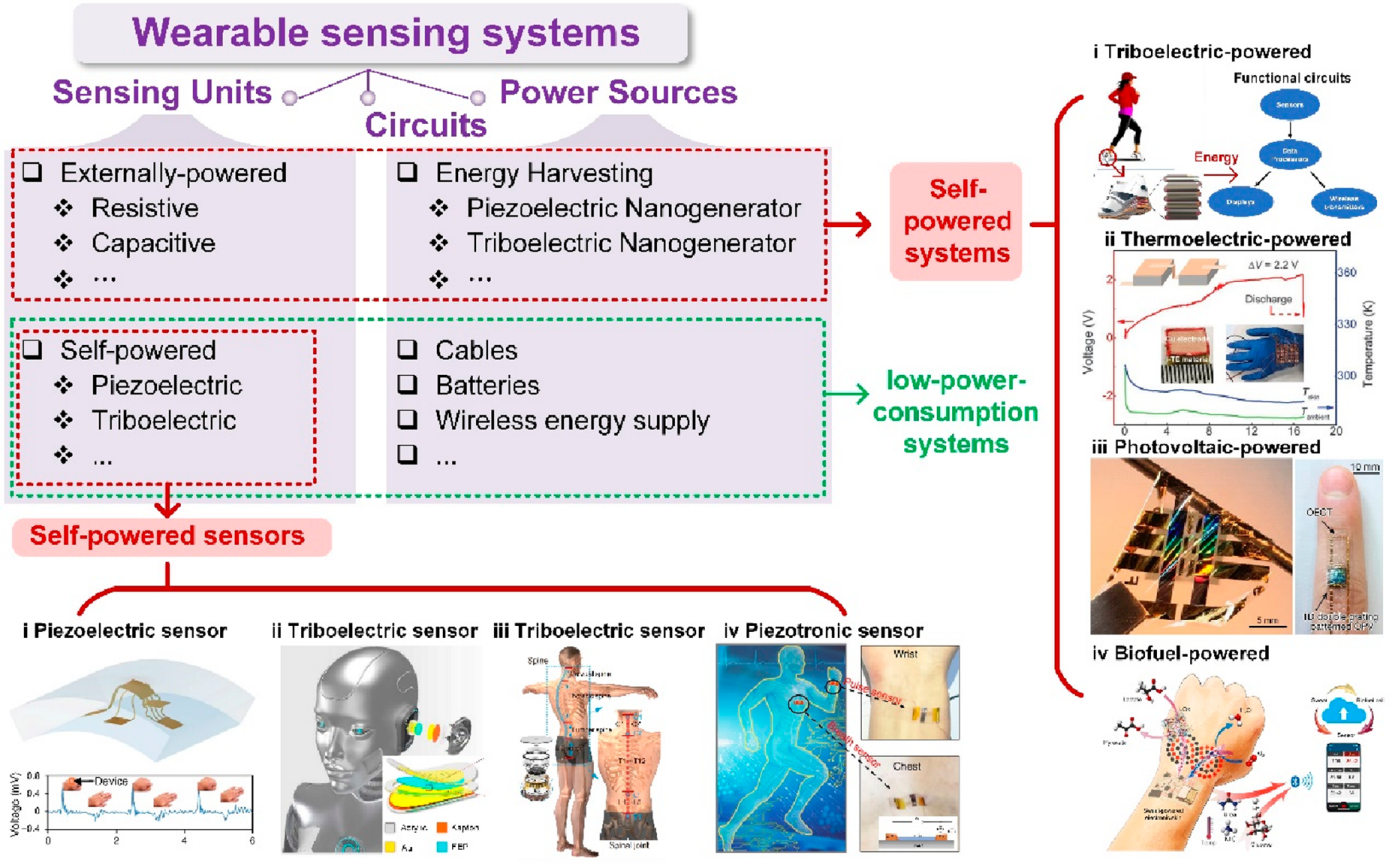
Self-powered sensors and self-powered systems in wearable electronics: self-powered sensors employ energy harvesters as sensing units. (i) Piezoelectric sensor. Reproduced with permission from ref (66). Copyright 2019 Springer Nature. (ii) Triboelectric sensor. Reproduced with permission from ref (31). Copyright 2018 AAAS. (iii) Triboelectric sensor. Reproduced with permission from ref (118). Copyright 2021 The Authors. (iv) Piezotronic sensor. Reproduced with permission from ref (130). Copyright 2019 American Chemical Society. Self-powered systems employ energy harvesters as power sources. (i) Triboelectric powered. Reproduced with permission from ref (76). Copyright 2015 The Authors. (ii) Thermoelectric powered. Reproduced with permission from ref (192). Copyright 2020 AAAS. (iii) Photovoltaic powered. Reproduced with permission from ref (234). Copyright 2018 Springer Nature. (iv) Biofuel powered. Reproduced with permission from ref (33). Copyright 2020 AAAS.

Over the past decade, various innovative technologies and self-powered prototypes have been developed, Figure 3, making self-powered technology promising for wearable applications. As researchers worldwide devote significant effort to this field, two main research strategies have emerged: one involves using nanogenerators as power sources to drive sensing units and processing circuits, thereby constructing complete self-powered systems; the other entails employing energy harvesters as sensing units, serving as self-powered sensors, and then integrating them with processing circuits and external power sources to create low-power-consumption systems. The former approach benefits from mature sensing devices, but its drawback lies in the depletable nature of the generated power from current powering techniques, which limits the system’s functionalities. Conversely, the latter approach enables the use of self-powered sensors to extend the overall system’s battery life, directly integrate with mature processing and power circuits, and in some scenarios enhance the signal-to-noise ratio due to the self-generating-signal ability.^34^ However, this method requires external power sources and the development of additional self-powered sensors. Therefore, in the subsequent sections, we will discuss both strategies and present other innovative powering techniques suitable for wearable applications.

## 2 Self-Powered Wearable Sensors

In this section, we present typical self-powered wearable sensors based on piezoelectric, triboelectric, piezotronic, and tribotronic effects.

### 2.1 Piezoelectric Sensors

#### 2.1.1 Mechanism

**Figure 4:**
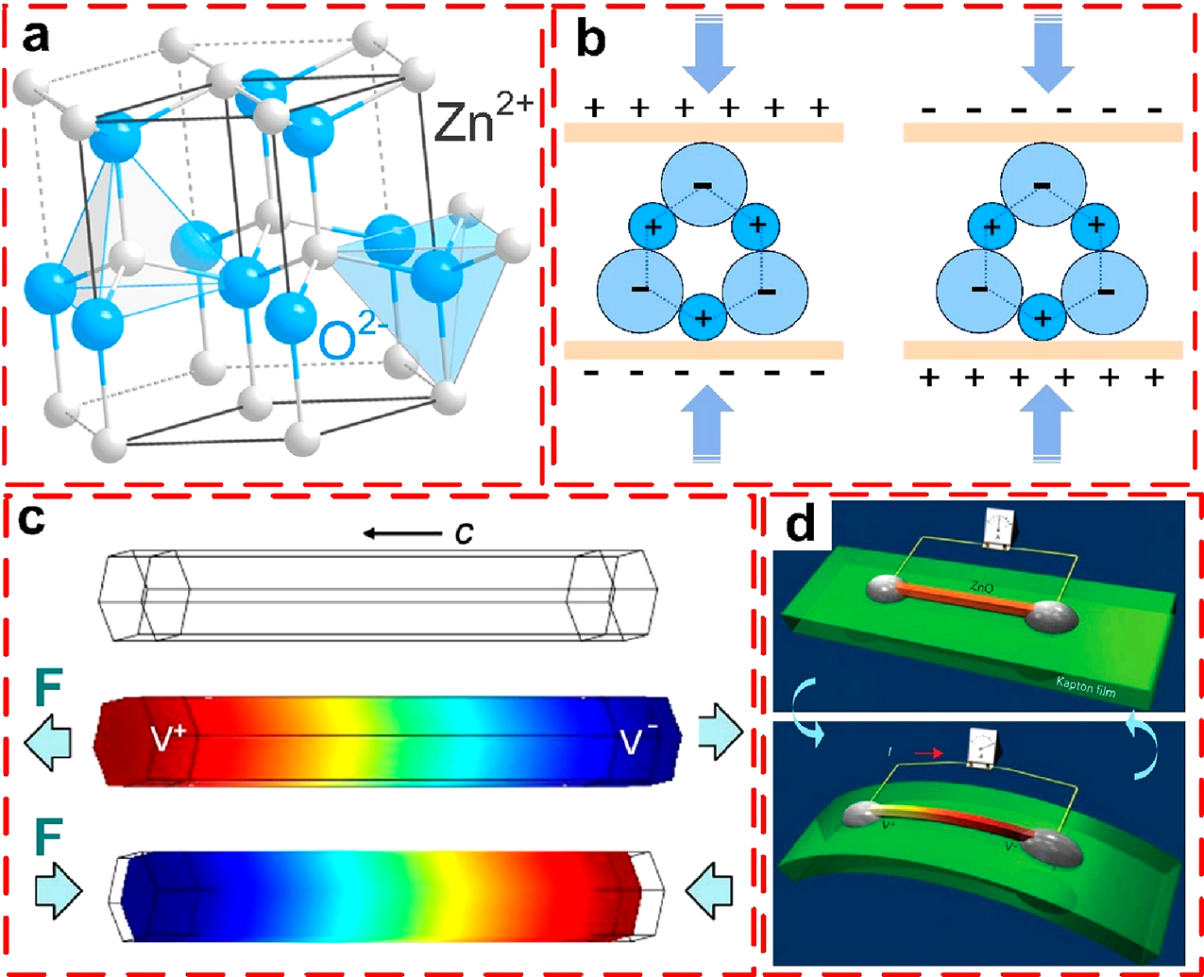
Sketch of the piezoelectric mechanism. (a) Wurtzite-structured ZnO. (b) Piezopotential in tension and compression. (c) Numerical calculation of the piezoelectric potential distribution in a ZnO nanowire under axial strain. Reproduced with permission from ref (43). Copyright 2009 AIP Publishing LLC. (d) Potential of a piezoelectric nanowire under bending. Reproduced with permission from ref (48). Copyright 2008 Springer Nature.

Piezoelectricity has been reported for a long time.^35,36^ It stands for the electricity generation due to the breaking of the material structure’s central symmetry under pressure. It has been widely exploited in applications, such as the sonic production^37^ and detection,^38^ inkjet printing,^39^ scanning microscopes,^40,41^ high-voltage electricity generation,^42^ etc. The first nanogenerator is based on the piezoelectricity of zinc oxide (ZnO). Under an external force, the central symmetry in the ZnO crystal structure is broken, forming a piezopotential. For example, in the wurtzite structure of a ZnO crystal, Zn^2+^ and O^2–^ are stacked layer-by-layer along the *c* axis^43^ (see Figure 4a), and the charge center of the cations and anions overlap at this stage. When the structure is deformed under an external force, the charge centers are separated and thus form an electric dipole, resulting in a piezopotential (Figure 4b). Therefore, free electrons are driven to flow through the external circuit in order to screen the piezopotential and achieve electrostatic equilibrium. That is the mechanics–electricity conversion process (see Figure 4c).^44^ If the external force is periodically applied, the nanogenerator will continuously output (Figure 4d). Based on this working principle, numerous forms of piezoelectric devices are reported.^45−48^

#### 2.1.2 Piezoelectric Materials for Wearables

**Figure 5:**
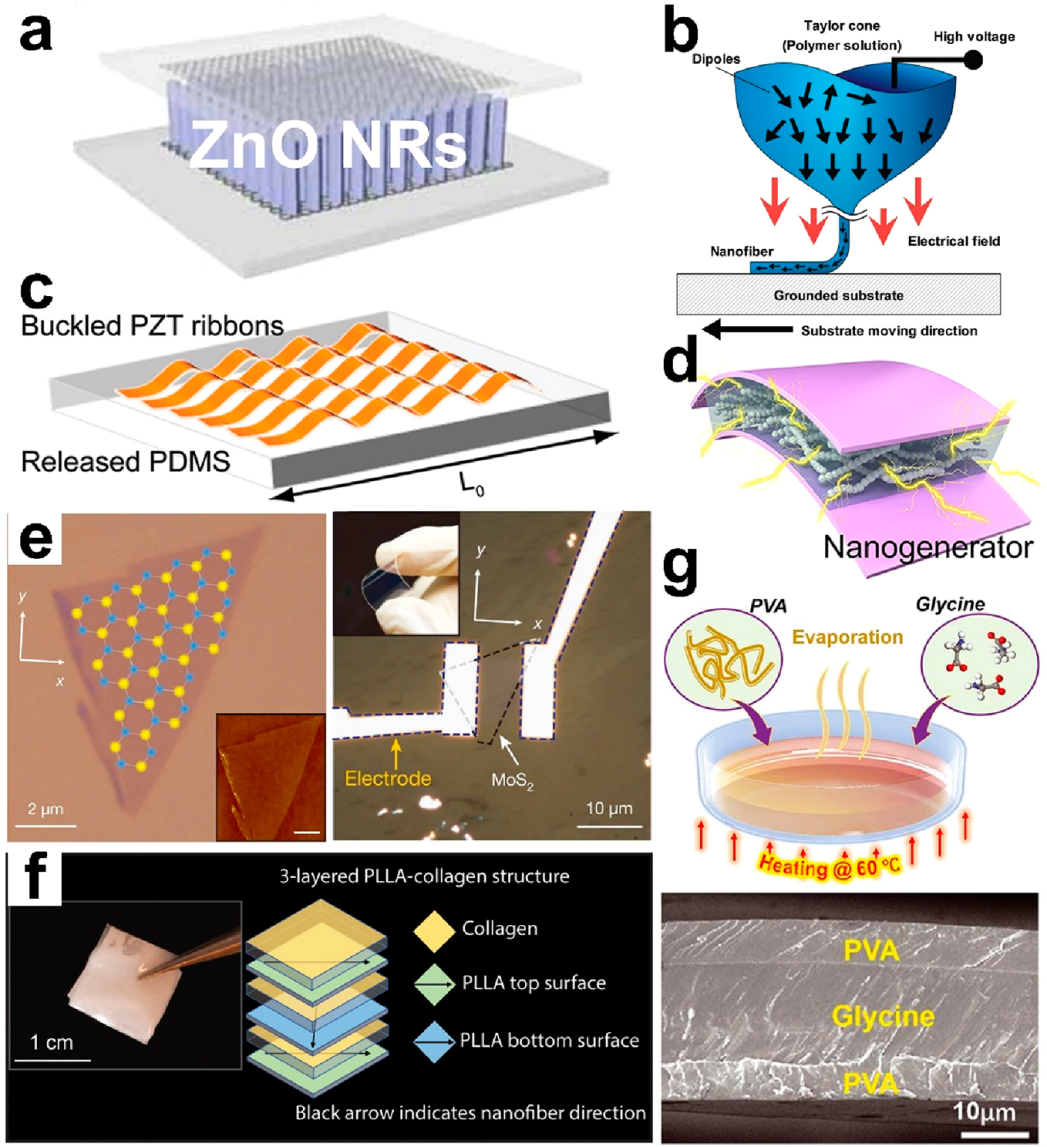
Common piezoelectric materials for wearable applications. (a) ZnO nanorod array. Reproduced with permission from ref (58). Copyright 2010 WILEY-VCH. (b) PVDF fibers. Reproduced with permission from ref (51). Copyright 2010 American Chemical Society. (c) Buckled PZT ribbons. Reproduced with permission from ref (54). Copyright 2011 American Chemical Society. (d) BaTiO_3_-doped dielectric materials. Reproduced with permission from ref (55). Copyright 2013 American Chemical Society. (e) Two-dimensional materials of MoS_2_. Reproduced with permission from ref (61). Copyright 2014 Springer Nature. (f) Implantable PLLA. Reproduced with permission from ref (57). Copyright 2022 AAAS. (g) Wafer-level bio-organic glycine. Reproduced with permission from ref (56). Copyright 2021 AAAS.

The integration of sensing elements with the soft and curvilinear surfaces of the human body requires attention to materials design to obtain seamless and breathable interfaces, which ensures devices’ robustness and wearers’ comfort during daily motions.^48,49^ Therefore, flexible/stretchable materials are required. Traditional piezoelectric materials are piezoelectric ceramics, e.g., lead zirconate titanate (PZT).^50^ Although it possesses a high piezoelectric coefficient (PC) of 218.7 pC (d33), the toxicity of ingredients and low flexibility restrict its wearable applications. Hence, soft piezoelectric materials have been rapidly developed. Figure 5a shows an array of ZnO nanorods capsulated by soft jelly, such as polydimethylsiloxane (PDMS) or Ecoflex. Under bending or stretching, the jelly compresses the ZnO nanorods, delivering electricity output. Poly(vinylidene fluoride) (PVDF), as an organic material with inherent flexibility, is also widely used as the piezoelectric material. It can be fabricated as fibers and textiles, which are suitable for wearable applications. Lin reported PVDF fibers fabricated via electrospinning (Figure 5b) with in situ mechanical stretching and electrical poling to produce piezoelectric properties.^51,52^ Besides, diversiform piezoelectric fibers, combining wearability and piezoelectricity, have been discussed in the literature (including inorganic ceramics or organic polymers).^53^ Another approach to obtain soft piezoelectric materials is softening traditional piezoelectric ceramics. Figure 5c shows a buckled PZT ribbon array, which makes the whole film retractable. Compared with PVDF, it exhibits 10-times higher output; however, the reported largest strain is 8%.^54^Figure 5d shows the barium carbonate-doped soft dielectric materials.^55^ The challenge lies in how to align the barium carbonate *c* axis to achieve a high PC value. Furthermore, as more new materials emerge, two-dimensional MoS_2_ shows its piezoelectric property and forms a soft and transparent device, as shown in Figure 5e.^56^ Besides, poly(l-lactic acid) (PLLA) and poly(vinyl alcohol) (PVA)/glycine/PVA were recently proposed due to their excellence in softness, piezoelectric property, and biocompatibility (Figure 5f and 5g).^56,57,59,60^ Notably, PVA is employed to promote the crystallization of glycine due to the hydrogen bonding at the PVA–glycine interface, which results in a large-scale generation of a piezoelectric film, with water-soluble and biodegradable properties.

#### 2.1.3 Devices and Applications

**Figure 6:**
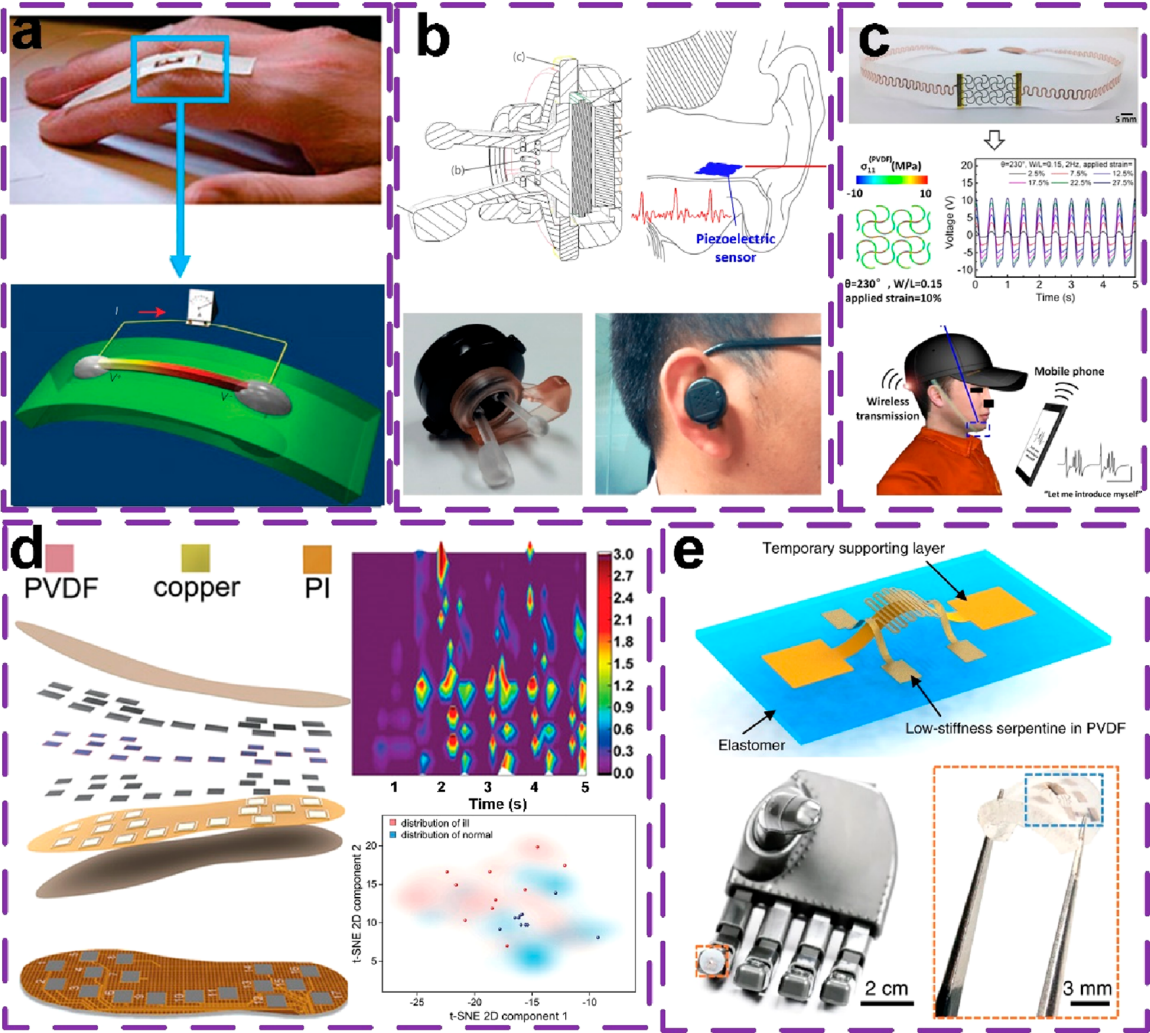
Piezoelectric sensors for wearable applications. (a) Finger motion sensor. Reproduced with permission from ref (62). Copyright 2009 American Chemical Society. (b) Sonic wave sensor. Reproduced with permission from ref (63). Copyright 2015 MDPI. (c) Vibration sensor. Reproduced with permission from ref (64). Copyright 2019 American Chemical Society. (d) Plantar pressure sensors. Reproduced with permission from ref (65). Copyright 2022 WILEY-VCH. (e) Three-dimensional touch sensor. Reproduced with permission from ref (66). Copyright 2019 Springer Nature.

In this section, we show examples of wearable sensors based on a piezoelectric mechanism. Figure 6a shows a finger bending monitoring device made up of a ZnO nanowire on a flexible substrate with an output around 0.1 V.^62^ And, the sensor output can be enhanced by serially connecting the devices. Figure 6b presents a sonic sensing application based on PVDF, where the sensing film will detect the sonic signals.^63^Figure 6c shows the application of using a piezoelectric device as the phonation sensor to help dumb people. The serpentine mesh layout was employed to achieve the desired stretchability instead of modifying the intrinsic mechanical properties of PVDF.^64^Figure 6d shows a smart insole, where the piezoelectric film of PVDF serves as a pressure sensor to detect the wearer’s foot pressure distribution.^65^ It is useful to assist doctors in the diagnosis of some orthopedic disorders, e.g., lumbar stenosis. In this work, the authors collected time-varying pressure data during patients walking and then employed machine learning analysis to evaluate and predict the patients’ disorders. Eventually, the system can recognize the patients from the healthy group and evaluate the patients’ postoperative recovery status. Figure 6e illustrates the piezoelectric sensor’s application in robotics. Notably, Han et al. utilized a three-dimensional processing technique to fabricate a stereostructure. Therefore, the sensor can detect multidirectional force trigger, making it a high-sensitivity touch sensor.^66^

### 2.2 Triboelectric Sensor

#### 2.2.1 Mechanism

**Figure 7:**
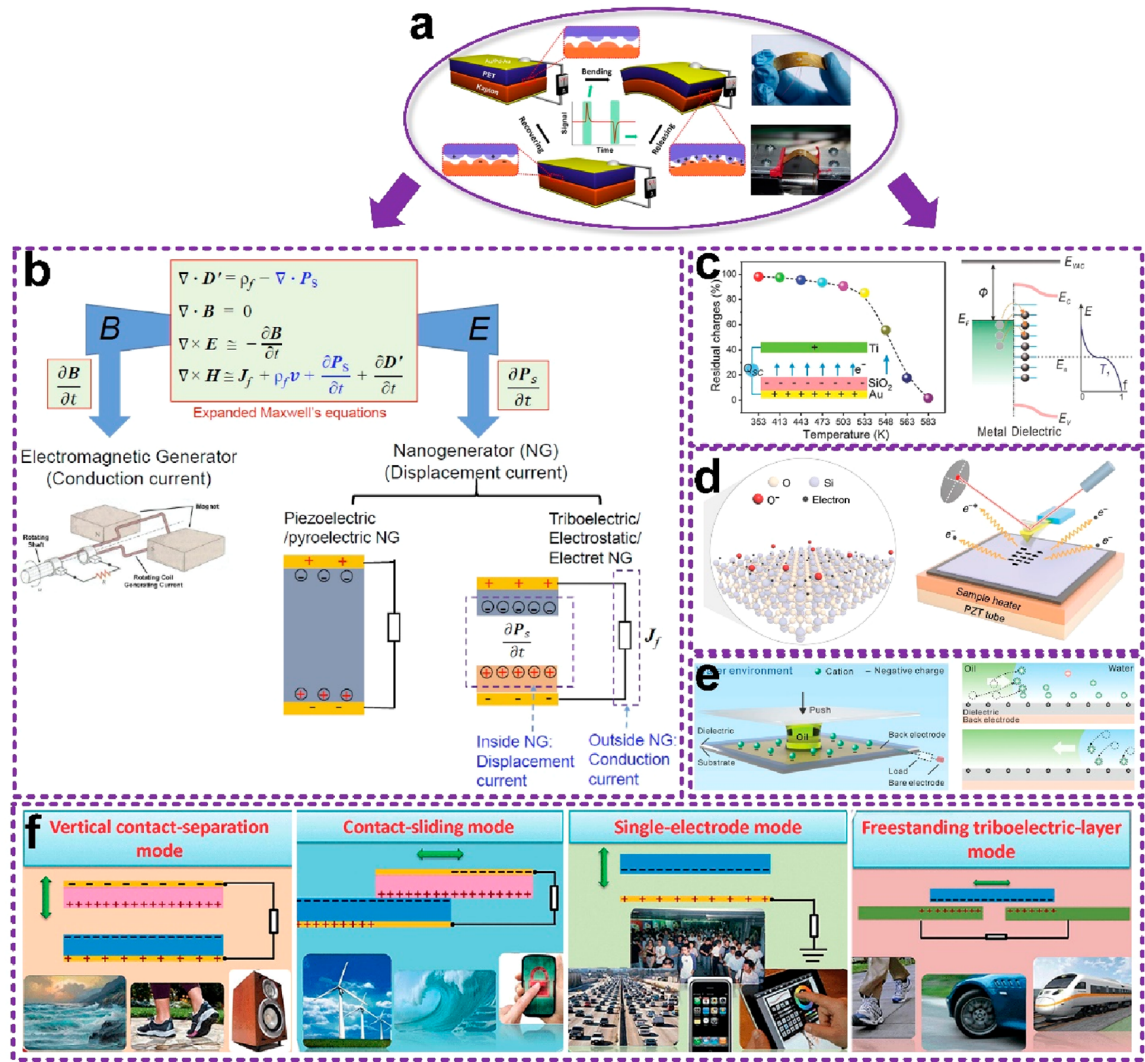
Mechanism of triboelectric nanogenerators. (a) First triboelectric nanogenerator. Reproduced with permission from ref (28). Copyright 2012 Elsevier Ltd. (b) Theory of the triboelectric devices. Reproduced with permission from ref (82). Copyright 2016 Elsevier Ltd. (c) Mechanism of tribocharges’ transfer between the interface pair. Reproduced with permission from ref (88). Copyright 2018 WILEY-VCH. (d) Electron/ion transfer both exist in the solid–water contact. Reproduced with permission from ref (91). Copyright 2020 The Authors. (e) Charge transfer during liquid–liquid contact. Reproduced with permission from ref (94). Copyright 2022 WILEY-VCH. (f) Four working modes of TENG devices. Reproduced with permission from ref (97). Copyright 2014 Elsevier Ltd.

The triboelectric nanogenerator was first proposed in 2012.^28^ It combined contact electrification and electrostatic induction, converting mechanical motions into electricity output.^67−69^ Generally, when two materials come in contact, due to the difference in the electron affinities, electrons are inclined to transfer from the one with lower affinity to the other with higher affinity; therefore, polarization is generated across the interface. Afterward, when the two materials separate or approach periodically, the electric field varies and causes electrons to move back and forth between the two electrodes located behind the contacting materials via external wires/circuits. Thus, alternative current is obtained (Figure 7a, more details about the working principle of the TENG can be found in the literature^70−72^). The output could be employed as a power supply^73−76^ or as a sensing signal.^77−80^ In this section, we focus on the sensing performance of the TENG.

It is also worth noting that both TENGs and PENGs are on the basis of the dielectric materials’ polarization. The physical theory is derived from the classical Maxwell equation, specifically the displacement current *∂P*_s_/*∂t*, as shown in Figure 7b.^81^ The PENG output is determined by the variation of the displacement current inside the piezoelectric material, whereas that of the TENG is determined by the variation of the displacement current during the interface of two contacting materials. Specifically, the output of the TENG is characterized by a term , where ***P***_s_ is mainly due to the existence of the surface charges and the relative movement of the objects as driven by mechanical motion. In general, the conventional Maxwell equations are for media whose boundaries and volumes are fixed and stationary. But, for the cases that involve moving objects, such as the case in the TENG, the equations have to be expanded. Starting from the integral forms of the four physics laws, Wang derived the expanded Maxwell equations in differential forms for slow moving objects (*v* ≪ *c*). The Maxwell equations for a mechano-driven slow-moving media system are given by^82−84^
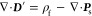
1a
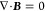
1b
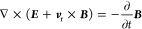
1c
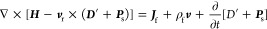
1d

The moving velocity of the unit charge inside the medium is split into two components: the moving velocity ***v*** of the moving reference frame and the relative moving velocity (***v***_r_) of the point charge inside the medium with respect to the moving reference frame. These equations are most useful for describing the electromagnetic behavior of moving media with acceleration, and they are fundamental for dealing with the coupling among mechano–electric–magnetic multifields and the interaction. The expanded equations are the most comprehensive governing equations including both electromagnetic interaction and power generation as well as their coupling for a TENG. Of course, the applications of Maxwell’s equations for a mechano-driven system are more general, and their application fields are way beyond the cases for a TENG.

Another interesting research focus of TENGs is the origin of the transferred charge. In the past, an electron transfer mechanism dominated, and theories based on the metals’ work function were adopted to explain the variety of materials’ electrification abilities.^85^ Afterward, Whitesides reported an ion transfer mechanism in cases of solid–liquid.^86^ Meanwhile, there are some other opinions which point out that the contact process will cause mass transfer and then lead to electrification.^87^ Since 2018, Wang has published a series of works on this topic. It was found that in the case of solid–solid contact, electron transfer is dominant as the as-generated charge recession follows the hot electron emission rule, which are both proved at macro- and microscales^88−90^ (Figure 7c). When the contact happens in water and a solid, both electron and ion transfer exist^91,92^ (Figure 7d), where the ratio is determined by the contact angle of the solid. Other works about the charge transfer mechanism have been discussed in detail in the references, including liquid–liquid contact,^93,94^ gas–solid contact,^95^ etc. (Figure 7e).

As for the TENG device, it has been widely investigated, and four modes have been developed, i.e., contact-separation mode, sliding mode, single-electrode mode, and free-standing mode, as shown in Figure 7f.^96^ They are suitable for different application scenarios. For example, the contact-separation mode can sense pressing or triggering, while the sliding mode is suitable for displacement sensation. A single-electrode mode can be used in many cases for its simple structure configuration, but the drawback is its lower interference resistance and signal output. The free-standing mode works similarly to the sliding mode and takes the feature of the facile fabrication process. Many self-powered triboelectric sensors have been designed based on the above four modes.^97−99^

#### 2.2.2 Triboelectric Materials for Wearables

**Figure 8:**
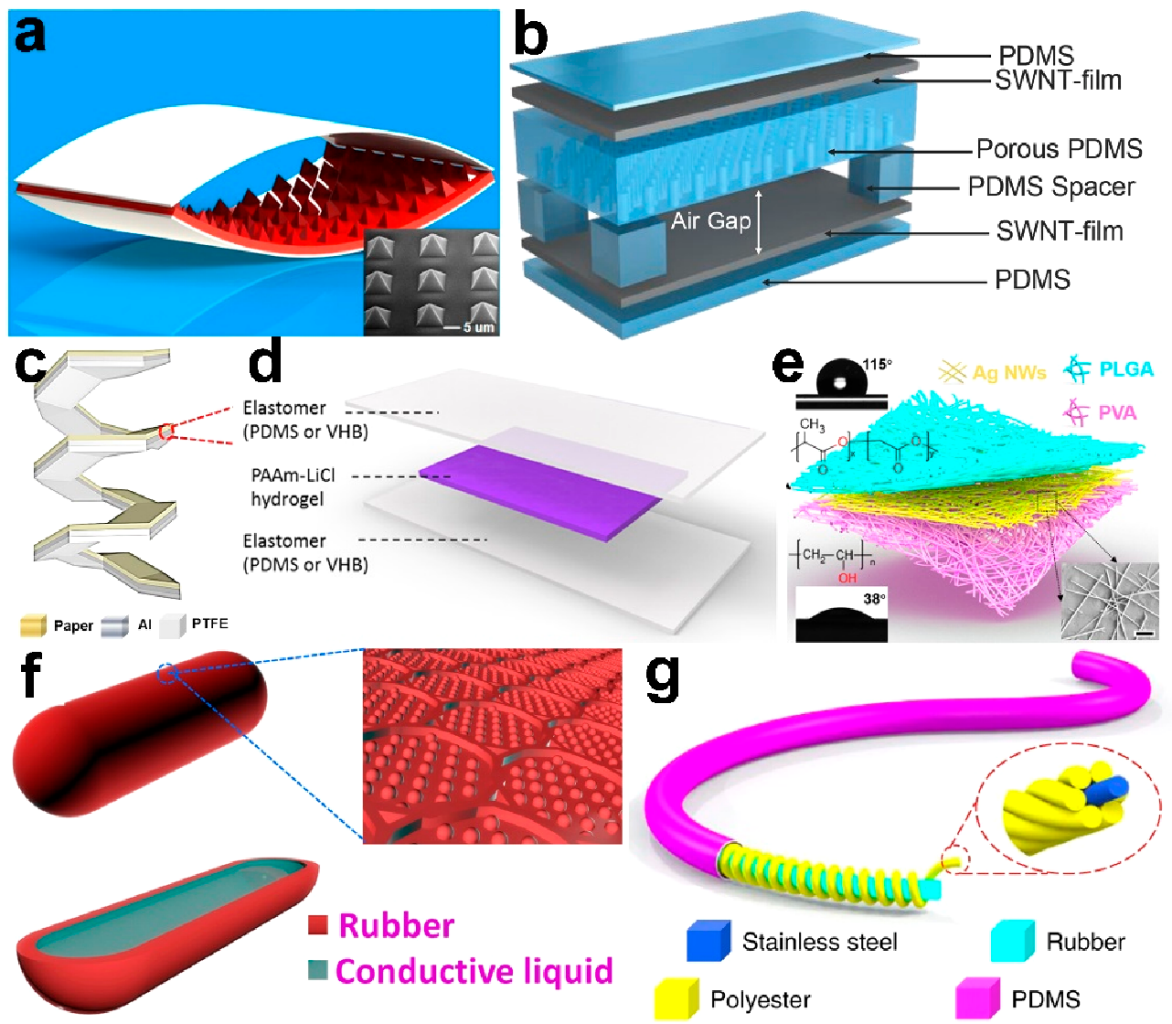
Common triboelectric materials for wearable applications. (a) PDMS with micropatterns. Reproduced with permission from ref (101). Copyright 2015 American Chemical Society. (b) Porous PDMS for TENGs. Reproduced with permission from ref (102). Copyright 2014 American Chemical Society. (c) Paper TENGs. Reproduced with permission from ref (103). Copyright 2015 American Chemical Society. (d) Hydrogel TENGs. Reproduced with permission from ref (108). Copyright 2017 The Authors. (e) TENG devices based on electrospinning materials. Reproduced with permission from ref (110). Copyright 2020 The Authors. (f) TENG devices based on a conductive liquid and rubber. Reproduced with permission from ref (107). Copyright 2016 The Authors. (g) Fiber TENGs. Reproduced with permission from ref (111). Copyright 2020 The Authors.

As for wearable applications, materials are also required to be flexible, stretchable, and lightweight. In this section, we recall common materials used for wearable triboelectric sensors. Among them, polydimethylsiloxane (PDMS) is commonly utilized at the beginning for the mold fabrication, as shown in Figure 8a. Researchers employed the molding process to fabricate various PDMS films, covered by gratings, pillars, pyramids, and even micro–nano dual structures,^77,100,101^ which increases the contact area, also weakens the sticky surface to some degree, and eventually enhances the sensors’ signal output. Bao introduced a porous structure in PDMS (Figure 8b), serving as a Young’s module switchable approach for contact materials.^102^ Besides, paper^103,104^ and wood,^105^ which are eco-friendly, are also utilized as triboelectrification materials. Mao et al. proposed a paper-based triboelectric device (Figure 8c), with a maximum power density of 53 W/m^2^. It can effectively convert mechanical energy from the action of turning book pages into electricity, serving as a document monitor.^106^ Luo et al. reported a wood-based triboelectric sensor for athletic big data collecting and it applied in table tennis.^105^

With the development of a human–machine interface, devices based on materials with high flexibility, gas permeability, and wearability have been reported. Yi et al. showed a stretchable device by using a conductive liquid as the induction electrode covered with rubber. It can stand 300% strain and be able to sense arm motions.^107^ Similarly, Pu et al. presented a hydrogel triboelectric sensor,^108^ which can stand up to 1160% strain and delivers an output of 35 mW/m^2^ (Figure 8d). Another factor for wearable applications that should be considered is breathability. It is important to adjust the thermal–moisture balance and achieve gas exchange between human skin and the environment, and low breathability could cause skin discomfort and even induce inflammation and itching.^109^ Dong et al. fabricated a triboelectric sensor by sandwiching a silver nanowire (Ag NW) between polylactic-*co*-glycolic acid (PLGA) and poly(vinyl alcohol) (PVA).^110^ With the micro-to-nano hierarchical porous structure, the device has a high specific surface area and numerous capillary channels for thermal–moisture transfer (Figure 8e and 8f). Besides, researchers also developed functional fibers to compose an inherent wearable triboelectric sensor. For instance, Yang et al. fabricated yarn-based stretchable triboelectric sensor arrays (Figure 8g), detecting hand motions, which allows real-time translation of signs into spoken words.^111^ Other fiber-based TENGs can be found in the references.^106,107^

#### 2.2.3 Force-Sensitive Devices

**Figure 9:**
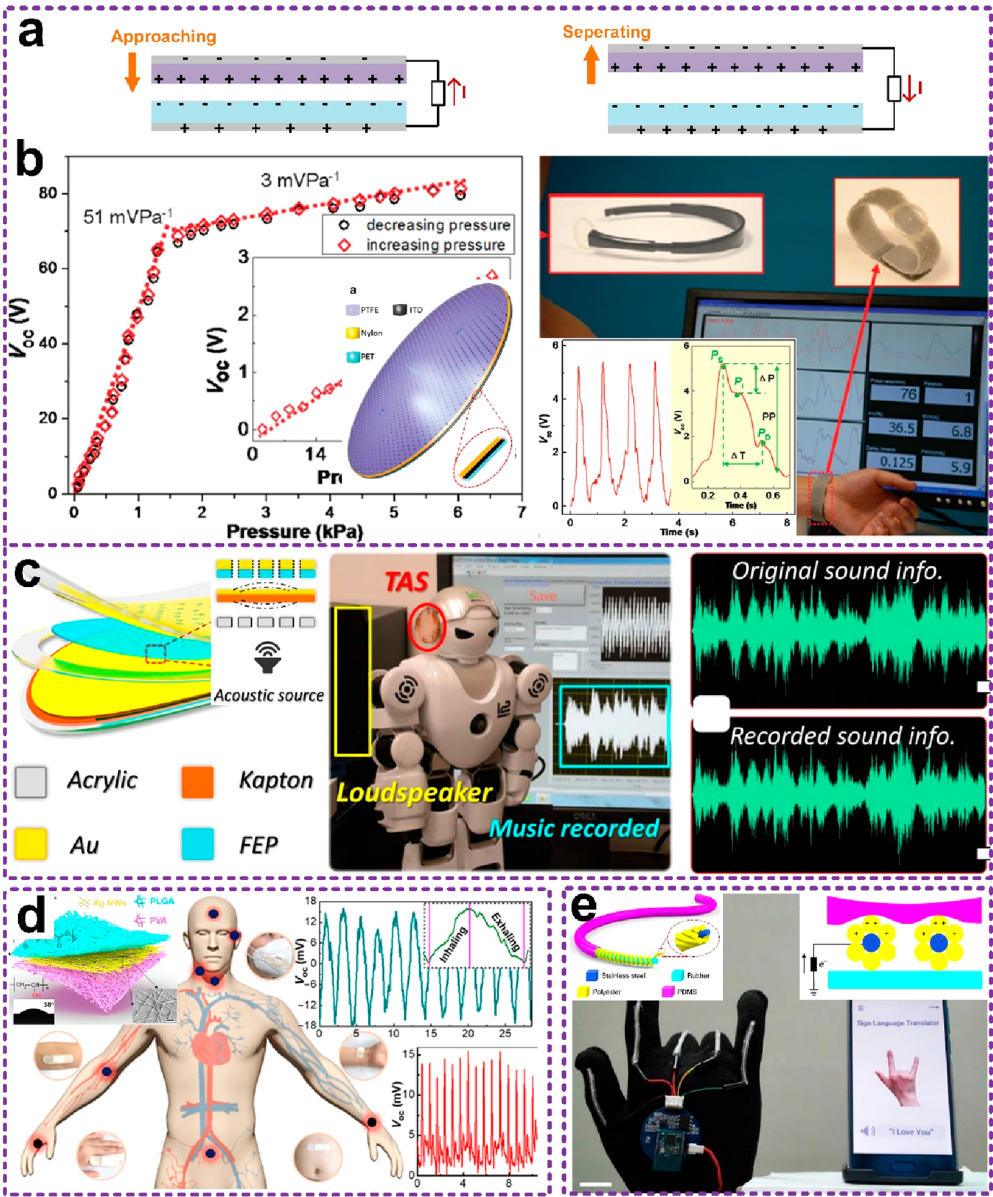
Force-sensitive triboelectric sensors. (a) Schematic of the general sensing mechanism. (b) Sphygmic monitoring. Reproduced with permission from ref (114). Copyright 2015 WILEY-VCH. (c) Sonic sensing. Reproduced with permission from ref (31). Copyright 2018 AAAS. (d) Human motion detecting. Reproduced with permission from ref (110). Copyright 2020 The Authors. (e) Gesture monitoring. Reproduced with permission from ref (111). Copyright 2020 The Authors.

Triboelectric sensors are inherent to deliver electricity output under external mechanical triggers. Thus, many self-powered wearable sensors have been proposed. Among them, devices based on force-sensitive mechanisms have been widely investigated. Figure 9a show a schematic of the general sensing mechanism. When the external mechanical trigger is applied, the gap between two contact materials varies and then induces an output potential. Figure 9b illustrates a soft pressure sensor, which is made up of PTFE/nylon with ITO as the induction electrode. The device works in the single-electrode mode. When an external mechanical force is triggered, PTFE approaches nylon, causing electron flow in the circuit, delivering a sensitivity of 51 mV/Pa with a response time less than 6 ms. Then, it was demonstrated to measure the dynamic pressure of human sphygmic or work as an anti-interference throat microphone, which could be used for recovering the human throat voice even in an extremely noisy environment.^114^Figure 9b illustrates a contact-separation mode TENG, working as an auditory sensor. It is based on a porous structure, with the Au/FEP as the electrification pair, and delivers a sensitivity of 110 mV/decibel. Then, it was used to construct a hearing aid, which simplified the signal processing and reduced the power consumption.^31^ In order to enhance the comfort and air permeability, researchers employed electrospinning to fabricate a skin-compliant strain sensor (Figure 9c). Its high specific surface area and numerous capillary channels assure the thermal–moisture transfer, which was found to be 120 mm/s, compared to commercial jeans of 10 mm/s.^110^Figure 9d illustrates a TENG strain sensor made up of fibers. It offers excellent mechanical durability, high sensitivity, and a quick response time and then was used to construct a wearable sign-to-speech translation system. A total of 660 sign language hand gestures based on American Sign Language (ASL) were acquired and successfully analyzed, with a high recognition rate of 98.63% and a short recognition time of less than 1 s.^112^

#### 2.2.4 Displacement-Sensitive Devices

**Figure 10:**
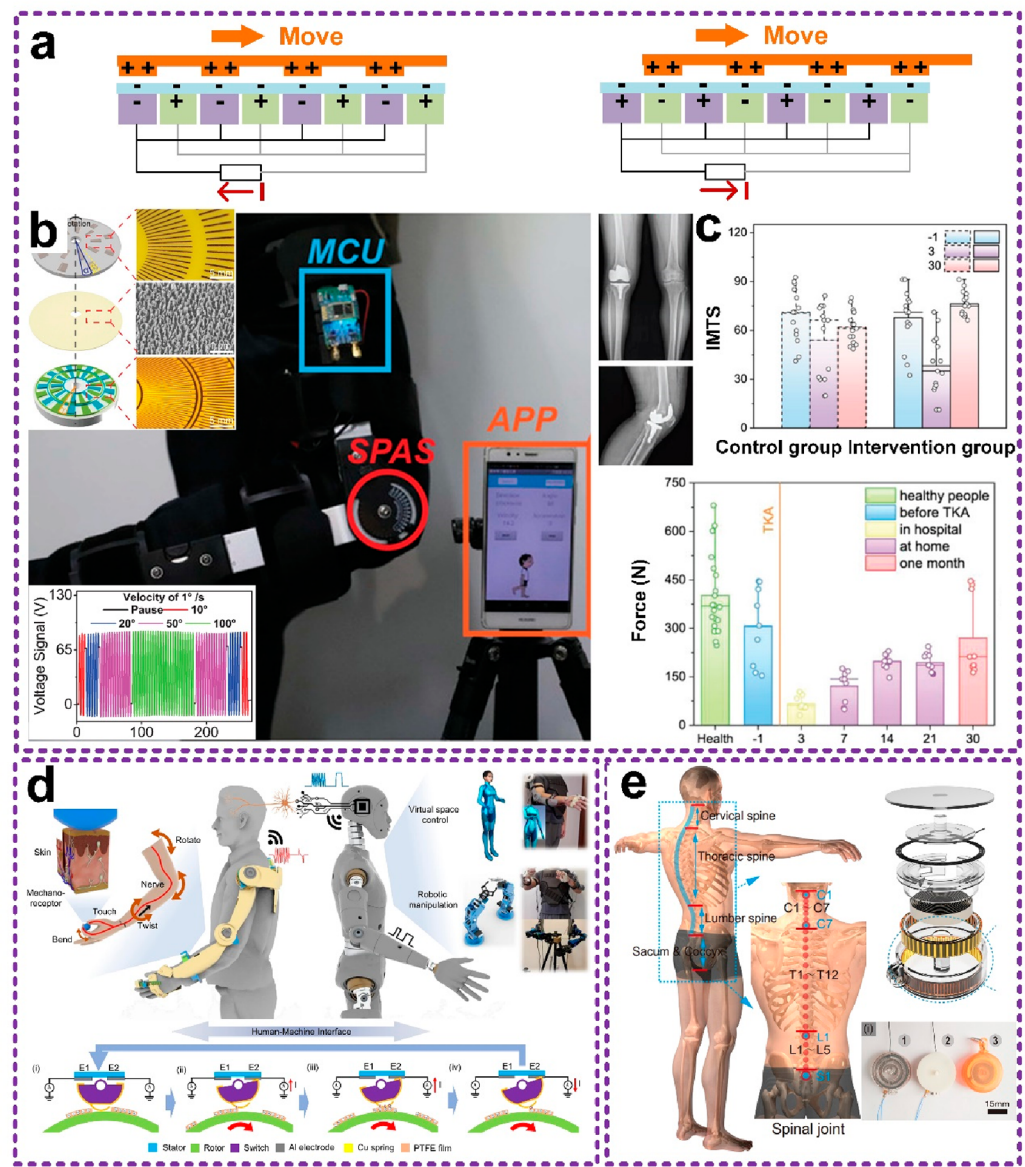
Displacement-sensitive sensors. (a) Schematic of the general sensing mechanism. (b) Angle sensor. Reproduced with permission from ref (115). Copyright 2020 WILEY-VCH. (c) Medical exoskeleton. Reproduced with permission from ref (116). Copyright 2020 The Authors. (d) Bidirectional rotation sensor and exoskeleton. Reproduced with permission from ref (117). Copyright 2021 The Authors. (e) Stretch sensor. Reproduced with permission from ref (118). Copyright 2021 The Authors.

A force-sensitive mechanism has been widely utilized for its shape compliance and simple structure. However, environmental influences, the materials’ viscoelasticity, and fatigability will inevitably affect the sensors’ output amplitude and thus lower the precision as well as the stability. To solve this problem, a displacement-sensitive mechanism was found to be a promising solution. It combines the relative sliding with the TENG’s grating electrodes, making the sensor output alternative waveforms, according to the displacement (Figure 10a). Thus, the signal’s phase variation indicates the information on the external mechanical motion. Even if the signal’s amplitude varies owing to influencing factors, its phase variation remains stable, assuring the sensing precision.

Figure 10b shows a TENG angular sensor.^115^ It consists of two rotation disks: one serves as the stator and the other as the rotator. Under mechanical triggers, the rotator rotates relative to the stator and continuously delivers periodic waveforms with one waveform corresponding to one electrode unit. According to the signal’s phase variation, the rotation degree can be obtained. When the temperature and humidity influence the signal amplitude, the phase will not change, so that the sensor possesses high precision. Subsequently, researchers demonstrated its applications in a medical rehabilitation exoskeleton, where the angular sensor was embedded and able to monitor the total knee arthroplasty (TKA) patients’ postoperative knee bending motions long term (Figure 10c). By combining data sharing and doctors’ guidance, patients achieved over 20–30% enhancement in recovery.^116^ Due to the inherent high precision of displacement-sensitive sensors, more sensing devices have been developed.^117−120^ For instance, Lee reported a bidirectional angular sensor, as shown in Figure 10d.^117^ It is embedded in an arm exoskeleton, serving as an economic and advanced human–machine interface for supporting the manipulation in both real and virtual worlds. Figure 10e presents a stretchable sensor based on a cyclic annular TENG encapsulated in a retractable reel.^118^ It was demonstrated to be able to monitor the spinal bending of the participant, delivering a displacement resolution of 0.6 mm, which corresponds well to the traditional inclinometer and deep camera. Furthermore, researchers also demonstrated its high durability even after 1 million stretching cycles due to the displacement-sensitive mechanism.^119^

Here, we provide a table to show the general differences between the two sensing mechanisms discussed above, Table 1.

**Table 1:** Comparisons of Two Different TENG Sensing Mechanisms

					characteristics		
sensing mechanism	TENG mode	fabrication	sensing objects	signal processing	positives	negatives	ref
force sensitive	contact separation, single electrode	material synthesis, stacking, 3D printing, surface microengineering	force, pressure, strain, stretching, angle	analog signal, amplitude measurement	high sensitivity, soft structure	external interference, low accuracy	(31, 110−114)
displacement sensitive	sliding, free standing	printed circuit board (PCB) process, surface microengineering	stretching, angle	discrete signal, phase measurement	high accuracy, batch fabrication	rigid supporting structure, less sensing objects	(115−120)

As we can see, displacement-sensitive sensors could be inert to the environmental factors and materials’ fatigue and then deliver accurate sensing performance. However, the present sensing objects are not diverse; devices with more functionalities, e.g. sensing force, pressure, etc., need be exploited based on this mechanism.

### 2.3 Piezotronics and Tribotronics

Piezotronic and tribotronic sensors are typical extensions of piezoelectric and triboelectric sensors, which utilize the piezo- or tribopotentials as the gate voltage to modulate various semiconductor devices (e.g., field effect transistors, memristors, Schottky diodes) and realize the sophisticated sensing functions.

#### 2.3.1 Piezotronic Mechanism and Typical Wearable Sensors

**Figure 11:**
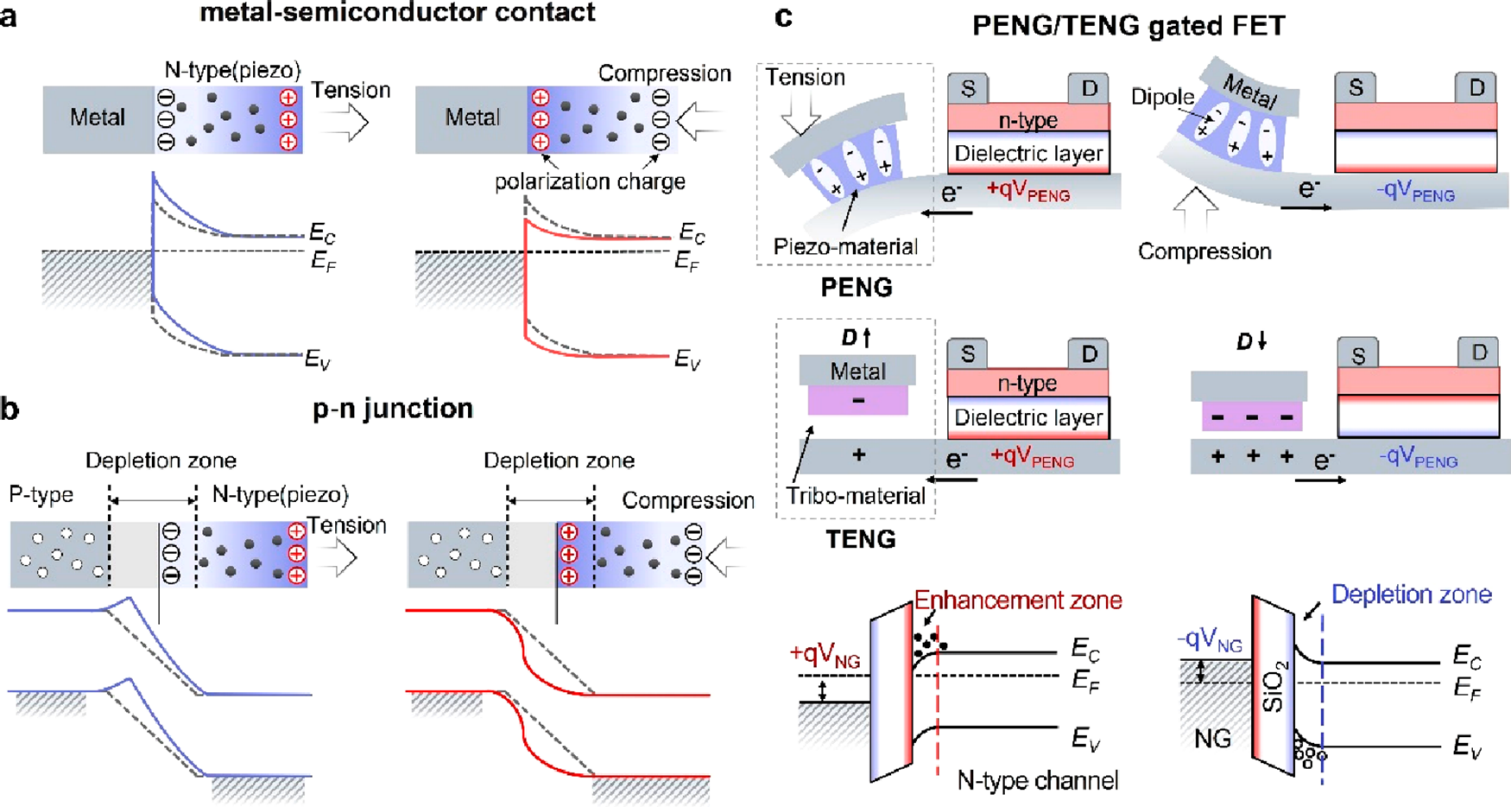
Piezotronic effect and piezo/triboelectric potential modulation. Schematic energy diagrams of the piezotronic effect at the metal–semiconductor contact (a) and p–n junction (b) under tensile and compressive strains. Reproduced with permission from ref (126). Copyright 2010 Springer Nature. (c) Piezoelectric and triboelectric potential modulation in FET.

Piezotronic devices (i.e., piezotronics) utilize the piezoelectric potential to specifically adjust the carrier transport properties at the metal–semiconductor contact interface (M–S contact) or p–n junctions through tuning the Schottky barrier height (SBH).^121−125^ As is known, the piezopotential is a locally induced electrical field in a noncentrosymmetric crystal by external strain, which originates from the nonannihilative and nonmobile ionic charges. As illustrated in Figure 11a and 11b, when a semiconductor device (in asymmetric structure) is subjected to an applied tensile strain, a negative piezopotential will be induced, repel electrons away from the p–n junction or M–S contact interface, and lead the local SBH to increase under the influence of negative polarization charges. On the contrary, externally applied compressive strain will induce a positive piezoelectric potential, attract electrons toward the M–S interface/p–n junction, and cause the local SBH to decrease under the influence of positive piezoelectric polarization charges.

In addition to the strain-gated two-terminal devices, piezotronics can be extended to a more general definition, e.g., the piezopotential/strain-gated transistors which can be functionalized under the piezopotential induced by externals strain. The rational design of PENG-gated three-terminal devices is exhibited in Figure 11b. The integrated PENG component is composed of the active piezoelectric material sandwiched between two electrodes. The induced piezopotential in a piezoelectric polymer is an intrinsic inner crystal field according to the enhanced/weakened electric dipole moments in the piezoelectric materials under external stress.^126^ The applied stress can lead to the rearrangement of electric dipoles along different directions. Applying tensile or compressive stress can induce a negative or positive gate voltage (−*qV*_PENG_ and +*qV*_PENG_) to the FET and lead to energy band bending and carrier depletion/accumulation in the semiconductor channel.

**Figure 12:**
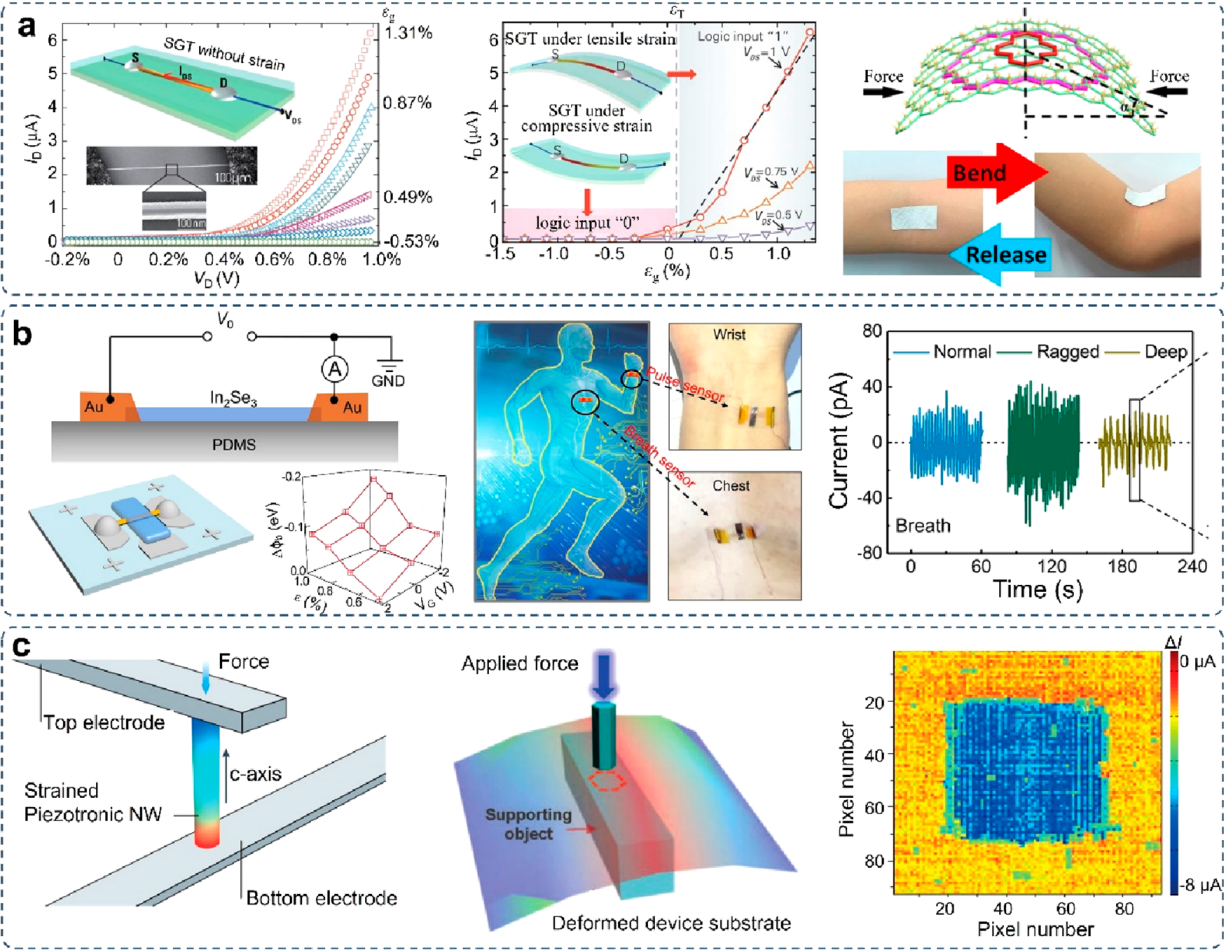
Piezotronic wearable sensors. (a) Piezotronic strain sensors. Reproduced with permission from ref (124). Copyright 2010 WILEY-VCH. (b) Self-powered piezoelectric sensors based on a multilayer α-In2Se_3_ flake for real-time monitoring of breath signals. Reproduced with permission from ref (130). Copyright 2019 American Chemical Society. (c) Array integration of vertical ZnO nanowire piezotronic transistors for tactile imaging. Reproduced with permission from ref (131). Copyright 2013 AAAS.

Based on the interfacial modulation of charge carriers, the piezotronic effect mainly describes the coupling effect between piezoelectricity and charge carrier transport in piezoelectric semiconductors.^123,124,127,128^ A typical flexible strain sensor relying on the piezotronic effect is exhibited in Figure 12a,^129^ which is composed of a horizontal single-crystal ZnO nanowire bonded to a plastic substrate. Its volt–ampere characteristics (*I*–*V* curves) indicate the strain sensor is highly sensitive due to the fact that the induced remnant piezoelectric charges can have a significant influence on the SBH and dramatically change the output current. Notably, the piezotronic effect should be distinguished from the piezoresistive effect (commonly existing in conventional semiconductor materials) according to the typical asymmetric effect on the two contacts, nonlinear and asymmetric rectifying *I*–*V* curve, strong polarity/interface effect/switch function, etc. The measured gauge factor for the ZnO piezotronic strain sensor, defined as the curve slope of the normalized current vs strain, is evaluated to be 1250 with a fast response time of 10 ms. The ZnO piezotronic strain sensor is also readily modulated by solid-state electrolyte to achieve tunable piezotronic strain sensors in a low-power-consuming way.^125^ In addition to 1D materials, the piezotronic effect has also been observed in 2D materials, e.g., single-layer MoS_2_, MoSe_2_, and multilayer α-In_2_Se_3_ flake.^130^ For instance, a piezotronic sensor based on an α-In_2_Se_3_ flake has been prepared for breath monitoring in a self-powered fashion (Figure 12b), which can effectively record three different breath states. The prepared In_2_Se_3_ piezotronic sensor can operate in an active and direct coupling means, exhibiting significant advantages in breath state detection according to the synchronization between breath frequency and output signals.

In addition to single piezotronic devices, the integration of multiple piezotronic nanodevices into an active matrix/array plays a critical role in realizing a functional system for high-resolution sensation. Wu et al. tried to demonstrate an integrated piezotronic transistor array with vertically aligned ZnO nanowires by combining the bottom-up and top-down microfabrication techniques. The piezotronic transistor array can be utilized as a taxel-addressable active matrix for imaging the external tactile information even by removing the gate electrodes (Figure 12c).^131^ The demonstrated piezotronic active matrix enables tactile pressure imaging in a self-powered means by only applying external mechanical stimuli. The simplified fabrication and integration process for the active matrix also offers a significant route to developing more diversified smart wearable sensors with multifunctionalities.

#### 2.3.2 Tribotronic Mechanism and Typical Wearable Sensors

**Figure 13:**
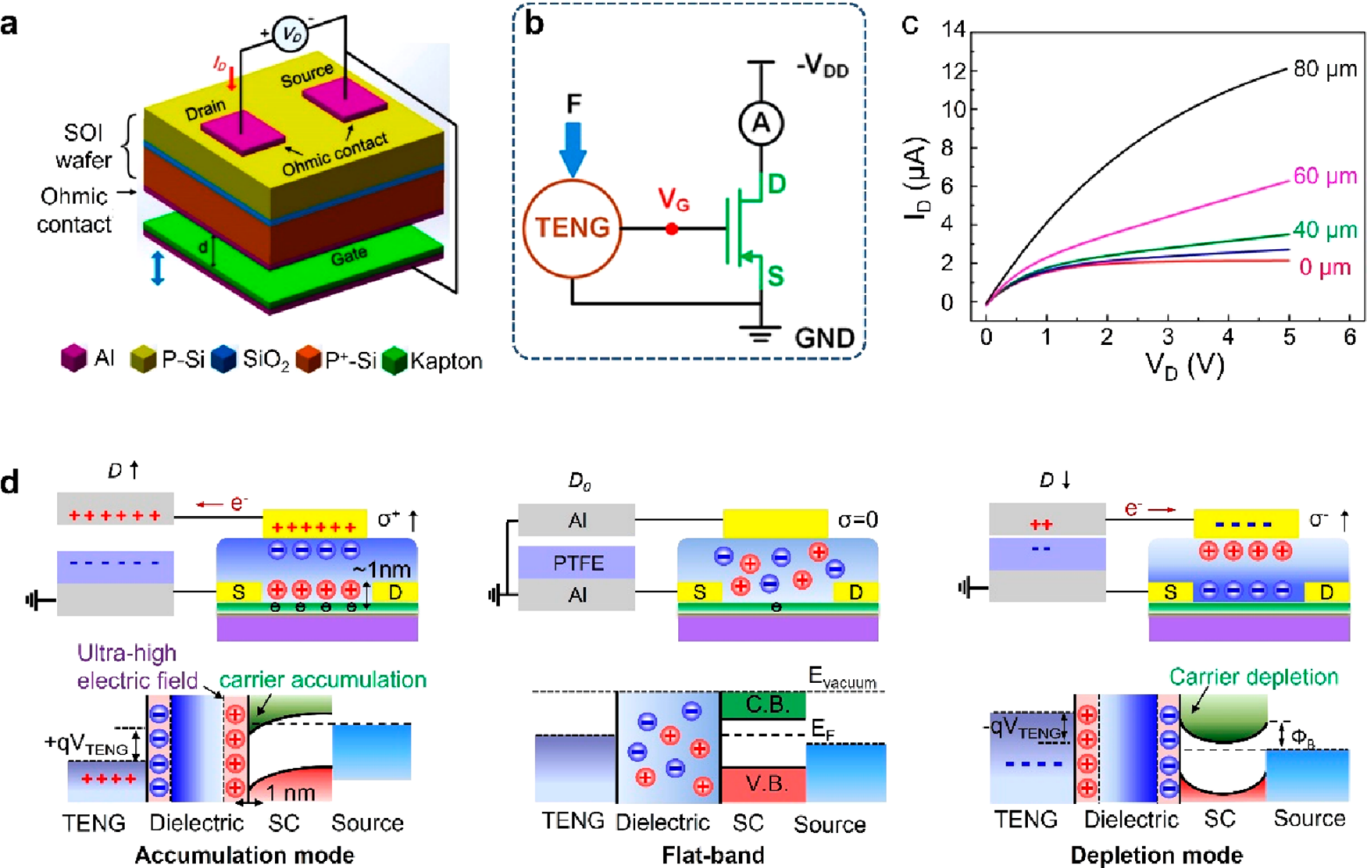
Tribotronic transistor and working mechanism. (a) Schematic illustration of a typical tribotronic transistor. Reproduced with permission from ref (133). Copyright 2014 American Chemical Society. (b) Equivalent circuit diagram of the tribotronic transistor. Reproduced with permission from ref (147). Copyright 2017 American Chemical Society. (c) Output characteristics of a tribotronic transistor under at different TENG displacements. Reproduced with permission from ref (133). Copyright 2014 American Chemical Society. (d) Working principle (top) and energy band (bottom) diagrams of the tribotronic transistor at three modes: accumulation mode, flat-band mode, and depletion mode. Reproduced with permission from ref (148). Copyright 2019 WILEY-VCH.

Similar to piezotronics, tribotronics^133−135^ is an emerging field utilizing the triboelectric potential to modulate the charge carrier transport in semiconductor devices instead of the applied gate voltage (bottom panel in Figure 11b). According to the material diversity and excellent functionality,^77,96,136^ tribotronic devices have been widely investigated in a variety of wearable sensing applications, including tribotronic logic gates,^137,138^ tactile controlled light emitting diodes (OLEDs),^139^ memory devices,^140^ smart tactile sensors,^141−143^ and wearable/flexible displacement sensors.^144,145^ Wang’s group has reported the first tribotronic device by combining a TENG with a metal–oxide–semiconductor FET (MOSFET), also known as contact electrification FET (CE-FET) (Figure 13a).^133^ The contact-separation-induced triboelectric potential can fully replace the external gate voltage and reduce corresponding energy consumption. An equivalent circuit diagram of the tribotronic transistor and the basic output characteristics under different vertical displacements (*D*) are presented in Figure 13b and 13c, respectively. Different from the traditional electrical behaviors of a transistor under sweeping gate voltage, the drain current (*I*_D_) of the CE-FET increases with increasing displacement *D* from 0 to 80 μm. The corresponding working mechanism and energy band diagram are shown in Figure 13d.^146^ At an initial state of *D* = *D*_0_, no charge transfer occurs between the integrated TENG and the transistor device. When *D* is controlled to increase or decrease, the induced positive/negative charges in the TENG are transferred to the gate and couple the positive/negative potential (σ+/σ−) to the transistor, resulting in the carrier accumulation/depletion and energy band bending in the semiconductor channel. In analogy with the important parameters of traditional transistors, e.g., the threshold voltage (*V*_th_) and subthreshold swing (*SS*), the tribotronic transistor can also be evaluated by analogous parameters, e.g., the tribotronic threshold value (*D*_t_) and the tribotronic subthreshold swing (*SS*_t_). *D*_t_ indicates the minimum TENG displacement required to establish a conductive path between the source and the drain electrodes. *SS*_t_, defined as *SS*_t_ = ∂(*D*)/∂(log 10(*I*_D_)), describes the minimum change of the TENG displacement (Δ*D*) required to contribute to 1 order of magnitude variation in *I*_D_.

**Figure 14:**
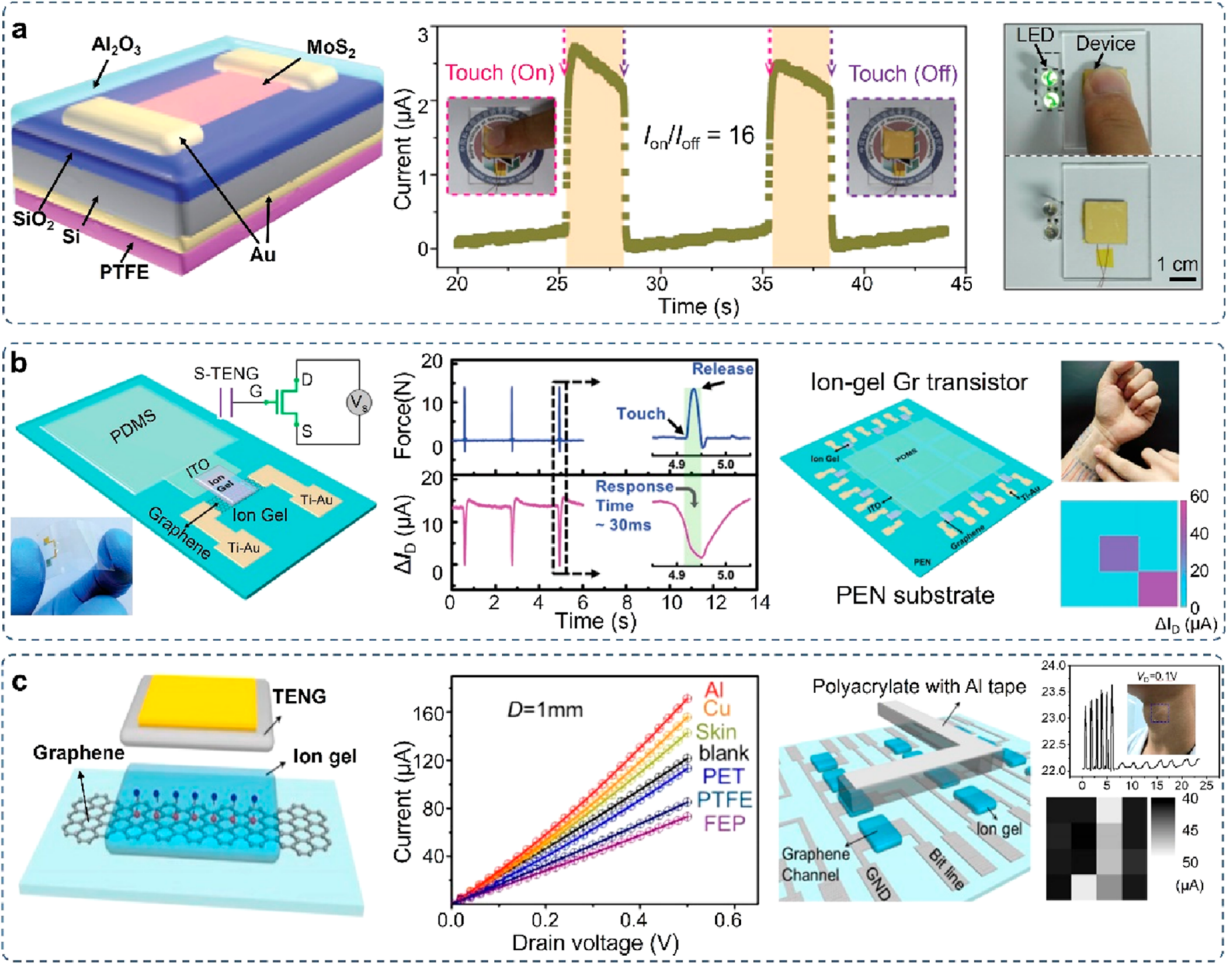
Tribotronic transistor for a smart touch sensor. (a) Tribotronic MoS_2_ transistor for tactile switch. Reproduced with permission from ref (150). Copyright 2016 WILEY-VCH. (b) Tribotronic graphene transistor for touch screen applications. Reproduced with permission from ref (151). Copyright 2016 WILEY-VCH. (c) Mechanosensation-active matrix based on a tribotronic coplanar graphene transistor array. Reproduced with permission from ref (144). Copyright 2018 American Chemical Society.

Tribotronic devices are potentially applicable for flexible and wearable touch sensors, human–machine interfaces, and artificial robotics. For instance, a tribotronic MoS_2_ transistor has been developed by integrating a MoS_2_ FET with a single-electrode mode TENG and applied as a smart tactile switch (Figure 14a).^141^ The triboelectric potential induced by the TENG displacement is available as the gate voltage to modulate the charge carrier transport in the MoS_2_ channel, achieving an on/off ratio of ∼16 to realize a direct tactile switch using fingers to light up double LEDs and indicate the tactile information. Notably, Wei et al. tried to demonstrate a high-performance tribotronic transistor array with record high current on/off ratios (>10^8^) by combing an integrated TENG with a large-area organic transistor array,^149^ which further offers an effective wearable interactive intelligent system, artificial robotic skin, and wearable mechano-driven electronic terminals.

Relying on ion-gel gating, a tribotronic graphene tactile sensor is prepared on a flexible substrate based on a single-electrode mode TENG and a coplanar gate graphene transistor (Figure 14b).^142^ The tribotronic transistor shows excellent performance in wearable tactile sensing and spatial mapping, including high sensitivity (2% kPa^–1^), a superior detection limit (1 kPa), a fast response time (30 ms), and excellent stability. In order to extend the functionality of material recognition and approaching sensation, a mechanosensation-active matrix is prepared based on a direct-contact tribotronic transistor array (Figure 14c).^144^ A typical ion gel is utilized as both the dielectric layer of the graphene transistor and the friction layer of the TENG component to realize the direct-contact sensing process with high sensitivity (0.16 mm^–1^), fast response time (15 ms), and excellent durability (>1000 cycles). When the tribotronic device contacts with different materials, different output sensing performance can be successfully characterized according to the triboelectric series. Accordingly, the direct-contact tribotronic graphene transistor can enable the wearable sensing of the contact distance and the identification of different friction materials, exhibiting the following advantages: (i) realizing noninvasive sensing based on triboelectrification and charge transfer, (ii) greatly simplifying the fabrication process of the tribotronic transistor, and (iii) effectively reducing the power consumption through electrical double-layer gating (i.e., triboiontronics). The demonstrated applications of the simplification and low-power consumption in triboiontronic transistors promise more opportunities for flexible multifunctional electronics, intelligent interactive sensing systems, and diversified neuromorphic applications.

#### 2.3.3 Advanced Artificial Synapse Applications

**Figure 15:**
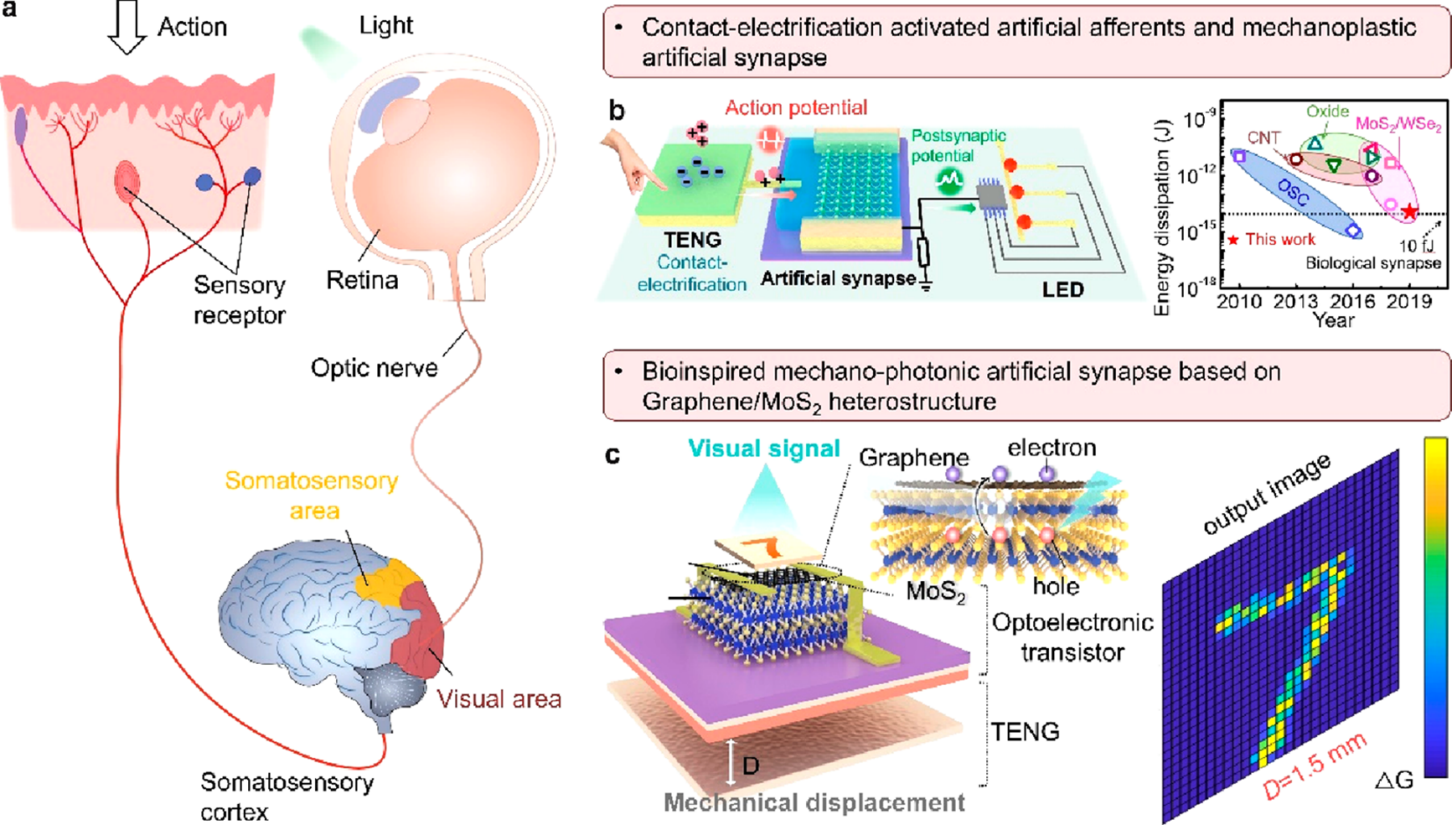
Tribotronics for an artificial synapse. (a) Schematic diagram of a biological tactile afferent nerve. (b) Bioinspired contact-electrification-activated artificial afferent. Reproduced with permission from ref (132). Copyright 2021 Springer Nature. (c) Bioinspired mechano–photonic artificial synapse. Reproduced with permission from ref (165). Copyright 2021 The Authors.

The integration of different types of sensors with synaptic devices in a synergistic fashion has pushed forward the development of interactive neuromorphic devices, which can not only sense/store/process external stimuli information in a direct means but also implement the biomimetic functions (e.g., perception, learning, memory, and even computation).^152^ The cooperation of receptors/neurons/synapses in the somatosensory system allows for effective recognition/processing on complex external tactile information.^153^ As shown in Figure 15a, the tactile stimuli signals are physiologically detected by mechanoreceptors on the skin and transmitted along the axons to postsynaptic neurons for further recognizing/processing and the tactile information.^154,155^ Generally, human skin is covered with different types of mechanoreceptors to record specific types of tactile stimuli and implement pressure/touch/tactile recognition. For instance, pressure receptors are commonly fast adaptive receptors made of Pacinian corpuscles to perceive pressure, while the touch receptors are usually slowly adaptive receptors made of Meisner bodies/Rufini bodies/Merkel discs to perceive touch information.^156^ Among the reported different types of mechanosensors, the resistive/capacitive mechanoreceptors can capture continuous static forces, while the piezoelectric/triboelectric mechanoreceptors can capture instantaneous dynamic pressures.^157^ Accordingly, to construct an interactive neuromorphic system, an artificial afferent nerve has been proposed to simulate the function of the human sensory system by integrating a TENG mechanoreceptor and an electrolyte-gated neuromorphic transistor (Figure 15b). Based on the working mechanism of the triboelectric–neuromorphic tactile system, the artificial afferent nerve can be activated by the induced triboelectric potential, utilized to monitor different types of stimulus information (e.g., mechanical displacement, tactile signal, lateral-sliding motion, and pressure), and conducted to identify the frequency/amplitude of external motions for simulating the behavior of a virtual stimulus in the cerebral cortex.^132^ Notably, the electrolyte-gated transistors reported in this work are generally classified into the electrostatically controlled electric double-layer transistors and electrochemical transistors according to whether the ions react with the semiconductor materials.^158^ The ion migration in the electrolyte can readily imitate the process and function of the neurotransmitters, which has been extensively investigated and contributed to the emerging neuromorphic devices.^159−162^ For instance, a versatile triboiontronic MoS_2_ transistor via a proton conductor has been demonstrated to utilize the triboelectric potential gating to modulate the transistor performance via proton migration/accumulation. It has been demonstrated as a mechanical behavior-controlled logic device and a neuromorphic sensory system, representing reliable and effective triboelectric potential modulation through protonic dielectrics.^163^

**Table 2:** Typical Self-Powered Sensors’ Performance and Characteristics

				characteristics		
devices	working principle	performance	active materials	positives	negatives	refs
strain sensor	piezoelectric	25 mV, under finger bending with a strain about 0.2%	ZnO nanowire	self-powered, simple structure	specific material	(62)
	triboelectric	1 V, under finger bending with a strain about 0.2%	PDMS/PE	high output, diverse material choices	external interference, specific structure	(111)
	piezotronic	0.6 μA@1 V, under a strain about 0.31%	ZnO nanowire	high integration	specific material	(130)
	tribotronic					
pressure sensor	piezoelectric	6 mV/Pa	PVDF with 3D structure	self-powered, simple structure	specific material	(66)
	triboelectric	51 mV/Pa	PTFE/Nylon	high output, diverse material choices, self-powered	external interference, specific structure	(114)
	piezotronic	0.5 μA/kPa@1 V	ZnO nanowire array	high integration	external power, specific material	(131)
	tribotronic	3.45 μA/kPa@0.5 V	PDMS/Al	high integration, high output	external power, specific structure	(151)

**Figure 16:**
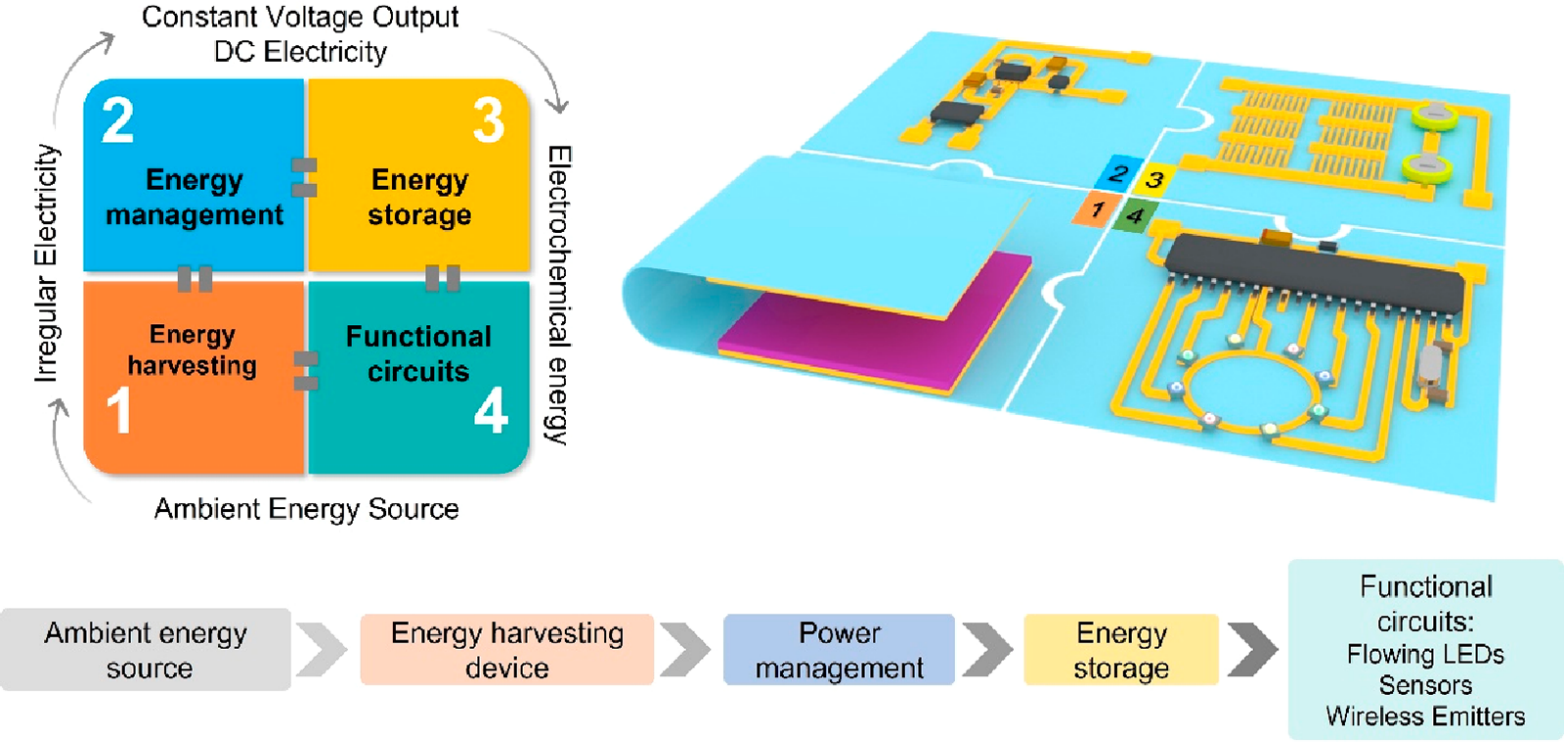
Schematic of self-powered sensing systems consisting of energy harvesting units, energy management units, energy storage units, and functional circuits (sensing units). Reproduced with permission from ref (167). Copyright 2022 The Royal Society of Chemistry.

**Figure 17:**
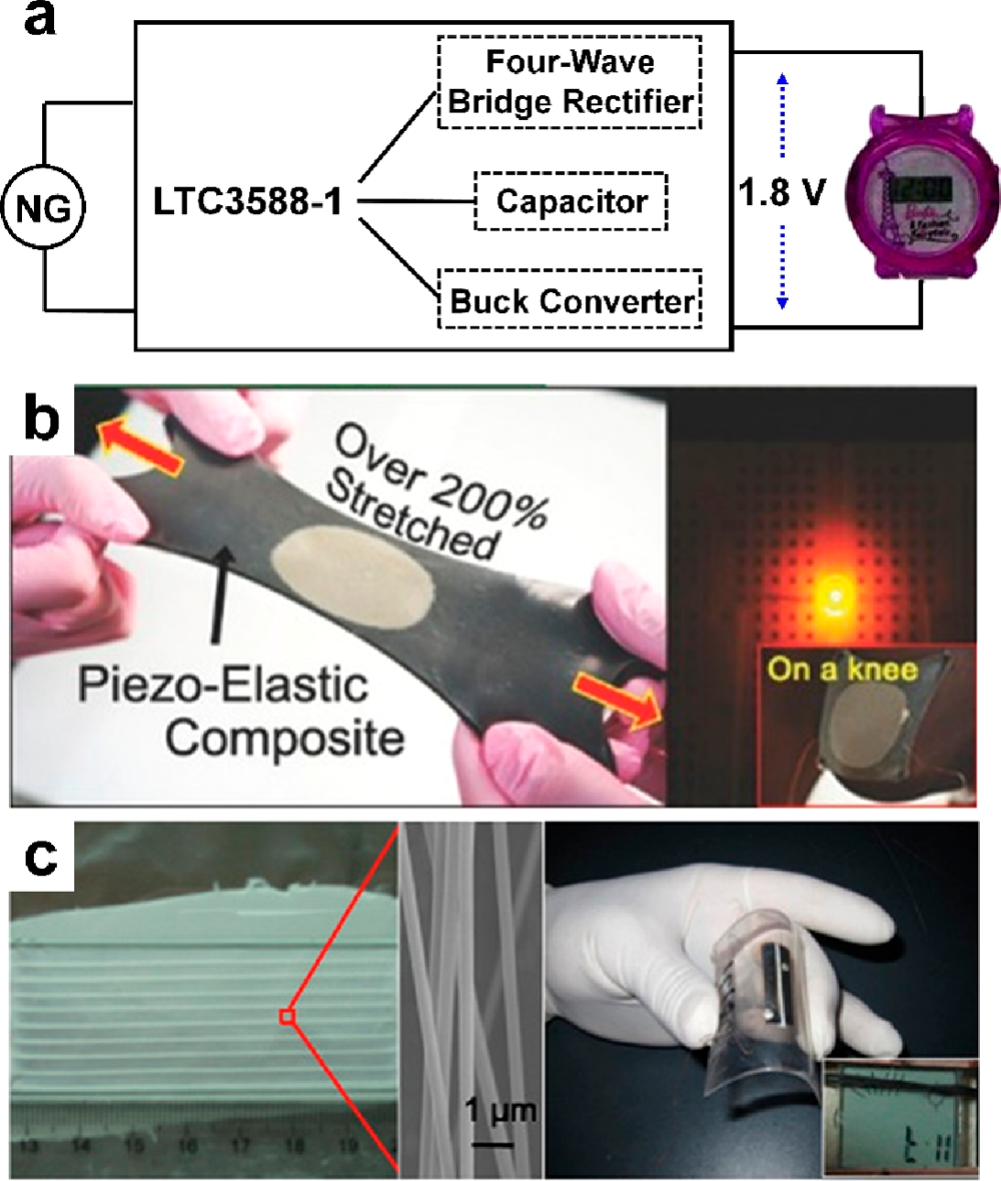
Piezoelectric nanogenerators as power sources. (a) Driving an electronic watch. Reproduced with permission from ref (27). Copyright 2011 WILEY-VCH. (b) Stretchable energy harvesting device. Reproduced with permission from ref (173). Copyright 2015 WILEY-VCH. (c) Driving a UV sensor. Reproduced with permission from ref (174). Copyright 2012 American Chemical Society.

**Figure 18:**
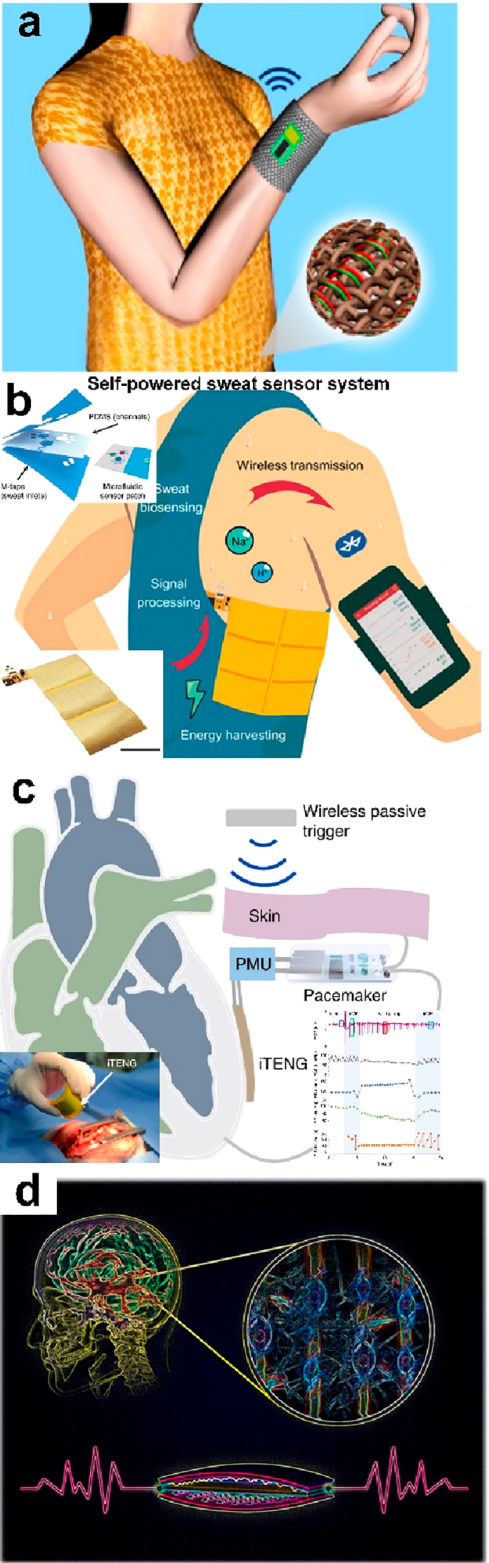
Self-powered systems employing TENGs as power sources. (a) Wireless temperature sensing system. Reproduced with permission from ref (176). Copyright 2014 American Chemical Society. (b) Wireless sweat sensing system. Reproduced with permission from ref (177). Copyright 2020 The Authors. (c) Symbiotic cardiac pacemaker. Reproduced with permission from ref (129). Copyright 2019 The Authors. (d) Electric stimulation system. Reproduced with permission from ref (190). Copyright 2016 American Chemical Society.

Besides, another mechanoplastic triboelectric neuromorphic tactile system has been constructed based on a floating gate FET (the terminology of “mechanoplastic” indicates the utilization of mechanical behavior to tune synaptic plasticity or update synaptic weight).^130^ The applied mechanical displacement can readily induce triboelectric potential to gate the transistor, trigger the PSC signal, and adjust the synaptic weight so as to realize the mechanical behavior modulated synaptic plasticity (i.e., mechanoplasticity). In this device, the system can implement both short-term and long-term plasticity according to the charge trapping in floating gate, realizing by mechanical displacement modulation in an active and interactive way. The triboelectric potential derived from TENGs can also be readily utilized to integrate with dual-gate transistor and implement multiple sensing applications. By integrating a triboelectric potential-powered dual-gate IGZO transistor with a common bottom gate and an air–dielectric top gate, a device-level versatile sensory platform is constructed to implement multifunctional sensations (including pressure/distance/optical sensors and artificial photonic synapse).^164^ Furthermore, Sun’s group^165^ has tried to introduce a bioinspired mechano–photonic artificial synapse with synergistic mechanical and optical plasticity (i.e., multimode/mixed-mode synaptic plasticity) **(**Figure 15c). Based on the integration of a graphene/MoS_2_ heterostructure-based phototransistor and an integrated TENG component, a mechanical displacement-tuned photoresponse is fulfilled based on the charge transfer/exchange in the heterostructure by the triboelectric potential. The reported mechano–photonic artificial synapses have provided an efficient route in implementing mixed mode interactions, simulating more complex biological neural systems and facilitating the development of interactive artificial intelligence.

At the end of this section, we selected two typical sensor devices, i.e., a strain sensor and a pressure sensor, and provide a comparative table to show the performance and characteristics based on various working principles, as shown in Table 2.

## 3 Self-Powered Wearable Systems

Unlike self-powered sensors, self-powered systems consist of functional circuits (sensor units), energy harvesting units, and energy management and storage units, as shown in Figure 16. The system’s power consumption is totally supplied by the energy harvesting units. In this section, we will review several of the main harvesting principles, including piezoelectric, triboelectric, thermoelectric, and photoelectric, biofuel cells, and hybrid generators. In addition, energy storage units are also discussed. At the end, we give a table summarizing the power levels of these devices.

### 3.1 Wearable Power Sources

#### 3.1.1 Piezoelectric Nanogenerator

The first nanogenerator is proposed on the basis of the piezoelectric principle and then widely used as a power source for some self-powered applications, including environmental monitoring systems^166−168^ and security systems.^169,170^ Meanwhile, self-powered wearable systems have been developed.^171,172^Figure 17a shows an electronic watch, for the first time, powered by a one-layer ZnO nanowire generator. Specifically, the nanogenerator delivers a voltage of 20 V and a current of 6 μA. After regulation by a LTC3588 power management chip, the device successfully drives a commercial electrical watch for more than 1 min under mechanical triggering for 1000 times.^27^Figure 17b illustrates a high-performance and hyper-stretchable elastic-composite piezoelectric nanogenerator by using Ag nanowire (Ag NW) stretchable electrodes.^173^ It delivers an output of 4 V and 500 nA and can convert biomechanical stretching energy to electricity. Figure 17c shows a wearable UV sensor powered by a PENG, where the generator is made up of dense lead zirconate titanate (PZT) parallel nanowires, fabricated via electrospinning. Its output voltage and current are about 6 V and 45 nA.^174^ Besides, You also presented a self-powered wearable system via embedding PVDF films into shoes.^175^

#### 3.1.2 Triboelectric Nanogenerator

Compared to a PENG, a TENG shows high output performance. Therefore, more wearable electronics driven by TENGs have been proposed. For instance, Zhong et al. reported a self-powered wearable temperature monitoring system.^176^ The TENG was fabricated by using commodity cotton threads, a polytetrafluoroethylene aqueous suspension, and carbon nanotubes. It can convert human motion/vibration energy into electricity with an average output power density of 0.1 μW/cm^2^. Then, it was demonstrated as an effective power shirt to drive a wireless body temperature sensor system (Figure 18a). Song et al. proposed a TENG by using a flexible printed circuit board (FPCB), which achieved a high power output of ∼416 mW/m^2^.^177^ And, it can be used in a battery-free sweat monitoring system with multiplexed biosensors and wirelessly transmit data through Bluetooth during on-body human trials (Figure 18b). Li reported a symbiotic cardiac pacemaker that is fully driven by the large animal’s cardiac pacing via a TENG (Figure 18c). The system consisted of a pacemaker, a power management circuit, and a TENG device, which delivers a voltage of up to 65 V and an energy generation of 0.495 μJ with the energy consumption of a traditional pacemaker of around 0.377 μJ. Figure 18d shows a self-powered electric stimulation system for neural differentiation. It is found that the neural differentiation is dramatically improved by the as-generated electric pulse via a TENG triggered by human normal walking (Figure 18d). Besides, various self-powered wearable electronics have been disclosed by employing a TENG as the power source.^76,112,178−184^ It is worth noting that the PENG/TENG possesses pulse outputs, and therefore, related power management circuits for voltage regulation and energy storage are required. Since the PENG’s output voltage is several to tens of volts, a LTC3588 commercial chip is normally utilized,^144^ whereas the TENG’s output voltage ranges from tens to hundreds of volts, so custom-designed power management circuits have been reported.^185−189^ In brief, a high-impedance circuit element is utilized to obtain the as-generated high-voltage electricity, and then, the energy will be converted into low-voltage electricity by switchable capacitor arrays^185^ or bulk circuits.^186^

#### 3.1.3 Thermoelectric Generator

**Figure 19:**
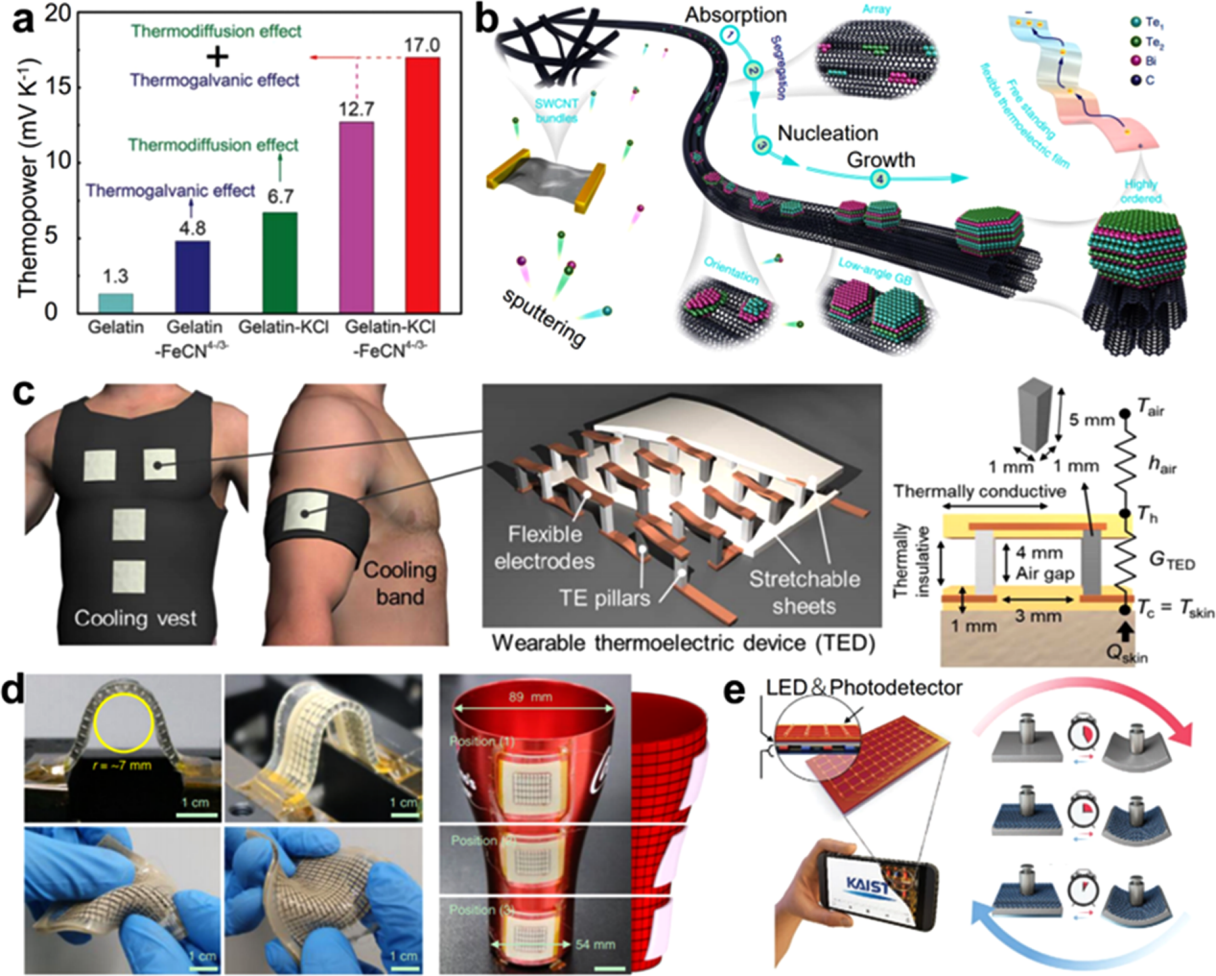
Thermal electric energy sources. (a) Ionic thermoelectric material using synergistic thermodiffusion and thermogalvanic effects. Reproduced with permission from ref (192). Copyright AAAS. (b) Illustration of the fabrication and structure of a free-standing highly ordered Bi_2_Te_3_–SWCNT hybrid thermoelectric material. Reproduced with permission from ref (191). Copyright 2018 Spring Nature. (c) Schematic illustration of cooling garments with wearable TEDs. Internal structure of the wearable TED with TE pillars connected by flexible copper electrodes and sandwiched between two stretchable sheets (right). Reproduced with permission from ref (193). Copyright AAAS. (d) Photographs of the compliant TEGs showing excellent conformability under various deformations. Scale bars 1 cm. Reproduced with permission from ref (194). Copyright Spring Nature. (e) Schematic illustration of the key design concept of the TES for rapid bidirectional conversion between a rigid hand-held electronic device and a soft wearable sensor. Reproduced with permission from ref (195). Copyright 2021 WILEY-VCH.

Generally, thermoelectric devices (TEDs) can realize the conversion of heat energy into electricity through the Seebeck/Soret effect. When there is a temperature gradient between two electrically connected conductors/semiconductors, the diffusion of charge carriers/ions can be induced to move away from the hot side which leads to a thermopotential and a consequent direct current (dc) flowing through the external circuit. Commonly, the maximum efficiency of the energy conversion process for power generation at a given temperature point is determined by the thermoelectric materials figure of merit *ZT*, given by *ZT* = σ*S*2*T*/κ, where σ is the material’s electrical conductivity, κ is thermal conductivity, and *S* is the Seebeck coefficient, which changes with temperature *T*. General state-of-the-art thermoelectric materials have *ZT* values of 2–3 with power densities reaching several tens of microwatts per square centimeter. For instance, Jin et al. reported a flexible thermoelectric material composed of Bi_2_Te_3_ nanocrystals in highly ordered crystalline alignment anchored on a carbon nanotube network (Figure 19a) The achieved maximum thermoelectric figure of merit (*ZT*) was evaluated to be ∼0.89 at room temperature due to the strong phonon scattering effect.^191^ According to the synergistic thermodiffusion and thermogalvanic effects, Han et al. demonstrated a giant positive thermopower (∼17.0 mV/K) in a flexible ionic thermoelectric material (Figure 19b).^192^ Hong et al. demonstrated a wearable TED with a high coefficient of performance (COP > 1.5), which can deliver more than a 10 °C cooling effect. The reported wearable TED with high flexibility is available to achieve long-term active cooling according to the novel design, which may inspire sophisticated personalized cooling with lower power consumption and improved comfort (Figure 19c).^193^ Lee et al. reported a compliant TED with stretchable interconnects and flexible conductors (silver-nanowire-based soft interconnects and metal particle magnetically self-assembled conductors) to realize high thermoelectric performance combined with excellent conformability (Figure 19d).^194^ Byun et al. prepared a flexible TED constructed on a gallium platform (Figure 19e), which can realize active temperature control to advance the solid–liquid phase transition of gallium based on the compact design and fast mechanical mode switching function. The flexible TED system provides new chances for personalized electronics, artificial robotics, and intelligent biomedical devices.^195^

#### 3.1.4 Photovoltaic Cell

**Figure 20:**
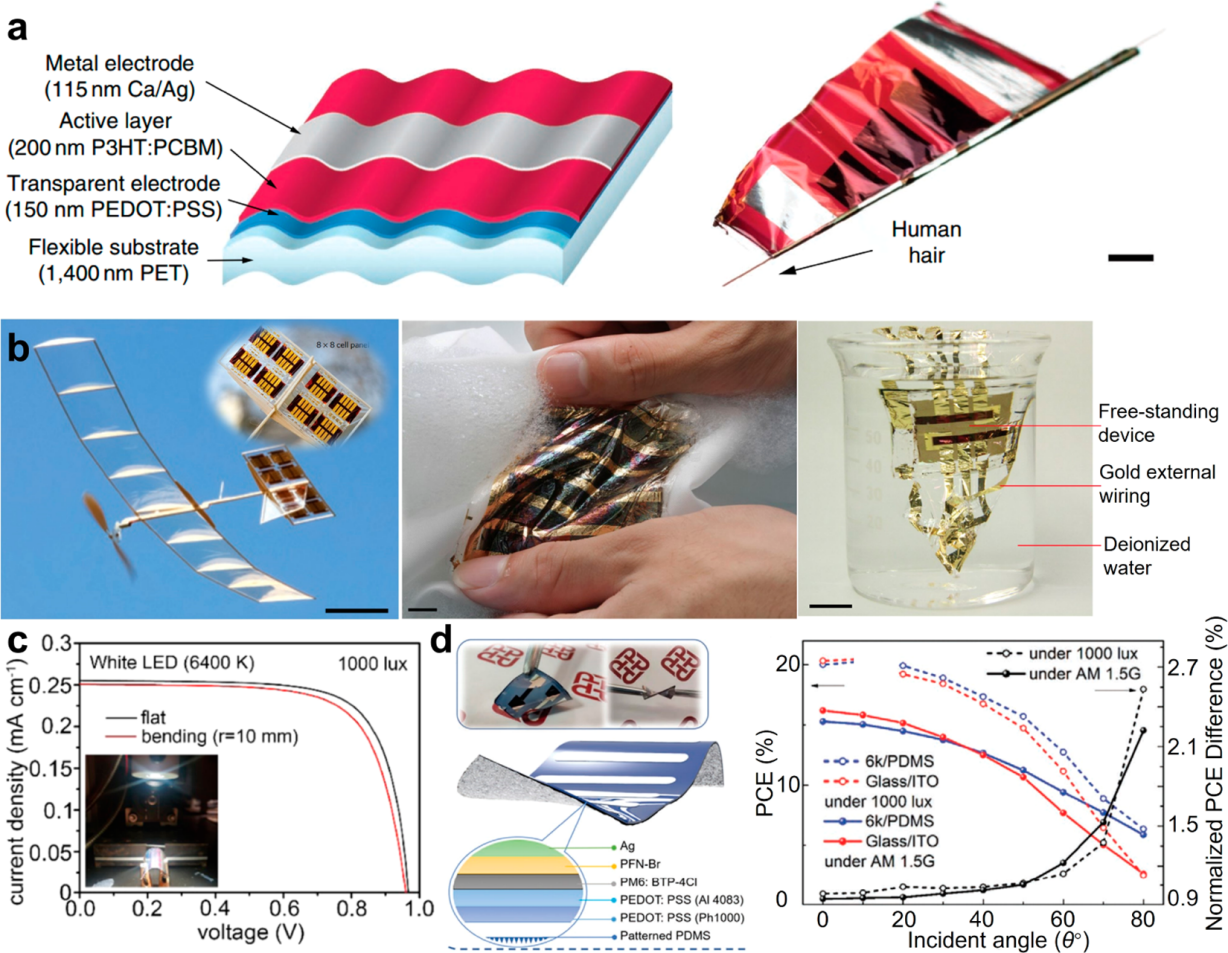
Photovoltaic cell. (a) Schematic of the ultralight and flexible organic solar cell. Layer thicknesses are drawn to scale. Extreme bending flexibility demonstrated by wrapping a solar cell around a 35 μm radius human hair. Scale bar 2 mm. Reproduced with permission from ref (199). Copyright 2012 Springer Nature. (b) Snapshot of the model plane during solar-powered outdoor flight. Scale bar 10 cm. Close-up photograph of the horizontal stabilizer with an integrated solar panel. Scale bar 2 cm. Photograph of the washing process for the devices conforming to a dress shirt. Scale bar 1 cm. Photograph of the dipping process. OPVs are submerged in deionized water. Scale bar 1 cm. Reproduced with permission from ref (196). Copyright Springer Nature. (c) *J*–*V* curves of the flat and bent devices (*r* = 10 mm) measured under illumination with a white LED (1000 lx). Reproduced with permission from ref (202). Copyright 2020 American Chemical Society. (d) Schematic diagram for surface-textured PDMS substrate fabrication and corresponding device structure. PCE/PCE enhancement of devices based on glass/ITO and 6k-PEDOT:PSS under AM 1.5G and 1000 lx of LED 2700 K. Reproduced with permission from ref (203). Copyright 2021 WILEY-VCH.

Solar cells, generally containing active layers, carrier-selective layers, and electrodes, can readily convert photonic energy into electrical energy based on the photovoltaic effects. Generally, the incident light is absorbed by the active layers and induces the generation of electron–hole pairs/excitons, which can be separated by the built-in potential and collected by the electrodes to produce an output current. As the thickness of the active layers in a solar cell may range from a few hundred nanometers to a few micrometers, how to reduce the thickness of the flexible substrate is of significant importance to increase the device flexibility/power density and reduce its weight (Figure 20a and 20b).^196−199^ Until now, the power conversion efficiencies (PCEs) of flexible organic solar cells and single-junction flexible perovskite solar cells under AM 1.5 G standard conditions have reached 16.61% and 21.73%, respectively.^200,201^ The PCE of flexible perovskite solar cells can be further increased to 23.33% at 400 lx and 28.63% at 5000 lx under the exposure of a weak light condition by a white light-emitting diode (Figure 20c).^202^ The PCT of a flexible organic solar cell has also been reported with an improvement to 20.5% under an indoor light illumination of 1500 lx (Figure 20d).^203^

#### 3.1.5 Biofuel Cell

**Figure 21:**
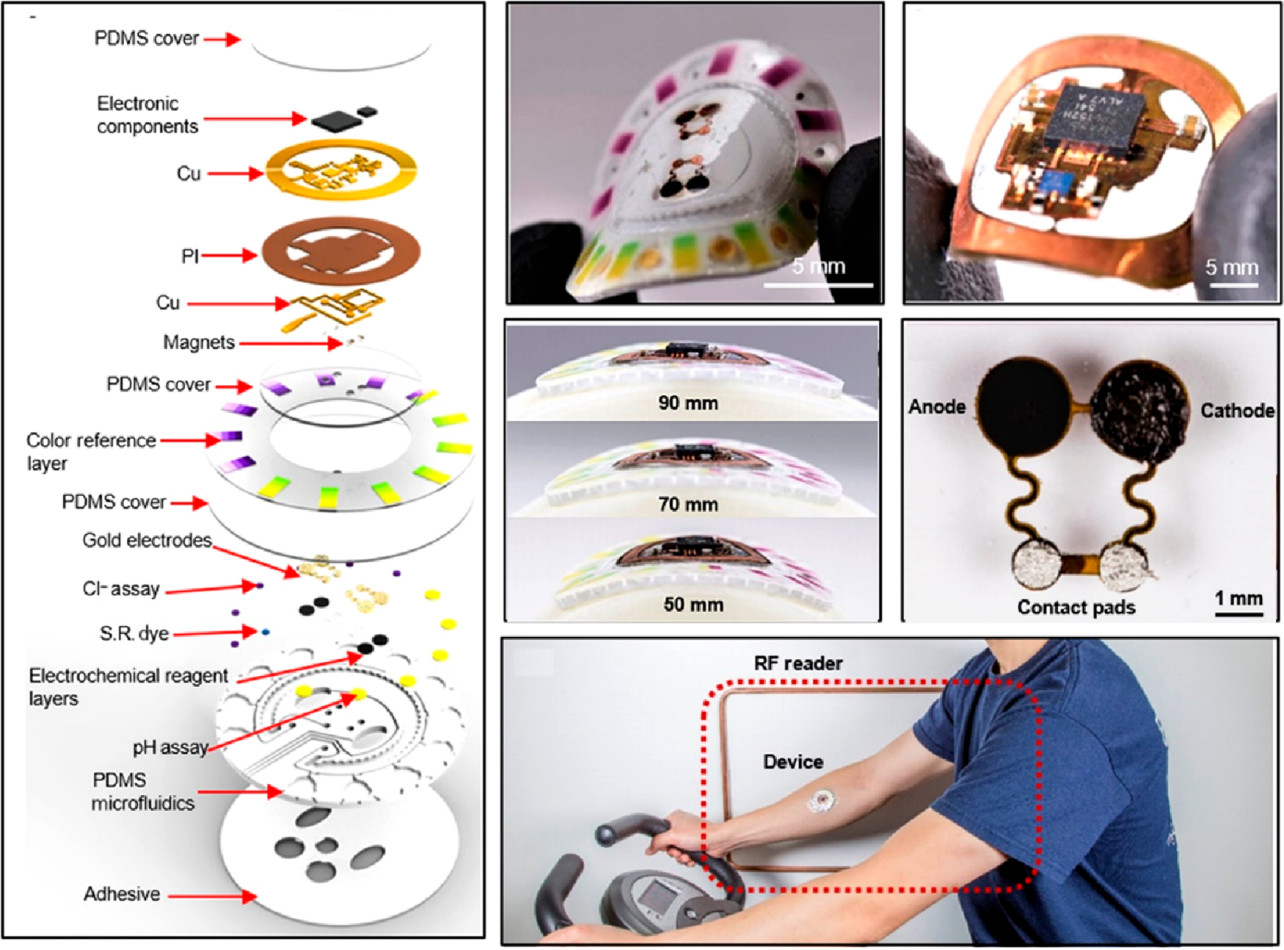
Battery-free, skin-interfaced microfluidic/electronic systems for simultaneous electrochemical, colorimetric, and volumetric analysis of sweat. Reproduced with permission from ref (205). Copyright 2019 AAAS.

Biofuel cells can readily convert biochemical energy into electricity, which generally relies on the redox reactions of various biofluids. Biofuel cells are commonly based on oxidizing biocatalysts, which can promote an oxidation reaction of fuels at the bioanode and a reduction reaction to water at the biocathode. The induced electrons flowing through an external electric circuit can help to generate output current and output power (Figure 21). The output current is determined by the concentration of the biofluids and the efficacy of the electron transfer process between the biocatalyst and the electrode. The power density is also improved to 3.5 mW/cm^2^ in the first developed milliwatt-level flexible sweat biofuel cell.^33,204−206^

#### 3.1.6 Hybrid Cell

**Figure 22:**
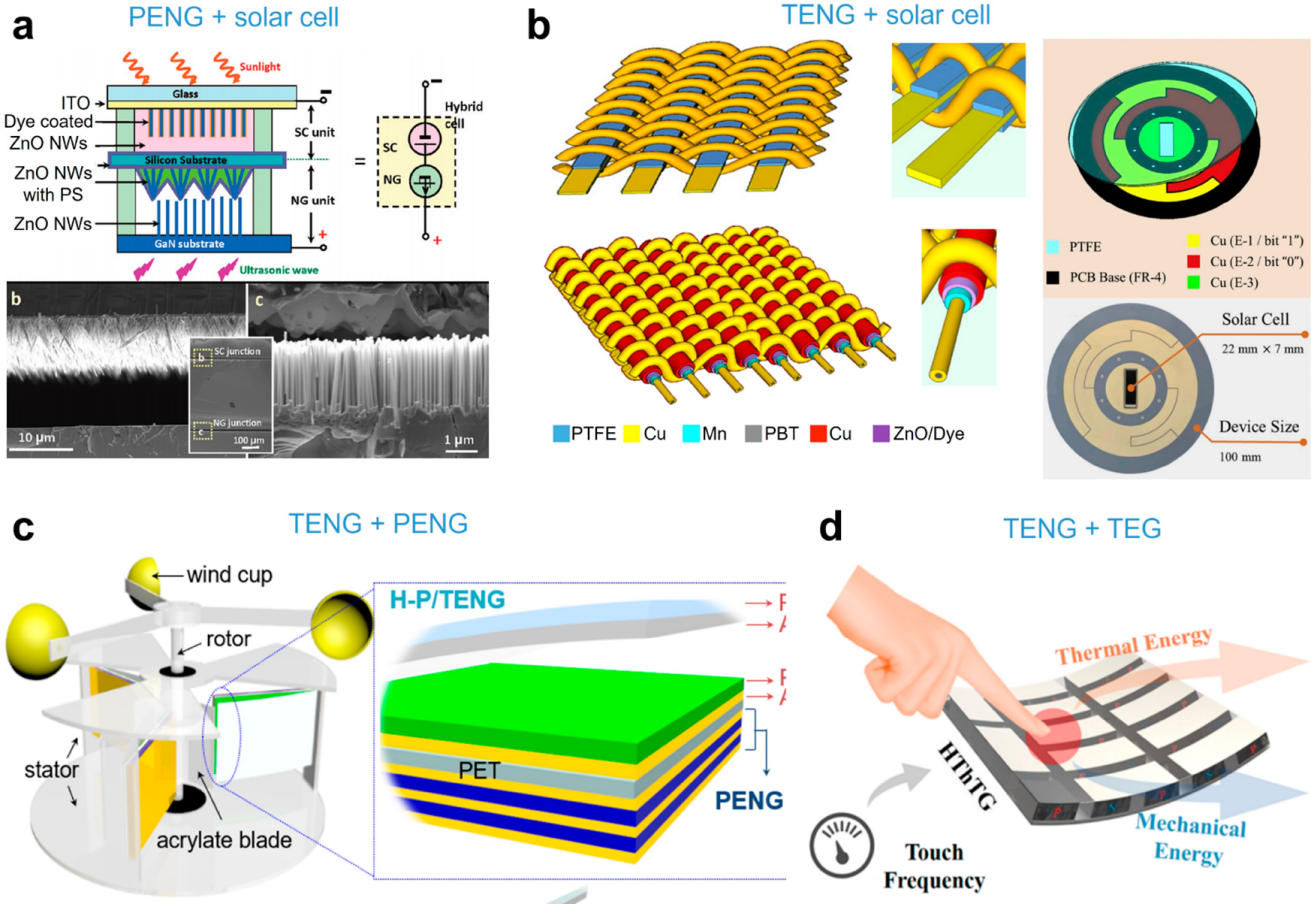
Hybrid energy harvesting for flexible wearable sensing. (a) Design and structure of a hybrid cell (HC) composed of a serially integrated solar cell (SC) and nanogenerator (NG) for raising the output voltage. Reproduced with permission from ref (207). Copyright 2009 American Chemical Society. (b) Schematic illustration of the hybrid power textile, which is a mixture of two textile-based all-solid energy harvesters: fabric TENG and photovoltaic textile. Reproduced with permission from ref (30). Copyright 2016 Springer Nature. (c) Schematic illustration of the H–P/TENGs mounted in the custom frame. (Inset) Enlarged structure of a single H–P/TENG. Reproduced with permission from ref (208). Copyright 2018 Elsevier Ltd. (d) Schematic representation of the working principle of a hybrid thermotriboelectric generator (HThTG). Reproduced with permission from ref (209). Copyright 2019 American Chemical Society.

As various and different forms of ambient energy are available as the energy sources in their related working environment, an effective strategy has been proposed to enhance the power density and sustainability by utilizing hybrid energy harvesters to power mobile/portable electronic devices. The first hybrid energy harvester was reported in 2009, combining a solar cell and a PENG to harvest both photonic and ultrasonic energy^207^ (Figure 22a). Other hybrid energy harvesters or energy harvesting strategies range from integrated TENGs and biofuel cells, integrated TENGs and solar cells, hybridized TENGs/PENGs (Figure 22b and 22c), and hybridized TENGs/TEDs (Figure 22d).^30,208,209^ The proposed integration of different/multiple energy harvesting devices on the same platform can be used to harvest two or multiple kinds of energy simultaneously, which is highly demanded to compensate for the intermittent drawbacks from one single energy source and significantly enhance the output power.

### 3.2 Wearable Energy Storage Units

Since the power generation is discontinuous or sometimes higher than the power consumption, power storage is required. In this section, we briefly show two typical wearable power storage technologies, i.e., supercapacitors and batteries.

#### 3.2.1 Supercapacitors

**Figure 23:**
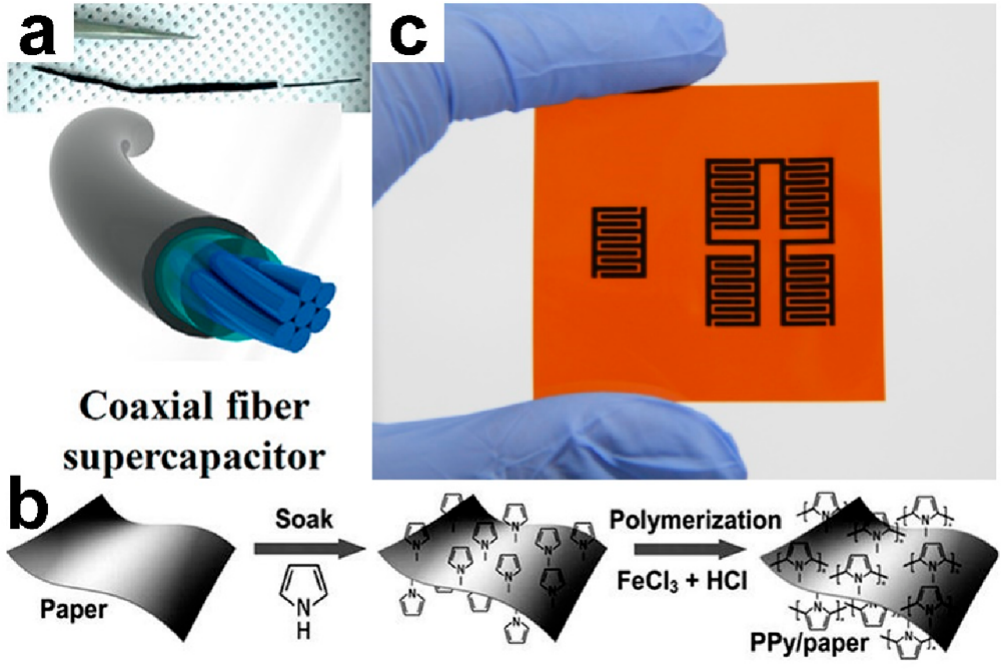
Wearable supercapacitors. (a) Fiber-based supercapacitor. Reproduced with permission from ref (212). Copyright 2013 American Chemical Society. (b) Film-based supercapacitor. Reproduced with permission from ref (213). Copyright 2014 The Authors. (c) Laser-direct-writing carbon-based supercapacitor. Reproduced with permission from ref (123). Copyright 2015 Tsinghua University Press and Springer-VBH.

Basically, a supercapacitor consists of two electrodes with current collectors, an electrolyte and a separator.^210,211^ Researchers have devoted great efforts on developing soft materials, high-density structures, as well as a facile fabrication process. Figure 23a shows a coaxial fiber supercapacitor consisting of carbon microfibers coated with carbon nanotubes. The capacitance reached 6.3 mF/cm.^212^Figure 23b presents a paper-based supercapacitor based on PPy soak and polymerization processing. It exhibits a capacitance of 0.42 F/cm^2^ with a high energy density of 1 mWh/cm^3^ at a power density of 0.27 W/cm^3^.^213^ Cui presented a cotton-fabric-based wearable supercapacitor with carbon nanotubes coated on, leading to an electrically conductive interconnecting network. Aqueous lithium sulfate is employed as the electrolyte. The device shows a high specific capacitance (∼70–80 F/g at 0.1 A/g) and cycling stability (negligible decay after 35 000 cycles).^214^Figure 23c shows an example of a facile fabrication. Luo et al. utilized direct laser writing to modify the surface of the polyimide. The as-generated graphene serves as the electrode of the capacitor. Then, the electrolyte and PDMS were successively deposited.^123^ More research on wearable supercapacitors can be found in the literature.^147,215−217^

#### 3.2.2 Lithium Batteries

**Figure 24:**
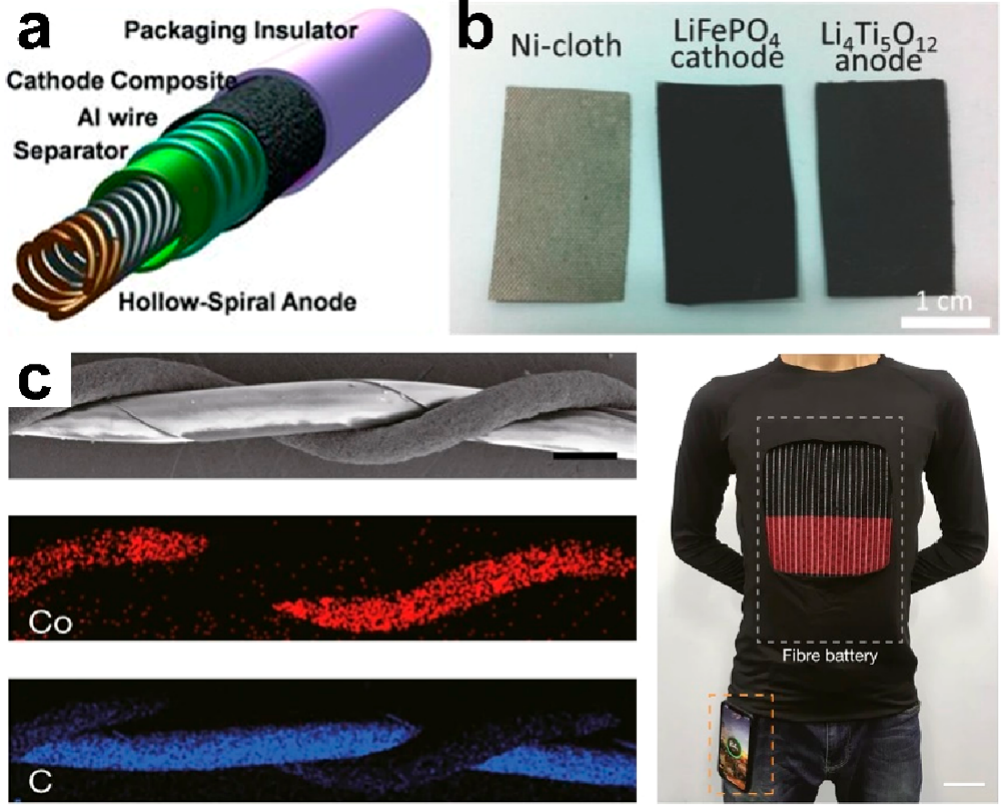
Wearable Li-ion batteries. (a) Fiber-based Li-ion battery. Reproduced with permission from ref (220). Copyright 2012 WILEY-VCH. (b) Textile-based Li-ion battery. Reproduced with permission from ref (221). Copyright 2015 WILEY-VCH. (c) Large-scale LIB clothes. Reproduced with permission from ref (222). Copyright 2021 The Authors.

For now, lithium batteries are indispensable for almost all kinds of electronics, especially Li-ion batteries (LIB). Hence, wearable battery technologies have been continuously developing.^218,219^ For instance, a cable-type battery with hollow-spiral, multiple-helix electrodes has been fabricated,^220^ showing a softening method for portable and wearable LIBs (Figure 24a). Textile-shaped batteries have also been presented,^221^ as shown in Figure 24b, where the conductive Ni-coated polyester textile is utilized as the current collector with coated active slurries of LiFePO_4_ and Li_4_Ti_5_O_12_, with a Celgard separator between. After 30 times folding, the LIB exhibits 84.5% capacity retention. In recent research, He et al.^222^ showed a landmark in wearable LIBs (Figure 24c). They produced meters of fiber LIB via a scalable process. Additionally, the fiber LIB shows an energy density of 85.69 Wh/kg and maintains over 80% capacity after being bent for 100 000 cycles. More research on wearable LIBs can be found in the literature.^193,223−226^

We give a summarized table to show the output levels of various energy harvesting devices as well as the energy density of the storage devices in Table 3.

**Table 3:** Output Performance of Energy Harvesting Devices and Storage Devices Commonly Used in Wearables^227^

category	devices	energy source	mechanism	output/capacity
wearable power sources	PENG	mechanical energy: vibration, wind, body motion	piezoelectric effect	ac, μW to a few mW cm^–2^ (peak), μW to a few mW g^–2^ (peak), up to 100 V
	TENG	mechanical energy: vibration, ocean wave, body motion	triboelectrification and electrostatic induction	ac, μW to mW cm^–2^ (peak), μW to mW g^–1^ (peak), p to kV (peak)
	TEG	heat: body, instrument, facility, the sun	ZnO nanowire	dc, ∼μW cm^–2^ ≈ mV K^–1^
	PV	light: the sun, indoor/outdoor light	photovoltaic effect	dc, a few tens of mW cm^–2^, a few tens of W g^–1^ (at a light intensity of 100 mW cm^–2^), ∼1 V
	biofuel cell	electrochemical energy: body fluid, sweat, blood	electrochemical reaction	dc, a few mW cm^–2^), ∼1 V
	hybrid cell	mechanical/optical; mechanical/thermal; mechanical/humidity	hybrid working mechanism	hybrid ac/dc; μW to a few tens of mW cm^–2^
wearable energy storage devices/systems	supercapacitors	chemical redox energy: electricity or chemicals	electric double-layer capacitance or pseudocapacitance	dc, up to 10 000 mW g^–1^ 1–4 V
	batteries	chemical redox energy: electricity or chemicals	electrochemical redox reactions	dc, hundreds of mW g^–1^, 2–5 V

## 4 Other Power Technologies for Wearable Systems

**Figure 25:**
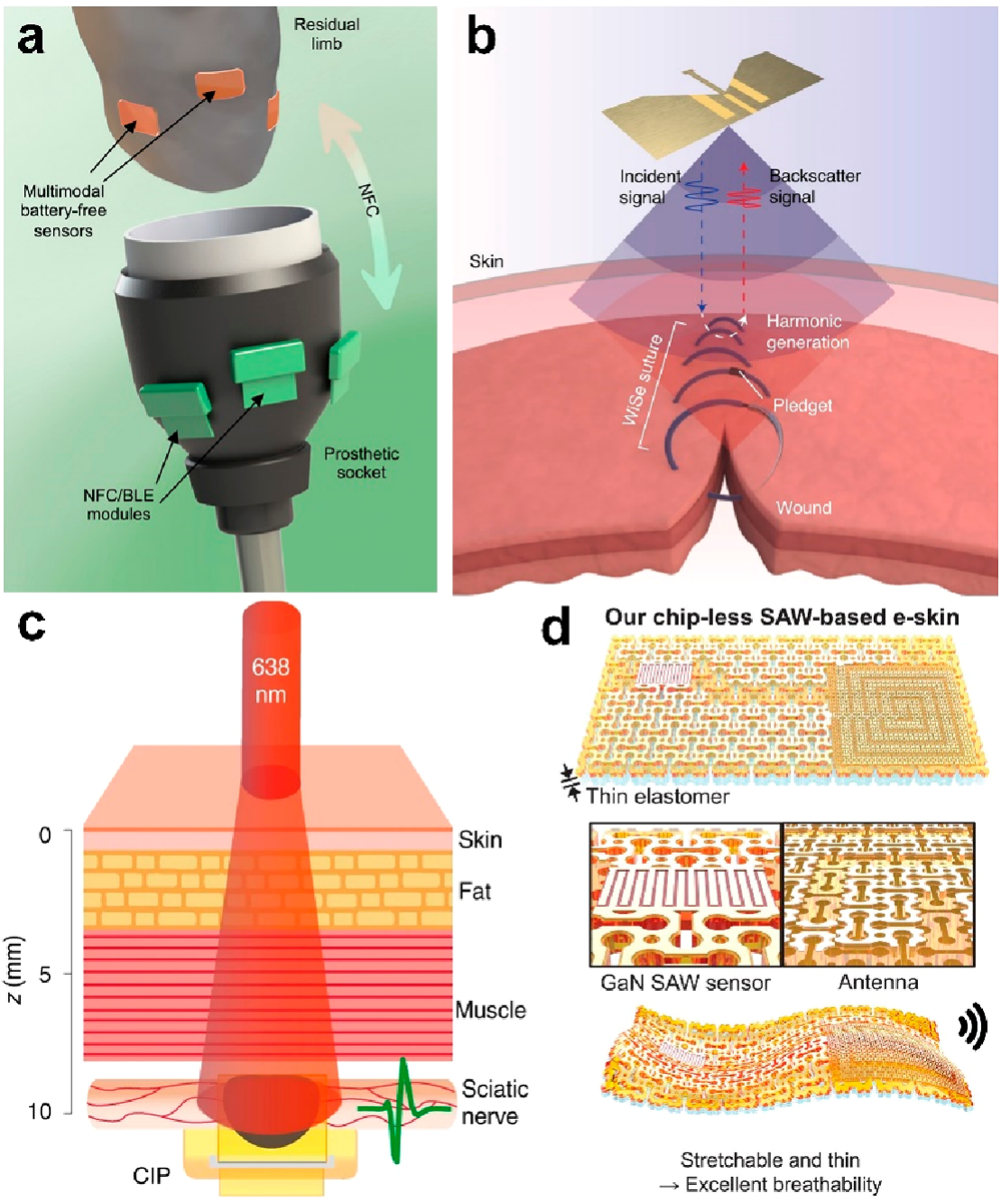
Other powering technologies in wearable applications. (a) NFC powering. Reproduced with permission from ref (33). Copyright 2020 AAAS. (b) LC-oscillation powering. Reproduced with permission from ref (231). Copyright 2021 The Authors. (c) IR powering. Reproduced with permission from ref (232). Copyright 2021 The Authors. (d) Chipless SAW-based e-skin. Reproduced with permission from ref (233). Copyright 2022 AAAS.

Although researchers have attempted to find techniques to power wearable sensors, work is still required to further improve the power output for the increasing monitoring demands. Therefore, some recent novel powering technologies from outside are disclosed. Figure 25a shows a power technology via near-field communication (NFC),^33^ which has been widely utilized in power supply for wearable or implantable electronics in recent years.^228−230^ Specifically, there are two components: the soft pressure sensor is attached on human skin, while the power components are located on the prosthetic. Two components utilized NFC coils for power and sensing data transmission. This approach ensures skin compliance and the sustainable power supply. A similar methodology has been extended to radio frequency (rf) powering,^231^ as shown in Figure 25b. It aims to monitor wound healing, where the suture is engineered with an electrode, serving as a rf antenna, and equipped with a variable resistor. When the wound changes, the resistor will change. Therefore, we can use rf transmission to transmit electricity to the suture and then obtain the resistor changing from the reflected signal. Figure 25c shows an example that employs infrared radiation to transmit energy to the chip implanted in the living creature, which can generate an electric signal for nerve stimulation.^232^Figure 25d illustrates a system that use surface acoustic wave to get energy and sensing data transmission.^233^ Specifically, they present a chipless wireless patch made of freestanding single-crystalline piezoelectric gallium nitride membranes, forming a surface acoustic wave (SAW) device. And, wireless strain sensing can be done by calibrating the resonant peak shifts in the SAW device in response to strain induced by bending the patch.

We show these examples using wireless energy transmission to solve the wearable electronics’ power consumption problem, which could be an alternative approach where self-powered technology is not applicable.

## 5 Challenges and Perspectives

**Figure 26:**
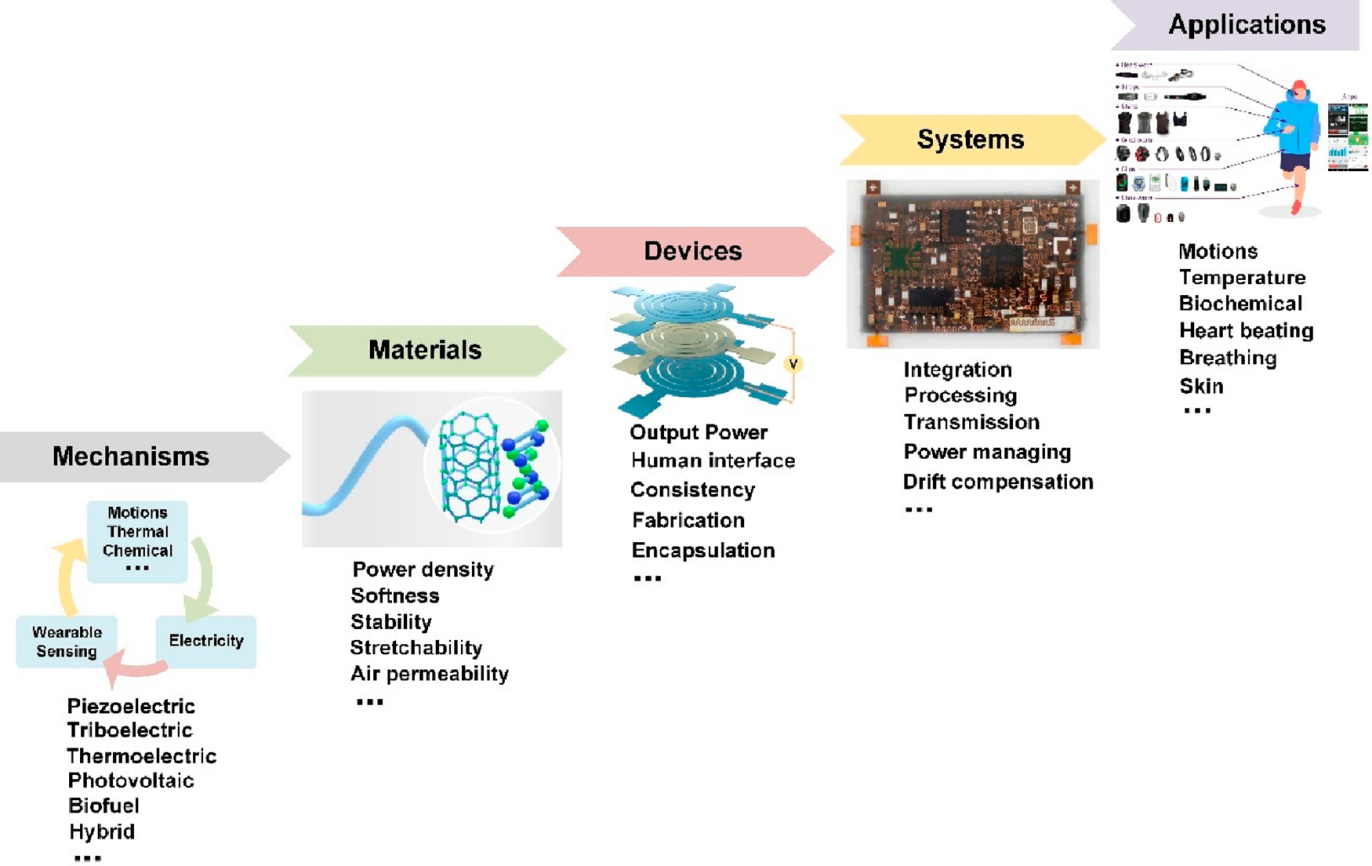
Perspectives on self-powered wearables’ future development. Reproduced with permission from ref (235). Copyright 2018 The Authors.

This review highlights the significant advancements made in wearable power generation technology in recent years. The self-powered approach is particularly appealing, yet it presents its own set of challenges. Currently, two primary strategies have been employed: self-powered sensors and self-powered systems. A variety of self-powered devices have been developed; however, the current power output remains somewhat insufficient. As the demand for wearable monitoring continues to grow, the need for increased power generation becomes more pressing. Consequently, innovative technologies must be explored, addressing key research aspects such as mechanisms, materials, devices, and systems in order to overcome specific issues and limitations (Figure 26).

Mechanisms: One of the primary challenges in self-powered wearable sensing systems is efficiently harvesting energy from the environment or the user’s movements. Various energy sources are discussed above, including piezoelectric, triboelectric, thermoelectric, and photovoltaic, among others. Developing innovative materials and mechanisms to optimize energy harvesting or sensing approaches will be crucial.

Materials: A key consideration involves the development of soft materials for high-power harvesting devices. For example, PVDF is widely utilized now in PENGs due to its high piezoelectric coefficient and flexibility. Researchers should determine methods to further enhance these properties while maintaining softness and stability. In terms of triboelectric materials, although numerous options exist, high electrification density materials are in demand, and meanwhile, the fabrication complexity must be taken into account. Additionally, there is also a demand for developing high-performance soft thermoelectric and photovoltaic materials. Besides, compliance between the materials and the human body should be considered, including stretchability, softness, as well as breathability. In our opinion, it is anticipated that more bionic materials might be investigated and exploited.

Devices: Since these devices are based on new materials, standardized and scalable fabrication processes should be developed in order to ensure the devices’ consistency and stability in practical applications. Besides, more investigations on device structure design, for high-efficiency coupling with human motions, heat, biochemical energy, etc., are required. As for self-powered sensors, the precision is always affected by factors such as the material’s viscoelasticity, fatigue, and environmental variations.^222^ Techniques are required to diminish that influence, for instance, via encapsulation or circuit compensation. Although displacement-sensitive sensors provide high precision and stability, there is a need for prototypes with extended functionality to accommodate diverse applications, such as force or pressure sensing.

Systems: Circuitry plays a crucial role in compensating for sensor drift and managing power. Given that the energy pulses generated are typically discontinuous and exhibit varying voltage ranges, power management circuits are vital for adjusting the output, storing energy in conventional storage mediums, and providing power to the entire system. Moreover, signal processing and transmission circuits are also in demand. Finally, proper integration and packaging methods should be investigated. Furthermore, exploring novel monitoring principles, such as surface acoustic wave (SAW)-based sensing systems that eliminate the need for chips and batteries, may offer solutions for particular applications.

In summary, it is always a goal for enhancing the output of various power generation devices, from mechanisms, materials, design optimizations to system integrations, so as to ensure the sustainable operation of emerging wearable devices. With the advancement of micro/nanoelectronics and power technologies, we anticipate the development of multifunctional wearable devices and systems that will provide more comfortable and reliable health monitoring for individuals. Concurrently, the gap between system consumption and power supply is expected to narrow, ultimately leading to a paradigm shift toward self-powered wearable sensing and systems.

## References

[ref1] Dunn J.; Kidzinski L.; Runge R.; Witt D.; Hicks J. L.; Schüssler-Fiorenza Rose S. M.; Li X.; Bahmani A.; Delp S. L.; Hastie T.; et al. Wearable Sensors Enable Personalized Predictions of Clinical Laboratory Measurements. Nat. Med. 2021, 27, 1105–1112. 10.1038/s41591-021-01339-0. 34031607 PMC8293303

[ref2] Ray T. R.; Choi J.; Bandodkar A. J.; Krishnan S.; Gutruf P.; Tian L.; Ghaffari R.; Rogers J. A. Bio-Integrated Wearable Systems: A Comprehensive Review. Chem. Rev. 2019, 119, 5461–5533. 10.1021/acs.chemrev.8b00573. 30689360

[ref3] Luo Y.; Abidian M. R.; Ahn J.-H.; Akinwande D.; Andrews A. M.; Antonietti M.; Bao Z.; Berggren M.; Berkey C. A.; Bettinger C. J.; et al. Technology Roadmap for Flexible Sensors. ACS Nano 2023, 17, 5211–5295. 10.1021/acsnano.2c12606. 36892156 PMC11223676

[ref4] Fernandez S. V.; Sadat D.; Tasnim F.; Acosta D.; Schwendeman L.; Shahsavari S.; Dagdeviren C. Ubiquitous Conformable Systems for Imperceptible Computing. Foresight 2022, 24, 75–98. 10.1108/FS-07-2020-0067.

[ref5] Tasnim F.; Sadraei A.; Datta B.; Khan M.; Choi K. Y.; Sahasrabudhe A.; Vega Galvez T. A.; Wicaksono I.; Rosello O.; Nunez-Lopez C.; Dagdeviren C.; et al. Towards Personalized Medicine: The Evolution of Imperceptible Health-Care Technologies. Foresight 2018, 20, 589–601. 10.1108/FS-08-2018-0075.

[ref6] Bayoumy K.; Gaber M.; Elshafeey A.; Mhaimeed O.; Dineen E. H.; Marvel F. A.; Martin S. S.; Muse E. D.; Turakhia M. P.; Tarakji K. G.; et al. Smart Wearable Devices in Cardiovascular Care: Where We Are and How to Move Forward. Nat. Rev. Cardiol 2021, 18, 581–599. 10.1038/s41569-021-00522-7. 33664502 PMC7931503

[ref7] Nelson B. W.; Low C. A.; Jacobson N.; Areán P.; Torous J.; Allen N. B. Guidelines for Wrist-Worn Consumer Wearable Assessment of Heart Rate in Biobehavioral Research. npj Digit. Med. 2020, 3, 90 10.1038/s41746-020-0297-4. 32613085 PMC7320189

[ref8] Shcherbina A.; Mattsson C. M.; Waggott D.; Salisbury H.; Christle J. W.; Hastie T.; Wheeler M. T.; Ashley E. A. Accuracy in Wrist-Worn, Sensor-Based Measurements of Heart Rate and Energy Expenditure in a Diverse Cohort. J. Pers. Med. 2017, 7, 3 10.3390/jpm7020003. 28538708 PMC5491979

[ref9] Li F.; Xue H.; Lin X.; Zhao H.; Zhang T. Wearable Temperature Sensor with High Resolution for Skin Temperature Monitoring. ACS Appl. Polym. Mater. 2022, 14, 43844–43852. 10.1021/acsami.2c15687. 36124623

[ref10] Han S.; Kim J.; Won S. M.; Ma Y.; Kang D.; Xie Z.; Lee K.-T.; Chung H. U.; Banks A.; Min S. Battery-Free, Wireless Sensors for Full-Body Pressure and Temperature Mapping. Sci. Transl. Med. 2018, 10, eaan4950 10.1126/scitranslmed.aan4950. 29618561 PMC5996377

[ref11] Honda W.; Harada S.; Arie T.; Akita S.; Takei K. In Printed wearable temperature sensor for health monitoring. SENSORS, 2014 IEEE, Valencia, Spain; IEEE, 2014; pp 2227–2229 10.1109/ICSENS.2014.6985483.

[ref12] Kim J.-N.; Lee J.; Lee H.; Oh I.-K. Stretchable and Self-Healable Catechol-Chitosan-Diatom Hydrogel for Triboelectric Generator and Self-Powered Tremor Sensor Targeting at Parkinson Disease. Nano Energy 2021, 82, 105705 10.1016/j.nanoen.2020.105705.

[ref13] Chung H. U.; Rwei A. Y.; Hourlier-Fargette A.; Xu S.; Lee K.; Dunne E. C.; Xie Z.; Liu C.; Carlini A.; Kim D. H.; et al. Skin-Interfaced Biosensors for Advanced Wireless Physiological Monitoring in Neonatal and Pediatric Intensive-Care Units. Nat. Med. 2020, 26, 418–429. 10.1038/s41591-020-0792-9. 32161411 PMC7315772

[ref14] Kim K. K.; Kim M.; Pyun K.; Kim J.; Min J.; Koh S.; Root S. E.; Kim J.; Nguyen B.-N. T.; Nishio Y.; et al. A Substrate-Less Nanomesh Receptor with Meta-Learning for Rapid Hand Task Recognition. Nat. Electron. 2022, 6, 64–75. 10.1038/s41928-022-00888-7.

[ref15] Bariya M.; Nyein H. Y. Y.; Javey A. Wearable Sweat Sensors. Nat. Electron. 2018, 1, 160–171. 10.1038/s41928-018-0043-y.

[ref16] Yang Y.; Song Y.; Bo X.; Min J.; Pak O. S.; Zhu L.; Wang M.; Tu J.; Kogan A.; Zhang H.; et al. A Laser-Engraved Wearable Sensor for Sensitive Detection of Uric Acid and Tyrosine in Sweat. Nat. Biotechnol. 2020, 38, 217–224. 10.1038/s41587-019-0321-x. 31768044

[ref17] Min J.; Tu J.; Xu C.; Lukas H.; Shin S.; Yang Y.; Solomon S. A.; Mukasa D.; Gao W. Skin-Interfaced Wearable Sweat Sensors for Precision Medicine. Chem. Rev. 2023, 123, 5049–5138. 10.1021/acs.chemrev.2c00823. 36971504 PMC10406569

[ref18] Güntner A. T.; Abegg S.; Königstein K.; Gerber P. A.; Schmidt-Trucksäss A.; Pratsinis S. E. Breath Sensors for Health Monitoring. ACS Sens. 2019, 4, 268–280. 10.1021/acssensors.8b00937. 30623644

[ref19] Bao W.; Chen F.; Lai H.; Liu S.; Wang Y. Wearable Breath Monitoring Based on a Flexible Fiber-Optic Humidity Sensor. Sens. Actuators B Chem. 2021, 349, 130794 10.1016/j.snb.2021.130794.

[ref20] Sel K.; Osman D.; Huerta N.; Edgar A.; Pettigrew R. I.; Jafari R. Continuous Cuffless Blood Pressure Monitoring with a Wearable Ring Bioimpedance Device. npj Digit. Med. 2023, 6, 59 10.1038/s41746-023-00796-w. 36997608 PMC10063561

[ref21] Konstantinidis D.; Iliakis P.; Tatakis F.; Thomopoulos K.; Dimitriadis K.; Tousoulis D.; Tsioufis K. Wearable Blood Pressure Measurement Devices and New Approaches in Hypertension Management: The Digital Era. J. Hum Hypertens. 2022, 36, 945–951. 10.1038/s41371-022-00675-z. 35322181 PMC8942176

[ref22] Song E.; Xie Z.; Bai W.; Luan H.; Ji B.; Ning X.; Xia Y.; Baek J. M.; Lee Y.; Avila R.; et al. Miniaturized Electromechanical Devices for the Characterization of the Biomechanics of Deep Tissue. Nat. Biomed. Eng. 2021, 5, 759–771. 10.1038/s41551-021-00723-y. 34045731

[ref23] Wang Z. L. Self-Powered Nanotech. Sci. Am. 2008, 298, 82–87. 10.1038/scientificamerican0108-82. 18225699

[ref24] Wang Z. L.; Song J. Piezoelectric Nanogenerators Based on Zinc Oxide Nanowire Arrays. Science 2006, 312, 242–246. 10.1126/science.1124005. 16614215

[ref25] Wang Z. L. Toward Self-Powered Sensor Networks. Nano Today 2010, 5, 512–514. 10.1016/j.nantod.2010.09.001.

[ref26] Wang Z. L. Self-Powered Nanosensors and Nanosystems. Adv. Mater. 2012, 24, 280–285. 10.1002/adma.201102958. 22329002

[ref27] Hu Y.; Lin L.; Zhang Y.; Wang Z. L. Replacing a Battery by a Nanogenerator with 20 V Output. Adv. Mater. 2012, 24, 110–114. 10.1002/adma.201103727. 22057731

[ref28] Fan F.-R.; Tian Z.-Q.; Lin Wang Z. Flexible Triboelectric Generator. Nano Energy 2012, 1, 328–334. 10.1016/j.nanoen.2012.01.004.

[ref29] Wang S.; Lin Z.-H.; Niu S.; Lin L.; Xie Y.; Pradel K. C.; Wang Z. L. Motion Charged Battery as Sustainable Flexible-Power-Unit. ACS Nano 2013, 7, 11263–11271. 10.1021/nn4050408. 24266595

[ref30] Chen J.; Huang Y.; Zhang N.; Zou H.; Liu R.; Tao C.; Fan X.; Wang Z. L. Micro-Cable Structured Textile for Simultaneously Harvesting Solar and Mechanical Energy. Nat. Energy 2016, 1, 16138 10.1038/nenergy.2016.138.

[ref31] Guo H.; Pu X.; Chen J.; Meng Y.; Yeh M.-H.; Liu G.; Tang Q.; Chen B.; Liu D.; Qi S. A Highly Sensitive, Self-Powered Triboelectric Auditory Sensor for Social Robotics and Hearing Aids. Sci. Robot. 2018, 3, eaat2516 10.1126/scirobotics.aat2516. 33141730

[ref32] Ouyang H.; Liu Z.; Li N.; Shi B.; Zou Y.; Xie F.; Ma Y.; Li Z.; Li H.; Zheng Q. Symbiotic Cardiac Pacemaker. Nat. Commun. 2019, 10, 1821 10.1038/s41467-019-09851-1. 31015519 PMC6478903

[ref33] Yu Y.; Nassar J.; Xu C.; Min J.; Yang Y.; Dai A.; Doshi R.; Huang A.; Song Y.; Gehlhar R.; et al. Biofuel-powered soft electronic skin with multiplexed and wireless sensing for human-machine interfaces. Sci. Robot. 2020, 5, eaaz7946 10.1126/scirobotics.aaz7946. 32607455 PMC7326328

[ref34] Zhou Y.; Fei C.; Uddin M. A.; Zhao L.; Ni Z.; Huang J. Self-Powered Perovskite Photon-Counting Detectors. Nature 2023, 616, 712–718. 10.1038/s41586-023-05847-6. 37020031 PMC11565494

[ref35] Jaffe H. Piezoelectric Ceramics. J. Am. Ceram. Soc. 1958, 41, 494–498. 10.1111/j.1151-2916.1958.tb12903.x.

[ref36] Tressler J. F.; Alkoy S.; Newnham R. E. Piezoelectric Sensors and Sensor Materials. Journal of Electroceramics 1998, 2, 257–272. 10.1023/A:1009926623551.

[ref37] Krautkramer J., Krautkramer H. Ultrasonic Testing of Materials; Springer-Verlag, 1990; p 677.

[ref38] Manbachi A.; Cobbold R. S. C. Development and Application of Piezoelectric Materials for Ultrasound Generation and Detection. Ultrasound 2011, 19, 187–196. 10.1258/ult.2011.011027.

[ref39] Kim J. D.; Choi J. S.; Kim B. S.; Chan Choi Y.; Cho Y. W. Piezoelectric Inkjet Printing of Polymers: Stem Cell Patterning on Polymer Substrates. Polymer 2010, 51, 2147–2154. 10.1016/j.polymer.2010.03.038.

[ref40] Jung S.-B.; Kim S.-W. Improvement of Scanning Accuracy of Pzt Piezoelectric Actuators by Feed-Forward Model-Reference Control. Precision Engineering 1994, 16, 49–55. 10.1016/0141-6359(94)90018-3.

[ref41] Karrai K.; Grober R. D. Piezoelectric Tip-Sample Distance Control for near Field Optical Microscopes. Appl. Phys. Lett. 1995, 66, 1842–1844. 10.1063/1.113340.

[ref42] Kawashima S.; Ohnishi O.; Hakamata H.; Tagami F.; Fukuoka A.; Inoue T.; Hirose S. Third order longitudinal mode piezoelectric ceramic transformer and its application to high-voltage power inverter. Proceedings of IEEE Ultrasonics Symposium ULTSYM-94; IEEE, 1994; Vol. 1, pp 525–530.

[ref43] Gao Z.; Zhou J.; Gu Y.; Fei P.; Hao Y.; Bao G.; Wang Z. L. Effects of Piezoelectric Potential on the Transport Characteristics of Metal-Zno Nanowire-Metal Field Effect Transistor. J. Appl. Phys. 2009, 105, 113707 10.1063/1.3125449. 19657403 PMC2719466

[ref44] Wang Z. L.; Yang R.; Zhou J.; Qin Y.; Xu C.; Hu Y.; Xu S. Lateral Nanowire/Nanobelt Based Nanogenerators, Piezotronics and Piezo-Phototronics. Mater. Sci. Eng. R Rep. 2010, 70, 320–329. 10.1016/j.mser.2010.06.015.

[ref45] Wang X.; Song J.; Liu J.; Wang Z. L. Direct-Current Nanogenerator Driven by Ultrasonic Waves. Science 2007, 316, 102–105. 10.1126/science.1139366. 17412957

[ref46] Xu S.; Wei Y.; Liu J.; Yang R.; Wang Z. L. Integrated Multilayer Nanogenerator Fabricated Using Paired Nanotip-to-Nanowire Brushes. Nano Lett. 2008, 8, 4027–4032. 10.1021/nl8027813. 18939811

[ref47] Zhou J.; Fei P.; Gao Y.; Gu Y.; Liu J.; Bao G.; Wang Z. L. Mechanical–Electrical Triggers and Sensors Using Piezoelectric Micowires/Nanowires. Nano Lett. 2008, 8, 2725–2730. 10.1021/nl8010484. 18681485

[ref48] Yang R.; Qin Y.; Dai L.; Wang Z. L. Power Generation with Laterally Packaged Piezoelectric Fine Wires. Nat. Nanotechnol. 2009, 4, 34–39. 10.1038/nnano.2008.314. 19119280

[ref49] Ray T. R.; Choi J.; Bandodkar A. J.; Krishnan S.; Gutruf P.; Tian L.; Ghaffari R.; Rogers J. A. Bio-Integrated Wearable Systems: A Comprehensive Review. Chem. Rev. 2019, 119, 5461–5533. 10.1021/acs.chemrev.8b00573. 30689360

[ref50] Yamada T.; Ueda T.; Kitayama T. Piezoelectricity of a High-Content Lead Zirconate Titanate/Polymer Composite. J. Appl. Phys. 1982, 53, 4328–4332. 10.1063/1.331211.

[ref51] Chang C.; Tran V. H.; Wang J.; Fuh Y.-K.; Lin L. Direct-Write Piezoelectric Polymeric Nanogenerator with High Energy Conversion Efficiency. Nano Lett. 2010, 10, 726–731. 10.1021/nl9040719. 20099876

[ref52] Kawai H. The Piezoelectricity of Poly (Vinylidene Fluoride). Jpn. J. Appl. Phys. 1969, 8, 975 10.1143/JJAP.8.975.

[ref53] Scheffler S.; Poulin P. Piezoelectric Fibers: Processing and Challenges. ACS Appl. Mater. Interfaces. 2022, 14, 16961–16982. 10.1021/acsami.1c24611. 35404561

[ref54] Qi Y.; Kim J.; Nguyen T. D.; Lisko B.; Purohit P. K.; McAlpine M. C. Enhanced Piezoelectricity and Stretchability in Energy Harvesting Devices Fabricated from Buckled Pzt Ribbons. Nano Lett. 2011, 11, 1331–1336. 10.1021/nl104412b. 21322604

[ref55] Jeong C. K.; Kim I.; Park K.-I.; Oh M. H.; Paik H.; Hwang G.-T.; No K.; Nam Y. S.; Lee K. J. Virus-Directed Design of a Flexible Batio3 Nanogenerator. ACS Nano 2013, 7, 11016–11025. 10.1021/nn404659d. 24229091

[ref56] Wu W.; Wang L.; Li Y.; Zhang F.; Lin L.; Niu S.; Chenet D.; Zhang X.; Hao Y.; Heinz T. F.; et al. Piezoelectricity of Single-Atomic-Layer Mos2 for Energy Conversion and Piezotronics. Nature 2014, 514, 470–474. 10.1038/nature13792. 25317560

[ref57] Liu Y.; Dzidotor G.; Le T. T.; Vinikoor T.; Morgan K.; Curry E. J.; Das R.; McClinton A.; Eisenberg E.; Apuzzo L. N.; et al. Exercise-Induced Piezoelectric Stimulation for Cartilage Regeneration in Rabbits. Sci. Transl Med. 2022, 14, eabi7282 10.1126/scitranslmed.abi7282. 35020409

[ref58] Choi D.; Choi M.-Y.; Choi W. M.; Shin H.-J.; Park H.-K.; Seo J.-S.; Park J.; Yoon S.-M.; Chae S. J.; Lee Y. H.; et al. Fully Rollable Transparent Nanogenerators Based on Graphene Electrodes. Adv. Mater. 2010, 22, 2187–2192. 10.1002/adma.200903815. 20376853

[ref59] Yang F.; Li J.; Long Y.; Zhang Z.; Wang L.; Sui J.; Dong Y.; Wang Y.; Taylor R.; Ni D.; et al. Wafer-Scale Heterostructured Piezoelectric Bio-Organic Thin Films. Science 2021, 373, 337–342. 10.1126/science.abf2155. 34437153 PMC8516594

[ref60] Schäufele A. B.; Heinz Härdtl K. Ferroelastic Properties of Lead Zirconate Titanate Ceramics. J. Am. Ceram. Soc. 1996, 79, 2637–2640. 10.1111/j.1151-2916.1996.tb09027.x.

[ref61] Wu W.; Wang L.; Li Y.; Zhang F.; Lin L.; Niu S.; Chenet D.; Zhang X.; Hao Y.; Heinz T. F.; et al. Piezoelectricity of Single-Atomic-Layer Mos2 for Energy Conversion and Piezotronics. Nature 2014, 514, 470–474. 10.1038/nature13792. 25317560

[ref62] Yang R.; Qin Y.; Li C.; Zhu G.; Wang Z. L. Converting Biomechanical Energy into Electricity by a Muscle-Movement-Driven Nanogenerator. Nano Lett. 2009, 9, 1201–1205. 10.1021/nl803904b. 19203203

[ref63] Park J.-H.; Jang D.-G.; Park J. W.; Youm S.-K. Wearable Sensing of In-Ear Pressure for Heart Rate Monitoring with a Piezoelectric Sensor. Sensors 2015, 15, 23402–23417. 10.3390/s150923402. 26389912 PMC4610448

[ref64] Yan Z.; Pan T.; Wang D.; Li J.; Jin L.; Huang L.; Jiang J.; Qi Z.; Zhang H.; Gao M.; et al. Stretchable Micromotion Sensor with Enhanced Sensitivity Using Serpentine Layout. ACS Appl. Polym. Mater. 2019, 11, 12261–12271. 10.1021/acsami.8b22613. 30807090

[ref65] Liu D.; Zhang D.; Sun Z.; Zhou S.; Li W.; Li C.; Li W.; Tang W.; Wang Z. L. Active-Matrix Sensing Array Assisted with Machine-Learning Approach for Lumbar Degenerative Disease Diagnosis and Postoperative Assessment. Adv. Funct. Mater. 2022, 32, 2113008 10.1002/adfm.202113008.

[ref66] Han M.; Wang H.; Yang Y.; Liang C.; Bai W.; Yan Z.; Li H.; Xue Y.; Wang X.; Akar B.; et al. Three-Dimensional Piezoelectric Polymer Microsystems for Vibrational Energy Harvesting, Robotic Interfaces and Biomedical Implants. Nat. Electron. 2019, 2, 26–35. 10.1038/s41928-018-0189-7.

[ref67] Wang Z. L. Triboelectric Nanogenerators as New Energy Technology for Self-Powered Systems and as Active Mechanical and Chemical Sensors. ACS Nano 2013, 7, 9533–9557. 10.1021/nn404614z. 24079963

[ref68] Wang Z. L. Triboelectric Nanogenerator (Teng)—Sparking an Energy and Sensor Revolution. Adv. Energy Mater. 2020, 10, 2000137 10.1002/aenm.202000137.

[ref69] Wang Z. L.; Chen J.; Lin L. Progress in Triboelectric Nanogenerators as a New Energy Technology and Self-Powered Sensors. Energy Environ. Sci. 2015, 8, 2250–2282. 10.1039/C5EE01532D.

[ref70] Zhu G.; Pan C.; Guo W.; Chen C.-Y.; Zhou Y.; Yu R.; Wang Z. L. Triboelectric-Generator-Driven Pulse Electrodeposition for Micropatterning. Nano Lett. 2012, 12, 4960–4965. 10.1021/nl302560k. 22889363

[ref71] Lin L.; Wang S.; Xie Y.; Jing Q.; Niu S.; Hu Y.; Wang Z. L. Segmentally Structured Disk Triboelectric Nanogenerator for Harvesting Rotational Mechanical Energy. Nano Lett. 2013, 13, 2916–2923. 10.1021/nl4013002. 23656350

[ref72] Wang S.; Xie Y.; Niu S.; Lin L.; Wang Z. L. Freestanding Triboelectric-Layer-Based Nanogenerators for Harvesting Energy from a Moving Object or Human Motion in Contact and Non-Contact Modes. Adv. Mater. 2014, 26, 2818–2824. 10.1002/adma.201305303. 24449058

[ref73] Wang S.; Lin L.; Wang Z. L. Nanoscale Triboelectric-Effect-Enabled Energy Conversion for Sustainably Powering Portable Electronics. Nano Lett. 2012, 12, 6339–6346. 10.1021/nl303573d. 23130843

[ref74] Zhu G.; Su Y.; Bai P.; Chen J.; Jing Q.; Yang W.; Wang Z. L. Harvesting Water Wave Energy by Asymmetric Screening of Electrostatic Charges on a Nanostructured Hydrophobic Thin-Film Surface. ACS Nano 2014, 8, 6031–6037. 10.1021/nn5012732. 24745893

[ref75] Zhu G.; Chen J.; Zhang T.; Jing Q.; Wang Z. L. Radial-Arrayed Rotary Electrification for High Performance Triboelectric Generator. Nat. Commun. 2014, 5, 3426 10.1038/ncomms4426. 24594501

[ref76] Niu S.; Wang X.; Yi F.; Zhou Y. S.; Wang Z. L. A Universal Self-Charging System Driven by Random Biomechanical Energy for Sustainable Operation of Mobile Electronics. Nat. Commun. 2015, 6, 8975 10.1038/ncomms9975. 26656252 PMC4682168

[ref77] Fan F.-R.; Lin L.; Zhu G.; Wu W.; Zhang R.; Wang Z. L. Transparent Triboelectric Nanogenerators and Self-Powered Pressure Sensors Based on Micropatterned Plastic Films. Nano Lett. 2012, 12, 3109–3114. 10.1021/nl300988z. 22577731

[ref78] Zhou Y. S.; Zhu G.; Niu S.; Liu Y.; Bai P.; Jing Q.; Wang Z. L. Nanometer Resolution Self-Powered Static and Dynamic Motion Sensor Based on Micro-Grated Triboelectrification. Adv. Mater. 2014, 26, 1719–1724. 10.1002/adma.201304619. 24375783

[ref79] Chen J.; Zhu G.; Yang J.; Jing Q.; Bai P.; Yang W.; Qi X.; Su Y.; Wang Z. L. Personalized Keystroke Dynamics for Self-Powered Human–Machine Interfacing. ACS Nano 2015, 9, 105–116. 10.1021/nn506832w. 25552331

[ref80] Luo J.; Tang W.; Fan F. R.; Liu C.; Pang Y.; Cao G.; Wang Z. L. Transparent and Flexible Self-Charging Power Film and Its Application in a Sliding Unlock System in Touchpad Technology. ACS Nano 2016, 10, 8078–8086. 10.1021/acsnano.6b04201. 27501289

[ref81] Wang Z. L. On Maxwell’s Displacement Current for Energy and Sensors: The Origin of Nanogenerators. Mater. Today 2017, 20, 74–82. 10.1016/j.mattod.2016.12.001.

[ref82] Wang Z. L. On the Expanded Maxwell’s Equations for Moving Charged Media System – General Theory, Mathematical Solutions and Applications in Teng. Mater. Today 2022, 52, 348–363. 10.1016/j.mattod.2021.10.027.

[ref83] Wang Z. L. The Expanded Maxwell’s Equations for a Mechano-Driven Media System That Moves with Acceleration. Int. J. Mod. Phys. B 2023, 37, 16 10.1142/S021797922350159X.

[ref84] Wang Z. L. Maxwell’s Equations for a Mechano-Driven, Shape-Deformable, Charged-Media System, Slowly Moving at an Arbitrary Velocity Field V(R, T). J. Phys. Commun. 2022, 6, 085013 10.1088/2399-6528/ac871e.

[ref85] Davies D. K. Charge Generation on Dielectric Surfaces. J. Phys. D Appl. Phys. 1969, 2, 1533–1537. 10.1088/0022-3727/2/11/307.

[ref86] McCarty L. S.; Whitesides G. M. Electrostatic Charging Due to Separation of Ions at Interfaces: Contact Electrification of Ionic Electrets. Angew. Chem., Int. Ed. Engl. 2008, 47, 2188–2207. 10.1002/anie.200701812. 18270989

[ref87] Lowell J.; Rose-Innes A. C. Contact Electrification. Adv. Phys. 1980, 29, 947–1023. 10.1080/00018738000101466.

[ref88] Xu C.; Zi Y.; Wang A. C.; Zou H.; Dai Y.; He X.; Wang P.; Wang Y.-C.; Feng P.; Li D.; et al. On the Electron-Transfer Mechanism in the Contact-Electrification Effect. Adv. Mater. 2018, 30, 1706790 10.1002/adma.201706790. 29508454

[ref89] Lin S.; Xu L.; Xu C.; Chen X.; Wang A. C.; Zhang B.; Lin P.; Yang Y.; Zhao H.; Wang Z. L. Electron Transfer in Nanoscale Contact Electrification: Effect of Temperature in the Metal–Dielectric Case. Adv. Mater. 2019, 31, 1808197 10.1002/adma.201808197. 30844100

[ref90] Xu C.; Wang A. C.; Zou H.; Zhang B.; Zhang C.; Zi Y.; Pan L.; Wang P.; Feng P.; Lin Z.; et al. Raising the Working Temperature of a Triboelectric Nanogenerator by Quenching Down Electron Thermionic Emission in Contact-Electrification. Adv. Mater. 2018, 30, 1803968 10.1002/adma.201803968. 30091484

[ref91] Lin S.; Xu L.; Chi Wang A.; Wang Z. L. Quantifying Electron-Transfer in Liquid-Solid Contact Electrification and the Formation of Electric Double-Layer. Nat. Commun. 2020, 11, 399 10.1038/s41467-019-14278-9. 31964882 PMC6972942

[ref92] Tang W.; Jiang T.; Fan F. R.; Yu A. F.; Zhang C.; Cao X.; Wang Z. L. Liquid-Metal Electrode for High-Performance Triboelectric Nanogenerator at an Instantaneous Energy Conversion Efficiency of 70.6%. Adv. Funct. Mater. 2015, 25, 3718–3725. 10.1002/adfm.201501331.

[ref93] Nie J.; Wang Z.; Ren Z.; Li S.; Chen X.; Lin Wang Z. Power Generation from the Interaction of a Liquid Droplet and a Liquid Membrane. Nat. Commun. 2019, 10, 2264 10.1038/s41467-019-10232-x. 31118419 PMC6531479

[ref94] Qin H.; Xu L.; Lin S.; Zhan F.; Dong K.; Han K.; Wang H.; Feng Y.; Wang Z. L. Underwater Energy Harvesting and Sensing by Sweeping out the Charges in an Electric Double Layer Using an Oil Droplet. Adv. Funct. Mater. 2022, 32, 2111662 10.1002/adfm.202111662.

[ref95] Sun L.; Wang Z.; Li C.; Tang W.; Wang Z. Probing Contact Electrification between Gas and Solid Surface. Nanoenergy Adv. 2023, 3, 1–11. 10.3390/nanoenergyadv3010001.

[ref96] Wu C.; Wang A. C.; Ding W.; Guo H.; Wang Z. L. Triboelectric Nanogenerator: A Foundation of the Energy for the New Era. Adv. Energy Mater. 2019, 9, 1802906 10.1002/aenm.201802906.

[ref97] Wang S.; Lin L.; Wang Z. L. Triboelectric Nanogenerators as Self-Powered Active Sensors. Nano Energy 2015, 11, 436–462. 10.1016/j.nanoen.2014.10.034.

[ref98] Zhou Y.; Shen M.; Cui X.; Shao Y.; Li L.; Zhang Y. Triboelectric Nanogenerator Based Self-Powered Sensor for Artificial Intelligence. Nano Energy 2021, 84, 105887 10.1016/j.nanoen.2021.105887.

[ref99] Lei H.; Chen Y.; Gao Z.; Wen Z.; Sun X. Advances in Self-Powered Triboelectric Pressure Sensors. J. Mater. Chem. A 2021, 9, 20100–20130. 10.1039/D1TA03505C.

[ref100] Zhang X.-S.; Han M.-D.; Wang R.-X.; Zhu F.-Y.; Li Z.-H.; Wang W.; Zhang H.-X. Frequency-Multiplication High-Output Triboelectric Nanogenerator for Sustainably Powering Biomedical Microsystems. Nano Lett. 2013, 13, 1168–1172. 10.1021/nl3045684. 23384278

[ref101] Tang W.; Tian J.; Zheng Q.; Yan L.; Wang J.; Li Z.; Wang Z. L. Implantable Self-Powered Low-Level Laser Cure System for Mouse Embryonic Osteoblasts’ Proliferation and Differentiation. ACS Nano 2015, 9, 7867–7873. 10.1021/acsnano.5b03567. 26161869

[ref102] Park S.; Kim H.; Vosgueritchian M.; Cheon S.; Kim H.; Koo J. H.; Kim T. R.; Lee S.; Schwartz G.; Chang H.; et al. Stretchable Energy-Harvesting Tactile Electronic Skin Capable of Differentiating Multiple Mechanical Stimuli Modes. Adv. Mater. 2014, 26, 7324–7332. 10.1002/adma.201402574. 25256696

[ref103] Yang P.-K.; Lin Z.-H.; Pradel K. C.; Lin L.; Li X.; Wen X.; He J.-H.; Wang Z. L. Paper-Based Origami Triboelectric Nanogenerators and Self-Powered Pressure Sensors. ACS Nano 2015, 9, 901–907. 10.1021/nn506631t. 25555199

[ref104] Jang S.; Kim H.; Oh J. H. Simple and Rapid Fabrication of Pencil-on-Paper Triboelectric Nanogenerators with Enhanced Electrical Performance. Nanoscale 2017, 9, 13034–13041. 10.1039/C7NR04610C. 28836643

[ref105] Luo J.; Wang Z.; Xu L.; Wang A. C.; Han K.; Jiang T.; Lai Q.; Bai Y.; Tang W.; Fan F. R. Flexible and Durable Wood-Based Triboelectric Nanogenerators for Self-Powered Sensing in Athletic Big Data Analytics. Nat. Commun. 2019, 10, 5147 10.1038/s41467-019-13166-6. 31772189 PMC6879608

[ref106] Mao Y.; Zhang N.; Tang Y.; Wang M.; Chao M.; Liang E. A Paper Triboelectric Nanogenerator for Self-Powered Electronic Systems. Nanoscale 2017, 9, 14499–14505. 10.1039/C7NR05222G. 28930347

[ref107] Yi F.; Wang X.; Niu S.; Li S.; Yin Y.; Dai K.; Zhang G.; Lin L.; Wen Z.; Guo H.; et al. A Highly Shape-Adaptive, Stretchable Design Based on Conductive Liquid for Energy Harvesting and Self-Powered Biomechanical Monitoring. Sci. Adv. 2016, 2, e1501624 10.1126/sciadv.1501624. 27386560 PMC4928980

[ref108] Pu X.; Liu M.; Chen X.; Sun J.; Du C.; Zhang Y.; Zhai J.; Hu W.; Wang Z. L. Ultrastretchable, Transparent Triboelectric Nanogenerator as Electronic Skin for Biomechanical Energy Harvesting and Tactile Sensing. Sci. Adv. 2017, 3, e1700015 10.1126/sciadv.1700015. 28580425 PMC5451198

[ref109] Li M.; Chang K.; Zhong W.; Xiang C.; Wang W.; Liu Q.; Liu K.; Wang Y.; Lu Z.; Wang D. A Highly Stretchable, Breathable and Thermoregulatory Electronic Skin Based on the Polyolefin Elastomer Nanofiber Membrane. Appl. Surf. Sci. 2019, 486, 249–256. 10.1016/j.apsusc.2019.04.271.

[ref110] Peng X.; Dong K.; Ye C.; Jiang Y.; Zhai S.; Cheng R.; Liu D.; Gao X.; Wang J.; Wang Z. L. A Breathable, Biodegradable, Antibacterial, and Self-Powered Electronic Skin Based on All-Nanofiber Triboelectric Nanogenerators. Sci. Adv. 2020, 6, eaba9624 10.1126/sciadv.aba9624. 32637619 PMC7319766

[ref111] Zhou Z.; Chen K.; Li X.; Zhang S.; Wu Y.; Zhou Y.; Meng K.; Sun C.; He Q.; Fan W.; et al. Sign-to-Speech Translation Using Machine-Learning-Assisted Stretchable Sensor Arrays. Nat. Electron. 2020, 3, 571–578. 10.1038/s41928-020-0428-6.

[ref112] He X.; Zi Y.; Guo H.; Zheng H.; Xi Y.; Wu C.; Wang J.; Zhang W.; Lu C.; Wang Z. L. A Highly Stretchable Fiber-Based Triboelectric Nanogenerator for Self-Powered Wearable Electronics. Adv. Funct. Mater. 2017, 27, 1604378 10.1002/adfm.201604378.

[ref113] Huang T.; Wang C.; Yu H.; Wang H.; Zhang Q.; Zhu M. Human Walking-Driven Wearable All-Fiber Triboelectric Nanogenerator Containing Electrospun Polyvinylidene Fluoride Piezoelectric Nanofibers. Nano Energy 2015, 14, 226–235. 10.1016/j.nanoen.2015.01.038.

[ref114] Yang J.; Chen J.; Su Y.; Jing Q.; Li Z.; Yi F.; Wen X.; Wang Z.; Wang Z. L. Eardrum-Inspired Active Sensors for Self-Powered Cardiovascular System Characterization and Throat-Attached Anti-Interference Voice Recognition. Adv. Mater. 2015, 27, 1316–1326. 10.1002/adma.201404794. 25640534

[ref115] Wang Z.; An J.; Nie J.; Luo J.; Shao J.; Jiang T.; Chen B.; Tang W.; Wang Z. L. A Self-Powered Angle Sensor at Nanoradian-Resolution for Robotic Arms and Personalized Medicare. Adv. Mater. 2020, 32, 2001466 10.1002/adma.202001466. 32608052

[ref116] Luo J.; Li Y.; He M.; Wang Z.; Li C.; Liu D.; An J.; Xie W.; He Y.; Xiao W.; et al. Rehabilitation of Total Knee Arthroplasty by Integrating Conjoint Isometric Myodynamia and Real-Time Rotation Sensing System. Adv. Sci. 2022, 9, 2105219 10.1002/advs.202105219. PMC8922106 35038245

[ref117] Zhu M.; Sun Z.; Chen T.; Lee C. Low Cost Exoskeleton Manipulator Using Bidirectional Triboelectric Sensors Enhanced Multiple Degree of Freedom Sensory System. Nat. Commun. 2021, 12, 2692 10.1038/s41467-021-23020-3. 33976216 PMC8113469

[ref118] Li C.; Liu D.; Xu C.; Wang Z.; Shu S.; Sun Z.; Tang W.; Wang Z. L. Sensing of Joint and Spinal Bending or Stretching Via a Retractable and Wearable Badge Reel. Nat. Commun. 2021, 12, 2950 10.1038/s41467-021-23207-8. 34011979 PMC8136475

[ref119] Li C.; Xu Z.; Xu S.; Wang T.; Zhou S.; Sun Z.; Wang Z. L.; Tang W. Miniaturized Retractable Thin-Film Sensor for Wearable Multifunctional Respiratory Monitoring. Nano Res. 2023, 16, 11846 10.1007/s12274-023-5420-1. PMC9907204 36785562

[ref120] Yang L.; Li C.; Lu W.; An J.; Liu D.; Luo J.; Li Y.; Wang Z. L.; Tang W.; Meng B. High-Precision Wearable Displacement Sensing System for Clinical Diagnosis of Anterior Cruciate Ligament Tears. ACS Nano 2023, 17, 5686–5694. 10.1021/acsnano.2c11996. 36930244

[ref121] Wang X.; Zhou J.; Song J.; Liu J.; Xu N.; Wang Z. L. Piezoelectric Field Effect Transistor and Nanoforce Sensor Based on a Single Zno Nanowire. Nano Lett. 2006, 6, 2768–2772. 10.1021/nl061802g. 17163703

[ref122] Zhang Y.; Liu Y.; Wang Z. L. Fundamental Theory of Piezotronics. Adv. Mater. 2011, 23, 3004–3013. 10.1002/adma.201100906. 21560170

[ref123] Luo J.; Fan F. R.; Jiang T.; Wang Z.; Tang W.; Zhang C.; Liu M.; Cao G.; Wang Z. L. Integration of micro-supercapacitors with triboelectric nanogenerators for a flexible self-charging power unit. Nano Res. 2015, 8, 3934–3943. 10.1007/s12274-015-0894-8.

[ref124] Wu W.; Wei Y.; Wang Z. L. Strain-Gated Piezotronic Logic Nanodevices. Adv. Mater. 2010, 22, 4711–4715. 10.1002/adma.201001925. 20681015

[ref125] Yang X.; Hu G.; Gao G.; Chen X.; Sun J.; Wan B.; Zhang Q.; Qin S.; Zhang W.; Pan C.; et al. Coupled Ion-Gel Channel-Width Gating and Piezotronic Interface Gating in Zno Nanowire Devices. Adv. Funct. Mater. 2019, 29, 1807837 10.1002/adfm.201807837.

[ref126] Xu S.; Qin Y.; Xu C.; Wei Y.; Yang R.; Wang Z. L. Self-Powered Nanowire Devices. Nat. Nanotechnol. 2010, 5, 366–373. 10.1038/nnano.2010.46. 20348913

[ref127] Wang Z. L. Nanopiezotronics. Adv. Mater. 2007, 19, 889–892. 10.1002/adma.200602918.

[ref128] Zhong J.; Zhong Q.; Chen G.; Hu B.; Zhao S.; Li X.; Wu N.; Li W.; Yu H.; Zhou J. Surface Charge Self-Recovering Electret Film for Wearable Energy Conversion in a Harsh Environment. Energy Environ. Sci. . 2016, 9, 3085–3091. 10.1039/C6EE02135B.

[ref129] Zhou J.; Gu Y.; Fei P.; Mai W.; Gao Y.; Yang R.; Bao G.; Wang Z. L. Flexible Piezotronic Strain Sensor. Nano Lett. 2008, 8, 3035–3040. 10.1021/nl802367t. 18707178

[ref130] Dai M.; Wang Z.; Wang F.; Qiu Y.; Zhang J.; Xu C.-Y.; Zhai T.; Cao W.; Fu Y.; Jia D.; et al. Two-Dimensional Van Der Waals Materials with Aligned in-Plane Polarization and Large Piezoelectric Effect for Self-Powered Piezoelectric Sensors. Nano Lett. 2019, 19, 5410–5416. 10.1021/acs.nanolett.9b01907. 31343178

[ref131] Wu W.; Wen X.; Wang Z. L. Taxel-Addressable Matrix of Vertical-Nanowire Piezotronic Transistors for Active and Adaptive Tactile Imaging. Science 2013, 340, 952–957. 10.1126/science.1234855. 23618761

[ref132] Yu J.; Gao G.; Huang J.; Yang X.; Han J.; Zhang H.; Chen Y.; Zhao C.; Sun Q.; Wang Z. L. Contact-Electrification-Activated Artificial Afferents at Femtojoule Energy. Nat. Commun. 2021, 12, 1581 10.1038/s41467-021-21890-1. 33707420 PMC7952391

[ref133] Zhang C.; Tang W.; Zhang L.; Han C.; Wang Z. L. Contact Electrification Field-Effect Transistor. ACS Nano 2014, 8, 8702–8709. 10.1021/nn5039806. 25119657

[ref134] Liu Y.; Niu S.; Wang Z. L. Theory of Tribotronics. Adv. Electron. Mater. 2015, 1, 1500124 10.1002/aelm.201500124.

[ref135] Zhang C.; Wang Z. L. Tribotronics—a New Field by Coupling Triboelectricity and Semiconductor. Nano Today 2016, 11, 521–536. 10.1016/j.nantod.2016.07.004.

[ref136] Zou H.; Zhang Y.; Guo L.; Wang P.; He X.; Dai G.; Zheng H.; Chen C.; Wang A. C.; Xu C.; et al. Quantifying the Triboelectric Series. Nat. Commun. 2019, 10, 1427 10.1038/s41467-019-09461-x. 30926850 PMC6441076

[ref137] Gao G.; Wan B.; Liu X.; Sun Q.; Yang X.; Wang L.; Pan C.; Wang Z. L. Tunable Tribotronic Dual-Gate Logic Devices Based on 2d mos2 and Black Phosphorus. Adv. Mater. 2018, 30, 1705088 10.1002/adma.201705088. 29436069

[ref138] Zhang C.; Zhang L. M.; Tang W.; Han C. B.; Wang Z. L. Tribotronic Logic Circuits and Basic Operations. Adv. Mater. 2015, 27, 3533–3540. 10.1002/adma.201501511. 25953435

[ref139] Zhang C.; Li J.; Han C. B.; Zhang L. M.; Chen X. Y.; Wang L. D.; Dong G. F.; Wang Z. L. Organic Tribotronic Transistor for Contact-Electrification-Gated Light-Emitting Diode. Adv. Funct. Mater. 2015, 25, 5625–5632. 10.1002/adfm.201502450.

[ref140] Li J.; Zhang C.; Duan L.; Zhang L. M.; Wang L. D.; Dong G. F.; Wang Z. L. Flexible Organic Tribotronic Transistor Memory for a Visible and Wearable Touch Monitoring System. Adv. Mater. 2016, 28, 106–110. 10.1002/adma.201504424. 26540390

[ref141] Xue F.; Chen L.; Wang L.; Pang Y.; Chen J.; Zhang C.; Wang Z. L. Mos2 Tribotronic Transistor for Smart Tactile Switch. Adv. Funct. Mater. 2016, 26, 2104–2109. 10.1002/adfm.201504485.

[ref142] Khan U.; Kim T.-H.; Ryu H.; Seung W.; Kim S.-W. Graphene Tribotronics for Electronic Skin and Touch Screen Applications. Adv. Mater. 2017, 29, 1603544 10.1002/adma.201603544. 27786382

[ref143] Xi F.; Pang Y.; Li W.; Bu T.; Zhao J.; Liu G.; Guo T.; Liu W.; Zhang C. Tribotronic Bipolar Junction Transistor for Mechanical Frequency Monitoring and Use as Touch Switch. Microsyst. Nanoeng. 2018, 4, 25 10.1038/s41378-018-0026-1. 31057913 PMC6220156

[ref144] Meng Y.; Zhao J.; Yang X.; Zhao C.; Qin S.; Cho J. H.; Zhang C.; Sun Q.; Wang Z. L. Mechanosensation-Active Matrix Based on Direct-Contact Tribotronic Planar Graphene Transistor Array. ACS Nano 2018, 12, 9381–9389. 10.1021/acsnano.8b04490. 30183252

[ref145] Pang Y.; Li J.; Zhou T.; Yang Z.; Luo J.; Zhang L.; Dong G.; Zhang C.; Wang Z. L. Flexible Transparent Tribotronic Transistor for Active Modulation of Conventional Electronics. Nano Energy 2017, 31, 533–540. 10.1016/j.nanoen.2016.11.042.

[ref146] Gao G.; Yu J.; Yang X.; Pang Y.; Zhao J.; Pan C.; Sun Q.; Wang Z. L. Triboiontronic Transistor of Mos2. Adv. Mater. 2019, 31, 1806905 10.1002/adma.201806905. 30589132

[ref147] Zhao J.; Guo H.; Pang Y. K.; Xi F.; Yang Z. W.; Liu G.; Guo T.; Dong G.; Zhang C.; Wang Z. L. Flexible Organic Tribotronic Transistor for Pressure and Magnetic Sensing. ACS Nano 2017, 11, 11566–11573. 10.1021/acsnano.7b06480. 29099579

[ref148] Gao G.; Yu J.; Yang X.; Pang Y.; Zhao J.; Pan C.; Sun Q.; Wang Z. L. Triboiontronic Transistor of Mos2. Adv. Mater. 2019, 31, 1806905 10.1002/adma.201806905. 30589132

[ref149] Wei Y.; Liu W.; Yu J.; Li Y.; Wang Y.; Huo Z.; Cheng L.; Feng Z.; Sun J.; Sun Q.; et al. Triboelectric Potential Powered High-Performance Organic Transistor Array. ACS Nano 2022, 16, 19199–19209. 10.1021/acsnano.2c08420. 36354955

[ref150] Xue F.; Chen L.; Wang L.; Pang Y.; Chen J.; Zhang C.; Wang Z. L. Mos2tribotronic Transistor for Smart Tactile Switch. Adv. Funct. Mater. 2016, 26, 2104–2109. 10.1002/adfm.201504485.

[ref151] Khan U.; Kim T. H.; Ryu H.; Seung W.; Kim S. W. Graphene Tribotronics for Electronic Skin and Touch Screen Applications. Adv. Mater. 2017, 29, 1603544 10.1002/adma.201603544. 27786382

[ref152] Yu J.; Wang Y.; Qin S.; Gao G.; Xu C.; Lin Wang Z.; Sun Q. Bioinspired Interactive Neuromorphic Devices. Mater. Today 2022, 60, 158–182. 10.1016/j.mattod.2022.09.012.

[ref153] Kim Y.; Chortos A.; Xu W.; Liu Y.; Oh J. Y.; Son D.; Kang J.; Foudeh A. M.; Zhu C.; Lee Y.; et al. A Bioinspired Flexible Organic Artificial Afferent Nerve. Science 2018, 360, 998–1003. 10.1126/science.aao0098. 29853682

[ref154] Lumpkin E. A.; Caterina M. J. Mechanisms of Sensory Transduction in the Skin. Nature 2007, 445, 858–865. 10.1038/nature05662. 17314972

[ref155] Wan C.; Chen G.; Fu Y.; Wang M.; Matsuhisa N.; Pan S.; Pan L.; Yang H.; Wan Q.; Zhu L.; et al. An Artificial Sensory Neuron with Tactile Perceptual Learning. Adv. Mater. 2018, 30, 1801291 10.1002/adma.201801291. 29882255

[ref156] Sundaram S. How to Improve Robotic Touch. Science 2020, 370, 768–769. 10.1126/science.abd3643. 33184195

[ref157] Lee Y.; Park J.; Choe A.; Cho S.; Kim J.; Ko H. Mimicking Human and Biological Skins for Multifunctional Skin Electronics. Adv. Funct. Mater. 2020, 30, 1904523 10.1002/adfm.201904523.

[ref158] Kim S. H.; Hong K.; Xie W.; Lee K. H.; Zhang S.; Lodge T. P.; Frisbie C. D. Electrolyte-Gated Transistors for Organic and Printed Electronics. Adv. Mater. 2013, 25, 1822–1846. 10.1002/adma.201202790. 23203564

[ref159] Ji J.; Wang H.; Liu R.; Jiang X.; Zhang Q.; Peng Y.; Sang S.; Sun Q.; Wang Z. L. Dual-Liquid-Gated Electrochemical Transistor and Its Neuromorphic Behaviors. Nano Energy 2021, 87, 106116 10.1016/j.nanoen.2021.106116.

[ref160] Liu W.; Huang Y.; Jin C.; Zhou B.; Yu J.; Liang Y.; Sun Q.; Sun J.; Yang J. Field-Driven Modulating of in-Sn-O Synaptic Transistors with a Precisely Controlled Weight Update. Applied Mater. Today 2021, 23, 101024 10.1016/j.apmt.2021.101024.

[ref161] Yu J.; Qin S.; Zhang H.; Wei Y.; Zhu X.; Yang Y.; Sun Q. Fiber-Shaped Triboiontronic Electrochemical Transistor. Research 2021, 2021, 9840918 10.34133/2021/9840918. 34007968 PMC8098052

[ref162] Xiong Y.; Han J.; Wang Y.; Wang Z. L.; Sun Q. Emerging Iontronic Sensing: Materials, Mechanisms, and Applications. Research 2022, 2022, 9867378 10.34133/2022/9867378. 36072274 PMC9414182

[ref163] Yang X.; Han J.; Yu J.; Chen Y.; Zhang H.; Ding M.; Jia C.; Sun J.; Sun Q.; Wang Z. L. Versatile Triboiontronic Transistor Via Proton Conductor. ACS Nano 2020, 14, 8668–8677. 10.1021/acsnano.0c03030. 32568513

[ref164] Tan F.; Xiong Y.; Yu J.; Wang Y.; Li Y.; Wei Y.; Sun J.; Xie X.; Sun Q.; Wang Z. L. Triboelectric Potential Tuned Dual-Gate Igzo Transistor for Versatile Sensory Device. Nano Energy 2021, 90, 106617 10.1016/j.nanoen.2021.106617.

[ref165] Yu J.; Yang X.; Gao G.; Xiong Y.; Wang Y.; Han J.; Chen Y.; Zhang H.; Sun Q.; Wang Z. L. Bioinspired Mechano-Photonic Artificial Synapse Based on Graphene/Mos2 Heterostructure. Sci. Adv. 2021, 7, eabd9117 10.1126/sciadv.abd9117. 33731346 PMC7968845

[ref166] Lee M.; Bae J.; Lee J.; Lee C.-S.; Hong S.; Wang Z. L. Self-Powered Environmental Sensor System Driven by Nanogenerators. Energy Environ. Sci. 2011, 4, 3359–3363. 10.1039/c1ee01558c.

[ref167] Han J.; Xu N.; Yu J.; Wang Y.; Xiong Y.; Wei Y.; Wang Z. L.; Sun Q. Energy autonomous paper modules and functional circuits. Energy Environ. Sci. 2022, 15, 5069–5081. 10.1039/D2EE02557D.

[ref168] Yang Y.; Zhou Y.; Wu J. M.; Wang Z. L. Single Micro/Nanowire Pyroelectric Nanogenerators as Self-Powered Temperature Sensors. ACS Nano 2012, 6, 8456–8461. 10.1021/nn303414u. 22900676

[ref169] Xu S.; Hansen B. J.; Wang Z. L. Piezoelectric-Nanowire-Enabled Power Source for Driving Wireless Microelectronics. Nat. Commun. 2010, 1, 93 10.1038/ncomms1098. 20981021

[ref170] Murillo G.; Lee M.; Xu C.; Abadal G.; Wang Z. L. Hybrid Resonant Energy Harvester Integrating Zno Nws with Mems for Enabling Zero-Power Wireless Sensor Nodes. Nano Commun. Netw 2011, 2, 235–241. 10.1016/j.nancom.2011.10.001.

[ref171] Dagdeviren C.; Joe P.; Tuzman O. L.; Park K.-I.; Lee K. J.; Shi Y.; Huang Y.; Rogers J. A. Recent Progress in Flexible and Stretchable Piezoelectric Devices for Mechanical Energy Harvesting, Sensing and Actuation. Extreme Mech. Lett. 2016, 9, 269–281. 10.1016/j.eml.2016.05.015.

[ref172] Fernandez S. V.; Cai F.; Chen S.; Suh E.; Tiepelt J.; McIntosh R.; Marcus C.; Acosta D.; Mejorado D.; Dagdeviren C. On-Body Piezoelectric Energy Harvesters through Innovative Designs and Conformable Structures. ACS Biomater. Sci. Eng. 2023, 9, 2070–2086. 10.1021/acsbiomaterials.1c00800. 34735770

[ref173] Jeong C. K.; Lee J.; Han S.; Ryu J.; Hwang G.-T.; Park D. Y.; Park J. H.; Lee S. S.; Byun M.; Ko S. H.; et al. A Hyper-Stretchable Elastic-Composite Energy Harvester. Adv. Mater. 2015, 27, 2866–2875. 10.1002/adma.201500367. 25824939

[ref174] Wu W.; Bai S.; Yuan M.; Qin Y.; Wang Z. L.; Jing T. Lead Zirconate Titanate Nanowire Textile Nanogenerator for Wearable Energy-Harvesting and Self-Powered Devices. ACS Nano 2012, 6, 6231–6235. 10.1021/nn3016585. 22713250

[ref175] Zhao J.; You Z. A Shoe-Embedded Piezoelectric Energy Harvester for Wearable Sensors. Sensors 2014, 14, 12497 10.3390/s140712497. 25019634 PMC4168512

[ref176] Zhong J.; Zhang Y.; Zhong Q.; Hu Q.; Hu B.; Wang Z. L.; Zhou J. Fiber-Based Generator for Wearable Electronics and Mobile Medication. ACS Nano 2014, 8, 6273–6280. 10.1021/nn501732z. 24766072

[ref177] Song Y.; Min J.; Yu Y.; Wang H.; Yang Y.; Zhang H.; Gao W. Wireless Battery-Free Wearable Sweat Sensor Powered by Human Motion. Sci. Adv. 2020, 6, eaay9842 10.1126/sciadv.aay9842. 32998888 PMC7527225

[ref178] Guo H.; Yeh M.-H.; Lai Y.-C.; Zi Y.; Wu C.; Wen Z.; Hu C.; Wang Z. L. All-in-One Shape-Adaptive Self-Charging Power Package for Wearable Electronics. ACS Nano 2016, 10, 10580–10588. 10.1021/acsnano.6b06621. 27934070

[ref179] Yi F.; Wang J.; Wang X.; Niu S.; Li S.; Liao Q.; Xu Y.; You Z.; Zhang Y.; Wang Z. L. Stretchable and Waterproof Self-Charging Power System for Harvesting Energy from Diverse Deformation and Powering Wearable Electronics. ACS Nano 2016, 10, 6519–6525. 10.1021/acsnano.6b03007. 27351212

[ref180] Chen B.; Tang W.; Jiang T.; Zhu L.; Chen X.; He C.; Xu L.; Guo H.; Lin P.; Li D.; et al. Three-Dimensional Ultraflexible Triboelectric Nanogenerator Made by 3d Printing. Nano Energy 2018, 45, 380–389. 10.1016/j.nanoen.2017.12.049.

[ref181] Jiang Q.; Wu C.; Wang Z.; Wang A. C.; He J.-H.; Wang Z. L.; Alshareef H. N. Mxene Electrochemical Microsupercapacitor Integrated with Triboelectric Nanogenerator as a Wearable Self-Charging Power Unit. Nano Energy 2018, 45, 266–272. 10.1016/j.nanoen.2018.01.004.

[ref182] Zou Y.; Tan P.; Shi B.; Ouyang H.; Jiang D.; Liu Z.; Li H.; Yu M.; Wang C.; Qu X. A Bionic Stretchable Nanogenerator for Underwater Sensing and Energy Harvesting. Nat. Commun. 2019, 10, 2695 10.1038/s41467-019-10433-4. 31217422 PMC6584498

[ref183] Gang X.; Guo Z. H.; Cong Z.; Wang J.; Chang C.; Pan C.; Pu X.; Wang Z. L. Textile Triboelectric Nanogenerators Simultaneously Harvesting Multiple “High-Entropy” Kinetic Energies. ACS Appl. Polym. Mater. 2021, 13, 20145–20152. 10.1021/acsami.1c03250. 33878260

[ref184] Cheng R.; Ning C.; Chen P.; Sheng F.; Wei C.; Zhang Y.; Peng X.; Dong K.; Wang Z. L. Enhanced Output of on-Body Direct-Current Power Textiles by Efficient Energy Management for Sustainable Working of Mobile Electronics. Adv. Energy Mater. 2022, 12, 2201532 10.1002/aenm.202201532.

[ref185] Tang W.; Zhou T.; Zhang C.; Ru Fan F.; Bao Han C.; Lin Wang Z. A Power-Transformed-and-Managed Triboelectric Nanogenerator and Its Applications in a Self-Powered Wireless Sensing Node. Nanotechnology 2014, 25, 225402 10.1088/0957-4484/25/22/225402. 24833476

[ref186] Xi F.; Pang Y.; Li W.; Jiang T.; Zhang L.; Guo T.; Liu G.; Zhang C.; Wang Z. L. Universal Power Management Strategy for Triboelectric Nanogenerator. Nano Energy 2017, 37, 168–176. 10.1016/j.nanoen.2017.05.027.

[ref187] Liu W.; Wang Z.; Wang G.; Zeng Q.; He W.; Liu L.; Wang X.; Xi Y.; Guo H.; Hu C.; et al. Switched-Capacitor-Convertors Based on Fractal Design for Output Power Management of Triboelectric Nanogenerator. Nat. Commun. 2020, 11, 1883 10.1038/s41467-020-15373-y. 32312950 PMC7171113

[ref188] Liu Y.; Liu W.; Wang Z.; He W.; Tang Q.; Xi Y.; Wang X.; Guo H.; Hu C. Quantifying Contact Status and the Air-Breakdown Model of Charge-Excitation Triboelectric Nanogenerators to Maximize Charge Density. Nat. Commun. 2020, 11, 1599 10.1038/s41467-020-15368-9. 32221300 PMC7101333

[ref189] Yan W.; Liu Y.; Chen P.; Cao L. N. Y.; An J.; Jiang T.; Tang W.; Chen B.; Wang Z. L. Flexible Film-Discharge-Switch Assisted Universal Power Management System for the Four Operation Modes of Triboelectric Nanogenerators. Adv. Energy Mater. 2022, 12, 2103677 10.1002/aenm.202103677.

[ref190] Guo W.; Zhang X.; Yu X.; Wang S.; Qiu J.; Tang W.; Li L.; Liu H.; Wang Z. L. Self-Powered Electrical Stimulation for Enhancing Neural Differentiation of Mesenchymal Stem Cells on Graphene–Poly(3,4-Ethylenedioxythiophene) Hybrid Microfibers. ACS Nano 2016, 10, 5086–5095. 10.1021/acsnano.6b00200. 27144593

[ref191] Jin Q.; Jiang S.; Zhao Y.; Wang D.; Qiu J.; Tang D.-M.; Tan J.; Sun D.-M.; Hou P.-X.; Chen X.-Q.; et al. Flexible Layer-Structured Bi2te3 Thermoelectric on a Carbon Nanotube Scaffold. Nat. Mater. 2019, 18, 62–68. 10.1038/s41563-018-0217-z. 30455446

[ref192] Han C.-G.; Qian X.; Li Q.; Deng B.; Zhu Y.; Han Z.; Zhang W.; Wang W.; Feng S.-P.; Chen G.; et al. Giant Thermopower of Ionic Gelatin near Room Temperature. Science 2020, 368, 1091–1098. 10.1126/science.aaz5045. 32354840

[ref193] Hong S.; Gu Y.; Seo J. K.; Wang J.; Liu P.; Meng Y. S.; Xu S.; Chen R. Wearable thermoelectrics for personalized thermoregulation. Sci. Adv. 2019, 5, eaaw0536 10.1126/sciadv.aaw0536. 31114803 PMC6524982

[ref194] Lee B.; Cho H.; Park K. T.; Kim J.-S.; Park M.; Kim H.; Hong Y.; Chung S. High-Performance Compliant Thermoelectric Generators with Magnetically Self-Assembled Soft Heat Conductors for Self-Powered Wearable Electronics. Nat. Commun. 2020, 11, 5948 10.1038/s41467-020-19756-z. 33230141 PMC7684283

[ref195] Byun S.-H.; Kim C. S.; Agno K.-C.; Lee S.; Li Z.; Cho B. J.; Jeong J.-W. Design Strategy for Transformative Electronic System toward Rapid, Bidirectional Stiffness Tuning Using Graphene and Flexible Thermoelectric Device Interfaces. Adv. Mater. 2021, 33, 2007239 10.1002/adma.202007239. 33491832

[ref196] Kaltenbrunner M.; Adam G.; Głowacki E. D.; Drack M.; Schwödiauer R.; Leonat L.; Apaydin D. H.; Groiss H.; Scharber M. C.; White M. S.; et al. Flexible High Power-Per-Weight Perovskite Solar Cells with Chromium Oxide–Metal Contacts for Improved Stability in Air. Nat. Mater. 2015, 14, 1032–1039. 10.1038/nmat4388. 26301766

[ref197] Cheng Y.-B.; Pascoe A.; Huang F.; Peng Y. Print Flexible Solar Cells. Nature 2016, 539, 488–489. 10.1038/539488a. 27882991

[ref198] Jinno H.; Fukuda K.; Xu X.; Park S.; Suzuki Y.; Koizumi M.; Yokota T.; Osaka I.; Takimiya K.; Someya T. Stretchable and Waterproof Elastomer-Coated Organic Photovoltaics for Washable Electronic Textile Applications. Nat. Energy 2017, 2, 780–785. 10.1038/s41560-017-0001-3.

[ref199] Kaltenbrunner M.; White M. S.; Głowacki E. D.; Sekitani T.; Someya T.; Sariciftci N. S.; Bauer S. Ultrathin and Lightweight Organic Solar Cells with High Flexibility. Nat. Commun. 2012, 3, 770 10.1038/ncomms1772. 22473014 PMC3337988

[ref200] Wu S.; Li Z.; Zhang J.; Wu X.; Deng X.; Liu Y.; Zhou J.; Zhi C.; Yu X.; Choy W. C. H.; et al. Low-Bandgap Organic Bulk-Heterojunction Enabled Efficient and Flexible Perovskite Solar Cells. Adv. Mater. 2021, 33, 2105539 10.1002/adma.202105539. 34601764

[ref201] Wan J.; Xia Y.; Fang J.; Zhang Z.; Xu B.; Wang J.; Ai L.; Song W.; Hui K. N.; Fan X.; et al. Solution-Processed Transparent Conducting Electrodes for Flexible Organic Solar Cells with 16.61% Efficiency. Nano-Micro Lett. 2021, 13, 44 10.1007/s40820-020-00566-3. PMC8187532 34138225

[ref202] Kim S.; Oh H.; Kang G.; Han I. K.; Jeong I.; Park M. High-Power and Flexible Indoor Solar Cells Via Controlled Growth of Perovskite Using a Greener Antisolvent. ACS Appl. Energy Mater. 2020, 3, 6995–7003. 10.1021/acsaem.0c00997.

[ref203] Huang J.; Ren Z.; Zhang Y.; Liu K.; Zhang H.; Tang H.; Yan C.; Zheng Z.; Li G. Stretchable Ito-Free Organic Solar Cells with Intrinsic Anti-Reflection Substrate for High-Efficiency Outdoor and Indoor Energy Harvesting. Adv. Funct. Mater. 2021, 31, 2010172 10.1002/adfm.202010172.

[ref204] Bandodkar A. J.; You J.-M.; Kim N.-H.; Gu Y.; Kumar R.; Mohan A. M. V.; Kurniawan J.; Imani S.; Nakagawa T.; Parish B.; et al. Soft, Stretchable, High Power Density Electronic Skin-Based Biofuel Cells for Scavenging Energy from Human Sweat. Energy Environ. Sci. 2017, 10, 1581–1589. 10.1039/C7EE00865A.

[ref205] Bandodkar A. J.; Gutruf P.; Choi J.; Lee K.; Sekine Y.; Reeder J. T.; Jeang W. J.; Aranyosi A. J.; Lee S. P.; Model J. B. Battery-Free, Skin-Interfaced Microfluidic/Electronic Systems for Simultaneous Electrochemical, Colorimetric, and Volumetric Analysis of Sweat. Sci. Adv. 2019, 5, eaav3294 10.1126/sciadv.aav3294. 30746477 PMC6357758

[ref206] Tang S.; Zhang F.; Gong H.; Wei F.; Zhuang J.; Karshalev E.; Esteban-Fernández de Ávila B.; Huang C.; Zhou Z.; Li Z.; et al. Enzyme-Powered Janus Platelet Cell Robots for Active and Targeted Drug Delivery. Sci. Robot. 2020, 5, eaba6137 10.1126/scirobotics.aba6137. 33022613

[ref207] Xu C.; Wang X.; Wang Z. L. Nanowire Structured Hybrid Cell for Concurrently Scavenging Solar and Mechanical Energies. J. Am. Chem. Soc. 2009, 131, 5866–5872. 10.1021/ja810158x. 19338339

[ref208] Zhao C.; Zhang Q.; Zhang W.; Du X.; Zhang Y.; Gong S.; Ren K.; Sun Q.; Wang Z. L. Hybrid piezo/triboelectric nanogenerator for highly efficient and stable rotation energy harvesting. Nano Energy 2019, 57, 440–449. 10.1016/j.nanoen.2018.12.062.

[ref209] Seo B.; Cha Y.; Kim S.; Choi W. Rational Design for Optimizing Hybrid Thermo-triboelectric Generators Targeting Human Activities. ACS Energy Lett. 2019, 4, 2069 10.1021/acsenergylett.9b01426.

[ref210] Frackowiak E. Carbon Materials for Supercapacitor Application. Phys. Chem. Chem. 2007, 9, 1774–1785. 10.1039/b618139m. 17415488

[ref211] Iro Z. S.; Subramani C.; Dash S. J. I. J. E. S. A Brief Review on Electrode Materials for Supercapacitor. Int. J. Electrochem. Sci. 2016, 11, 10628–10643. 10.20964/2016.12.50.

[ref212] Le V. T.; Kim H.; Ghosh A.; Kim J.; Chang J.; Vu Q. A.; Pham D. T.; Lee J.-H.; Kim S.-W.; Lee Y. H. Coaxial Fiber Supercapacitor Using All-Carbon Material Electrodes. ACS Nano 2013, 7, 5940–5947. 10.1021/nn4016345. 23731060

[ref213] Shown I.; Ganguly A.; Chen L.-C.; Chen K.-H. Conducting Polymer-Based Flexible Supercapacitor. Energy Sci. Eng. 2015, 3, 2–26. 10.1002/ese3.50.

[ref214] Pasta M.; La Mantia F.; Hu L.; Deshazer H. D.; Cui Y. Aqueous Supercapacitors on Conductive Cotton. Nano Res. 2010, 3, 452–458. 10.1007/s12274-010-0006-8.

[ref215] Huang L.; Santiago D.; Loyselle P.; Dai L. Graphene-Based Nanomaterials for Flexible and Wearable Supercapacitors. Small 2018, 14, 1800879 10.1002/smll.201800879. 30009468

[ref216] Manjakkal L.; Pullanchiyodan A.; Yogeswaran N.; Hosseini E. S.; Dahiya R. A Wearable Supercapacitor Based on Conductive Pedot:Pss-Coated Cloth and a Sweat Electrolyte. Adv. Mater. 2020, 32, 1907254 10.1002/adma.201907254. 32390218

[ref217] Xue Q.; Sun J.; Huang Y.; Zhu M.; Pei Z.; Li H.; Wang Y.; Li N.; Zhang H.; Zhi C. Recent Progress on Flexible and Wearable Supercapacitors. Small 2017, 13, 1701827 10.1002/smll.201701827. 28941073

[ref218] Ren J.; Zhang Y.; Bai W.; Chen X.; Zhang Z.; Fang X.; Weng W.; Wang Y.; Peng H. Elastic and Wearable Wire-Shaped Lithium-Ion Battery with High Electrochemical Performance. Angew. Chem., Int. Ed. 2014, 126, 7998–8003. 10.1002/ange.201402388. 24899361

[ref219] Zhang Y.; Zhao Y.; Ren J.; Weng W.; Peng H. Advances in Wearable Fiber-Shaped Lithium-Ion Batteries. Adv. Mater. 2016, 28, 4524–4531. 10.1002/adma.201503891. 26643467

[ref220] Kwon Y. H.; Woo S.-W.; Jung H.-R.; Yu H. K.; Kim K.; Oh B. H.; Ahn S.; Lee S.-Y.; Song S.-W.; Cho J.; et al. Cable-Type Flexible Lithium Ion Battery Based on Hollow Multi-Helix Electrodes. Adv. Mater. 2012, 24, 5192–5197. 10.1002/adma.201202196. 22886776

[ref221] Pu X.; Li L.; Song H.; Du C.; Zhao Z.; Jiang C.; Cao G.; Hu W.; Wang Z. L. A Self-Charging Power Unit by Integration of a Textile Triboelectric Nanogenerator and a Flexible Lithium-Ion Battery for Wearable Electronics. Adv. Mater. 2015, 27, 2472–2478. 10.1002/adma.201500311. 25736078

[ref222] He J.; Lu C.; Jiang H.; Han F.; Shi X.; Wu J.; Wang L.; Chen T.; Wang J.; Zhang Y.; et al. Scalable Production of High-Performing Woven Lithium-Ion Fibre Batteries. Nature 2021, 597, 57–63. 10.1038/s41586-021-03772-0. 34471277

[ref223] Liu Q.-C.; Liu T.; Liu D.-P.; Li Z.-J.; Zhang X.-B.; Zhang Y. A Flexible and Wearable Lithium–Oxygen Battery with Record Energy Density Achieved by the Interlaced Architecture Inspired by Bamboo Slips. Adv. Mater. 2016, 28, 8413–8418. 10.1002/adma.201602800. 27479932

[ref224] Wang Y.; Chen C.; Xie H.; Gao T.; Yao Y.; Pastel G.; Han X.; Li Y.; Zhao J.; Fu K.; et al. 3d-Printed All-Fiber Li-Ion Battery toward Wearable Energy Storage. Adv. Funct. Mater. 2017, 27, 1703140 10.1002/adfm.201703140.

[ref225] Rao J.; Liu N.; Zhang Z.; Su J.; Li L.; Xiong L.; Gao Y. All-Fiber-Based Quasi-Solid-State Lithium-Ion Battery Towards Wearable Electronic Devices with Outstanding Flexibility and Self-Healing Ability. Nano Energy 2018, 51, 425–433. 10.1016/j.nanoen.2018.06.067.

[ref226] Yi F.; Ren H.; Shan J.; Sun X.; Wei D.; Liu Z. Wearable Energy Sources Based on 2d Materials. Chem. Soc. Rev. 2018, 47, 3152–3188. 10.1039/C7CS00849J. 29412208

[ref227] Liu R.; Wang Z. L.; Fukuda K.; Someya T. Flexible Self-Charging Power Sources. Nat. Rev. Mater. 2022, 7, 870–886. 10.1038/s41578-022-00441-0.

[ref228] Choi Y. S.; Jeong H.; Yin R. T.; Avila R.; Pfenniger A.; Yoo J.; Lee J. Y.; Tzavelis A.; Lee Y. J.; Chen S. W.; et al. A Transient, Closed-Loop Network of Wireless, Body-Integrated Devices for Autonomous Electrotherapy. Science 2022, 376, 1006–1012. 10.1126/science.abm1703. 35617386 PMC9282941

[ref229] Kwon K.; Kim J. U.; Won S. M.; Zhao J.; Avila R.; Wang H.; Chun K. S.; Jang H.; Lee K. H.; Kim J.-H. A Battery-Less Wireless Implant for the Continuous Monitoring of Vascular Pressure, Flow Rate and Temperature. Nat. Biomed. Eng. 2023. 10.1038/s41551-023-01022-4 37037964

[ref230] Jiang Y.; Trotsyuk A. A.; Niu S.; Henn D.; Chen K.; Shih C.-C.; Larson M. R.; Mermin-Bunnell A. M.; Mittal S.; Lai J.-C.; et al. Wireless, Closed-Loop, Smart Bandage with Integrated Sensors and Stimulators for Advanced Wound Care and Accelerated Healing. Nat. Biotechnol. 2023, 41, 652–662. 10.1038/s41587-022-01528-3. 36424488

[ref231] Kalidasan V.; Yang X.; Xiong Z.; Li R. R.; Yao H.; Godaba H.; Obuobi S.; Singh P.; Guan X.; Tian X.; et al. Wirelessly Operated Bioelectronic Sutures for the Monitoring of Deep Surgical Wounds. Nat. Biomed. Eng. 2021, 5, 1217–1227. 10.1038/s41551-021-00802-0. 34654900

[ref232] Silverå Ejneby M.; Jakešová M.; Ferrero J. J.; Migliaccio L.; Sahalianov I.; Zhao Z.; Berggren M.; Khodagholy D.; Đerek V.; Gelinas J. N.; et al. Chronic Electrical Stimulation of Peripheral Nerves Via Deep-Red Light Transduced by an Implanted Organic Photocapacitor. Nat. Biomed. Eng. 2022, 6, 741–753. 10.1038/s41551-021-00817-7. 34916610

[ref233] Kim Y.; Suh J. M.; Shin J.; Liu Y.; Yeon H.; Qiao K.; Kum H. S.; Kim C.; Lee H. E.; Choi C.; et al. Chip-Less Wireless Electronic Skins by Remote Epitaxial Freestanding Compound Semiconductors. Science 2022, 377, 859–864. 10.1126/science.abn7325. 35981034

[ref234] Park S.; Heo S. W.; Lee W.; Inoue D.; Jiang Z.; Yu K.; Jinno H.; Hashizume D.; Sekino M.; Yokota T.; et al. Self-Powered Ultra-Flexible Electronics Via Nano-Grating-Patterned Organic Photovoltaics. Nature 2018, 561, 516–521. 10.1038/s41586-018-0536-x. 30258137

[ref235] Huang Z.; Hao Y.; Li Y.; Hu H.; Wang C.; Nomoto A.; Pan T.; Gu Y.; Chen Y.; Zhang T.; et al. Three-Dimensional Integrated Stretchable Electronics. Nat. Electron. 2018, 1, 473–480. 10.1038/s41928-018-0116-y.

